# Why does library holding format really matter for book impact assessment?: Modelling the relationship between citations and altmetrics with print and electronic holdings

**DOI:** 10.1007/s11192-021-04239-9

**Published:** 2021-12-20

**Authors:** Ashraf Maleki

**Affiliations:** University of Turku, Turku, Finland

**Keywords:** Scholarly books, Book impact, Library holding, Library print holding, Library eholding, Library electronic holding, Citation analysis, Altmetric, Google books, Syllabus mentions, OCLC, WorldCat, Twitter, Mendeley readership, Facebook, Wikipedia, Blog pages, News posts

## Abstract

Scholarly books are important outputs in some fields and their many publishing formats seem to introduce opportunities to scrutinize their impact. As there is a growing interest in the publisher-enforced massive collection of ebooks in libraries in the past decade, this study examined how this influences the relationship that library print holdings (LPH), library electronic holdings (LEH) and total library holdings (TLH) have with other metrics. As a follow up study to a previous research on OCLC library holdings, the relationship between library holdings and twelve other metrics including Scopus Citations, Google Books (GB) Citations, Goodreads engagements, and Altmetric indicators were examined for 119,794 Scopus-indexed book titles across 26 fields. Present study confirms the weak correlation levels observed between TLH and other indicators in previous studies and contributes additional evidence that print holdings can moderately reflect research, educational and online impact of books consistently more efficient than eholdings and total holdings across fields and over time, except for Mendeley for which eholdings slightly prevailed. Regression models indicated that along with other dimensions, Google Books Citations frequently best explained LPH (in 14 out of 26 fields), whereas Goodreads User counts were weak, but the best predictor of both LEH and TLH (in 15 fields out of 26), suggesting significant association of eholdings with online uptake of books. Overall, findings suggest that inclusion of eholdings overrides the more impactful counts of print holdings in Total Library Holdings metric and therefore undermines the statistical results, whilst print holdings has both statistically and theoretically promising underlying assumptions for prediction of impact of books and shows greater promise than the general Library Holding metric for book impact assessment. Thus, there is a need for a distinction between print and electronic holding counts to be made, otherwise total library holding data need to be interpreted with caution.

**Supplementary Information:**

The online version contains supplementary material available at 10.1007/s11192-021-04239-9.

## Introduction

Books are important scholarly outputs in Social Science and Humanities and their many publishing formats (print and various electronic formats) introduce opportunities to investigate their various aspects of impact. As quantifying impact in humanities is important (Nederhof, 2006; Torres-Salinas & Moed, 2009) current research concerns with quantifying impact of books across their various publishing formats. Libraries are the major source of data for academic books and most scholars seek to obtain the materials they need from their institutional library rather than a bookstore. Library Holding (LH) refers to the number of libraries that have acquired a certain book title in their collection. A recent study into the WorldCat (a significant source of library holding statistics) has indicated that U.S. libraries (about 63%) are predominant among an overall 15,195 libraries worldwide, of which 38% (5804) account for academic libraries, 29% (4441) public libraries and 33% (4950) other libraries (Torres-Salinas & Arroyo-Machado, 2020). This suggests that there can be stronger academic theme in WorldCat’s library holding counts for scholarly books.

Two different studies in 2009 introduced library holdings as a potentially useful indicator for assessment of book impact, one of them calling it *Libcitations* short for Library Citations (White et al., 2009) and the other naming it *Catalog Inclusions* (Torres-Salinas & Moed, 2009). It is introduced as an indicator of books’ acquisition worth from librarians’ point of view who choose titles based on institutional and community requests. As shown in an earlier investigation into publication format of books in libraries (Maleki, 2021), broad coverage is the major reason for importance of library holding statistics for books in WorldCat’s OCLC (Online Computer Library Centre). Work by Torres-Salinas and Moed (2009) and White et al. (2009) have made a significant contribution to the area of book impact assessment. However, ever since they have introduced library holdings as an indicator of book impact, the feature of Work Format in WorldCat, which is one of the central services for Library Management and the underlying factor in calculating holding statistics, has not been investigated (Maleki, 2021).

One important reason for significance of book format is that academic libraries have shown a growing interest to acquire ebooks in the past decade, particularly in the U.S. (Romano, 2016). WorldCat provides access to over 50 million records from 729 ebook providers through users’ libraries^1^ and the number of libraries having access to ebook records is mentioned as eholdings in OCLC API results. Admittedly, there are good reasons for libraries to provide digital version of books; ebooks are key to sustaining library service at such times as current COVID-19 pandemic by automatically removing the need for physical presence of users. However, for about a decade, what we know about library holdings is largely based upon empirical studies that have extensively used Total Library Holdings for book impact assessment which is indirectly representing the sum of counts of library print and electronic holdings. A major problem with this kind of application is that it does not take into account the different methods in which print and electronic books are acquired for libraries.

Libraries may order ebooks based on specific packages from publishers, which means that some books without actual demand can also find their way into holding statistics. However, as it also is not clear how much library print book orders are made based upon real information need, various studies have assessed library book circulation for print (Cabezas-Clavijo et al., 2013) and ebooks (Cox, 2011) or both (e.g. Haugh, 2016). Considering this, it would be reasonable to raise concerns about how library holding statistics need to be interpreted in terms of impact. Print books constitute a larger proportion of the libraries’ costs and space. Library holding counts as well as library loan statistics (Cabezas-Clavijo et al., 2013) are perhaps the most popular methods to track impact of print book collection. Other than inter-library loans, books provided via any acquisition model are meant to become a perpetual part of libraries. Perpetual access translates into higher expenses, but also a more careful selection policy. The major challenge would be with ebooks in libraries as not all types of access to them is perpetual. Majority of academic libraries in the U.S. provide them via subscription (79% of libraries) perhaps to be economical (Kont, 2016), and then, title by title purchases (75%), demand-driven purchases (49%), upfront purchases (34%), demand-driven short-term loans (18%), ebook approval plans (11%) and combined ebook and print approval plans (10%) (Romano, 2016). Thus, regardless of demand for all titles, there are substantially larger number of libraries having access to them. It would, therefore, be reasonable to question and investigate the usefulness of ebook holdings presence in library holding counts as an indicator of book impact assessment. This research addresses the questions of whether and to what extent ebooks and print books in libraries are in line with academic, educational, and cultural needs for books in academia and society.

A discussion about the problem of massive collections of ebooks has emerged recently in an analysis of WorldCat Identities (WI) for author level indicator of book holdings, where 398 most prolific book authors in information science were identified with false assignment of book titles and thus an inordinate number of holding counts that originated from ebooks (Torres-Salinas, Arroyo-Machado and Thelwall, 2020). A previous investigation into print and electronic holdings also showed that the average number of electronic holdings for each title is about 7 times more numerous than print holdings (Maleki, 2021), showing that a significant part of electronic holding is included in total library holding counts. However, it has not been investigated what sort of implications these differences in provision of the print and electronic books have for impact assessment.

Current research is a follow up study to my previous investigation on comparison of fourteen book metrics (Maleki, 2021). This research will use 119,794 Scopus-indexed books in 26 fields by libraries across work formats and other metric counts and over time to investigate relationships between these metrics. Correlation test provides initial evidence of potential connection between indicators. The perception behind testing the correlation between library holdings for different publication formats of books and other indicators is to examine the extent to which provision of each publication format represent library mission in covering needs for books. Thus, current research aims to identify the relationship that exists (Sud & Thelwall, 2014) between library holdings of each publication format with formal citations to books and other metrics in order to clarify aspects of impact. The major notion in this research is that because print books and ebooks are acquired by libraries in different methods and to varying extent, it is possible that print and ebook holdings reveal various aspects of impact, hidden from previous statistical correlation studies that used total holding counts. Thus, current research will investigate the difference between library print holdings, library electronic holdings and total library holdings in the empirical relationship they have with various dimensions of book impact including formal citations from Scopus and Google Books as sources of research related impact (Kousha & Thelwall, 2009, 2015; Kousha et al., 2011), syllabus mentions as a source of educational impact (Kousha & Thelwall, 2016), Goodreads captures as an indicator of cultural impact (Zuccala et al., 2015; Kousha et al., 2017), and altmetric indicators as sources of social media uptake of books (Thelwall et al., 2013).

As metrics for assessing books are relatively numerous and less known for the aspect of impact they can measure, it would be valuable to learn which metrics perform strongly when statistically explaining LH metrics. Except a few metrics such as syllabus mention that clearly measures educational uptake of books, most assessment metrics such as online social media metrics, however, have a multi-dimensional nature and might be driven for variety of social, educational and scholarly reasons. In this respect, some metrics perform stronger than others in revealing a certain aspect of impact such as Mendeley readerships which might indicate scholarly uptake as well as simple readership (Mohammadi et al., 2016). Although correlation analysis helps to identify this type of metrics usage, correlation only shows relationship between pairs of metrics and might not help to identify the predominant aspect of metrics usage when they are frequently used for different purposes. In order to take multi-dimensional factors of metrics into accounts and reveal metrics which are best aligned with LH metrics, there is a need for a regression analysis that uses normalized form of all metrics without excluding documents that have zero metric counts. This will help acquiring a universal understanding of metrics strength in predicting each one of LH metrics for different subject fields.

Furthermore, as uptake of books by library holdings metric is comprehensive and substantially larger than any other book metric in a short time after books’ first edition publication (Maleki, 2021), this research also intends to explore the predictability of various aspects of book impact using print and electronic holding counts over time.

## Research questions

The aim of this study is to ascertain the usefulness of book publication format in libraries for book impact assessment. In order to address this goal, correlations between holding counts across print and electronic formats and other metrics are investigated and regression models for predicting metrics with print and electronic book holdings in libraries across disciplines and over time are offered. This research, thus, seeks to address the following questions:

1. Do library print and electronic holdings correlate with other book metrics?
2. Which aspect of impact is best predicted by print, electronic and total library holdings?
3. Is there a significant statistical benefit to use library print holdings instead of library eholdings or total library holdings?
4. To what extent can all book metrics in the study statistically explain Library Holdings across its format?
5. How are the patterns in above questions changed across disciplines and over time?

## Background

### Correlations between library holdings and other metrics

The relationship between library holdings and many other indicators is already tested in different studies and reviewed in Torres-Salinas and Arroyo-Machado (2020). Previous studies have used it to indicate whether library holding counts can indicate other aspects of impact. For instance, Zuccala et al. (2021) associated library holdings with visibility of academic books and found that the publisher levels (nationality and internationality) do not have a predictive value for this kind of visibility. Table 1 suggests the strongest correlation results reported in different studies. In relation with formal citations, library holding counts have shown significant weak or low coefficients at moderate level (max *r* = 0.4 in Linmans, 2010) (more in Table 1). However, the strongest coefficients with non-WorldCat metrics were seen between library holdings and Goodreads ratings (*r* = 0.467) and Goodreads reviews (*r* = 0.448) (Zuccala et al., 2015). Overall, these studies have used the evidence of weak and low moderate coefficients to suggest that library holdings reflect aspects of impact different from both citations and other assessment metrics.

**Table 1:** The strongest significant correlation coefficients between Library Holdings and other metrics from different studies

Metrics	Linmans (2010)	Kousha (2011)	Zuccala (2015)	Kousha (2015)	Kousha (2017)	Torres-Salinas (2017)	Zuccala (2021)
Scopus citations			0.208 ARL			0.008	0.124
BKCI citations	0.40 En	0.145 SS			0.235 H 2010		
Google books citations		0.268 A&H		0.326 S&T	0.243 SS 2008		
Google scholar citations							0.423
Syllabus mentions				0.121			
Amazon reviews		0.348 A&H				− 0.006	
Choice rating score				0.304 H			
Mendeley captures		0.072 SS				0.123	
Goodreads captures/engagements			0.467 nonARL		0.396 A 2008	− 0.006	
Goodreads reviews			0.448 nonARL		0.346 SS 2008	− 0.006	
Goodreads average rating					0.263 SS 2008		
Wikipedia links					0.387 A 2010	0.071	
EBSCO saves						0.092	
EBSCO abstract views						0.129	
EBSCO PDF views						0.241	
EBSCO HTML views						0.146	
Twitter						− 0.001	
PlumX usage							0.246
PlumX captures							0.426
WorldCat country distribution							0.959

En: Books in English; SS: Social Science; A&H: Art and Humanities; S&T: Science and Technology; ARL: Association of Research Libraries

Linmans (2010) 1135 English and non-English books of 264 faculty members of the Faculty of Humanities at Leiden University

Kousha et al. (2011) 2739 monographs indexed in BKCI from 2008

Zuccala et al. (2015) 997 book titles in popular and academic History published up to 2011

Kousha and Thelwall (2015) 451 Choice-reviewed books multidisciplinary

Kousha and Thelwall (2017) 18,735 Scopus monographs in 2005–2012, multidisciplinary

Torres-Salinas et al. (2017) 268,081 EBSCO books multi-language and disciplinary

Zuccala et al. (2021) 743 academic books published in 2017 and authored by Danish, Finnish and Norwegian university researchers

Some general patterns could also be followed in the correlation results in terms of subject area and publication date. For instance, library holdings have often shown stronger correlation coefficients in Social Science and Art and Humanities than Medicine and Physical Science with various metrics such as formal citations, Amazon Reviews, and Goodreads Reviews (e.g. Kousha et al., 2011; Kousha & Thelwall, 2017) (more in Table 1). A few studies have also concerned to apply publication year into the correlation analyses. Older books have often shown stronger correlation coefficient with library holdings than more recent books (Kousha & Thelwall, 2017). One study on books holdings in Association of Research Libraries assessed the correlation between citations and library holdings in fields of History and Literature for books published in 1996–2000 and 2007–2011. In both fields, the later time period showed a weaker relationship (Zuccala & White, 2015). Kousha et al. (2016) also similarly reported a reduction in strength of correlation coefficients in more recent years across many fields. None of the above-mentioned studies have used publication year of first edition of books instead of the publication date of publishers, although they have reported elimination of duplicate editions.

Some recent studies, have used the number of library holdings across regions and various geographies using entropy method, reporting significant weak Spearman’s correlation coefficients with *Choice* reviews in recommendation, readership and interdisciplinary subject levels. One researcher suggested that WorldCat country distributions with *r* = 0.959 (*p* < 0.01) is in strong connection with the Library holdings (Zuccala et al., 2021). Another study reported the maximum *r* = 0.407 between distribution of holdings and *Choice* readerships in Science and Technology books (Zhou & Zhang, 2020b). In two other studies, they have shown a weak correlation between a compound score based on width and breadth score of books and holding numbers at *r* = 0.050 (Zhang & Zhou, 2020) and slightly better correlations for reference books at *r* = 0.209 (Zhou & Zhang, 2020a), both significant at *p* = 0.01.

However, correlation results from previous studies need to be considered carefully; firstly, because the relationship has not been examined for library holding counts across book formats; and secondly, due to the differences of examined datasets. The datasets used for correlation analysis sometimes varied in terms of inclusion or exclusion of books with zero metric counts. In other words, some studies used books with at least one of the respective events as mentioned in Table 1 and excluded books that have never been cited, mentioned, or reviewed (e.g., Kousha & Thelwall, 2015; Zuccala et al., 2015). Exclusion of books with zero metric counts can lead to different correlation coefficients from when they are included (Haustein et al., 2015). Some studies have examined the correlation analysis only for books meeting certain criteria. For instance, the correlation analyses in Zuccala et al. (2015) were conducted on 997 popular and academic History books at Scopus citations (≥ 4) and Goodreads Ratings (≥ 10) above the 75th percentile across two academic library holdings from ARL (American Association of Research Libraries) and non-ARL (International academic libraries). In the present study, in order to make research results comparable with previous studies, correlation coefficient has been reported for both zeros included and only non-zero datasets of books. A case comparison is also incorporated in the discussion. Furthermore, to indicate the usefulness of work format-based library holdings count, all correlations for print and eholdings are compared with total holdings.

## Research method

### Dataset

**Fig. 1:**
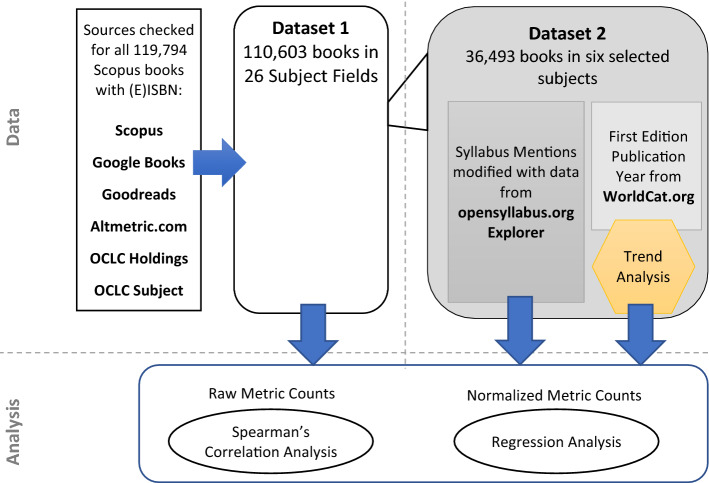
The process of data collection and analysis

This research uses two datasets from an earlier research (Maleki, 2021). The research design was planned to scrutinize differences in library print and electronic holdings with each other and with total library holdings in terms of impact. The aim was to examine the relationship that library print and electronic holdings have with citations, and other book-specific indicators. Throughout this paper, the abbreviations **LPH**, **LEH** and **TLH** are used to refer to *Library Print Holdings*, *Library Electronic Holdings* and *Total Library Holdings*, respectively. Book data are drawn from seven main sources: (1) Scopus, (2) OCLC (classify.oclc.org); (3) WorldCat (worldcat.org); (4) Altmetric.com; (5) Open Syllabus Project (opensyllabus.org); (6) Google Books (books.google.com); and (7) Goodreads (goodreads.com). Figure 1 gives an overview of the research process in terms of data and analysis. A complete and a sample dataset are selected to investigate the research questions:

- Dataset 1 consists of 119,794 unique titles with ISBN or E-ISBN of which 110,603 titles had a Library of Congress Subject Class (LCC) in OCLC (Tables 2, 3, adopted from Maleki, 2021)
- Dataset 2 involved all titles in six subject categories (Anthropology, Arts, Business and Economics, Law, Medicine and Political Science) from Dataset 1. This sample is selected for further analyses by: (a) The first edition’s publication date of books in WorldCat and (b) Educational impact of books that is manually extracted from *opensyllabus.org*. The major criteria for selecting the subjects are importance of books in those fields.

**Table 2:** Proportion cited of books in eight (out of 14) metrics across 26 fields (Adopted from Maleki, 2021)

Fields\Metrics	LPH	LEH	Scopus	GB	SM (OS)	GU	GR	# of Books
Agriculture	99.9%	99.0%	70.2%	70.3%		43.8%	23.5%	1775
Anthropology*	100.0%	99.6%	77.3%	83.6%	44.9%	64.6%	46.0%	1198
Arts*	100.0%	98.6%	61.8%	71.0%	33.4%	64.9%	42.5%	1245
Business and economics*	99.8%	99.5%	66.2%	74.6%	30.5%	50.7%	31.5%	11,987
Chemistry	99.4%	98.5%	80.1%	67.7%		29.1%	10.8%	1651
Education	99.9%	99.9%	63.3%	77.8%		50.5%	32.2%	4475
Engineering and technology	99.5%	93.2%	69.5%	62.8%		33.4%	16.3%	14,140
Ethics and religion	99.6%	99.9%	69.4%	84.9%		72.3%	54.5%	4354
Geography	99.6%	98.0%	72.4%	78.1%		48.8%	27.7%	1367
History	99.5%	99.6%	70.7%	83.0%		71.4%	53.2%	8,798
Languages and literature	99.8%	99.8%	71.5%	79.7%		68.1%	47.4%	9798
Law*	99.8%	99.8%	67.0%	79.4%	35.8%	52.6%	28.5%	4734
Library science and bibliography	100.0%	100.0%	63.5%	70.7%		62.9%	46.8%	563
Mathematics	99.5%	98.8%	73.4%	65.1%		47.8%	29.9%	4093
Medicine*	98.9%	99.5%	62.2%	62.1%	25.6%	45.7%	25.8%	12,227
Military science	99.4%	99.5%	58.2%	69.7%		52.6%	34.5%	822
Music	99.8%	100.0%	68.3%	79.3%		68.3%	51.7%	1161
Natural history and biology	99.8%	98.3%	73.3%	69.1%		48.7%	29.1%	1640
Philosophy	99.9%	100.0%	72.3%	85.0%		72.6%	52.5%	1935
Physics	99.8%	97.7%	72.4%	63.7%		44.2%	22.6%	2628
Physiology	99.4%	99.4%	66.9%	60.5%		41.0%	20.2%	1716
Political sciences*	99.9%	99.9%	71.8%	82.4%	42.6%	56.8%	35.5%	5098
Psychology	99.8%	99.8%	67.5%	79.4%		63.1%	45.2%	1996
Recreation and leisure	99.7%	99.7%	69.8%	79.6%		67.6%	51.2%	777
Social sciences	99.8%	99.8%	71.1%	79.8%		58.3%	39.4%	9645
Zoology	99.6%	99.1%	74.0%	67.9%		54.4%	37.0%	758
Unknown	99.6%	99.1%	64.2%	69.1%		37.7%	23.4%	9190
*Average of 26 fields (excluding Unknown)*	**99.70%**	**99.11%**	**69.39%**	**74.12%**		**55.17%**	**35.98%**	**110,603**
Average of six* fields	**99.74%**	**99.47%**	**67.72%**	**75.52%**	**35.46%**	**55.90%**	**34.97%**	**36,493**

LPH: Library Print Holdings; LEH: Library Electronic Holdings; TLH: Total Library Holdings; GB: Google Books; SM (OS): Syllabus Mentions from opensyllabus.org; GU: Goodreads Users; GR: Goodreads Ratings

**Table 3:** Proportion cited of books in six (out of 14) metrics across 26 fields (Adopted from Maleki, 2021)

Fields\metrics	GTR	Mendeley	Twitter	Facebook	Wikipedia	Blogs	News	# of books
Agriculture	7.8%	36.6%	21.2%	11.4%	6.5%	4.4%	4.2%	1775
Anthropology*	20.4%	28.4%	33.4%	9.7%	10.3%	7.6%	5.8%	1198
Arts*	14.9%	23.1%	23.3%	7.3%	8.4%	3.1%	2.2%	1245
Business and economics*	9.3%	33.3%	21.5%	4.9%	3.8%	6.2%	4.1%	11,987
Chemistry	1.6%	26.6%	13.8%	7.1%	4.6%	4.7%	2.8%	1651
Education	10.9%	30.3%	24.8%	7.6%	3.1%	3.8%	3.8%	4475
Engineering and technology	3.5%	26.6%	15.7%	6.8%	3.2%	2.1%	1.9%	14,140
Ethics and religion	28.3%	22.2%	20.3%	4.2%	8.5%	4.0%	3.7%	4354
Geography	9.4%	33.6%	23.8%	7.6%	5.6%	6.4%	3.3%	1367
History	27.0%	19.5%	21.1%	4.7%	12.4%	4.9%	4.1%	8798
Languages and literature	18.4%	21.4%	18.9%	4.2%	6.5%	3.5%	2.0%	9798
Law*	9.1%	34.4%	23.2%	4.7%	4.9%	10.4%	3.7%	4734
Library science and bibliography	20.2%	19.0%	25.2%	8.9%	5.3%	6.4%	0.4%	563
Mathematics	9.3%	42.7%	23.1%	6.9%	5.3%	3.3%	1.1%	4093
Medicine*	6.5%	45.6%	23.8%	10.0%	6.4%	4.7%	3.7%	12,227
Military science	16.9%	29.0%	23.7%	4.5%	6.7%	6.7%	6.0%	822
Music	25.3%	25.6%	26.2%	6.7%	15.5%	2.9%	3.4%	1161
Natural history and biology	10.5%	43.5%	27.8%	10.7%	7.4%	8.9%	3.7%	1640
Philosophy	22.5%	27.6%	23.1%	6.6%	3.4%	4.3%	1.2%	1935
Physics	7.6%	35.5%	18.8%	6.3%	5.8%	5.8%	3.0%	2628
Physiology	6.0%	43.7%	26.6%	10.9%	6.3%	5.3%	3.4%	1716
Political sciences*	12.4%	31.7%	26.3%	6.2%	6.7%	10.7%	6.5%	5098
Psychology	17.9%	33.6%	26.2%	9.0%	4.4%	7.1%	5.4%	1996
Recreation and leisure	23.5%	30.5%	30.3%	7.7%	8.9%	6.6%	5.8%	777
Social sciences	15.9%	31.1%	27.1%	6.6%	6.1%	7.1%	5.8%	9645
Zoology	15.9%	38.3%	30.3%	12.6%	15.7%	12.0%	7.2%	758
Unknown	8.7%	27.4%	18.1%	6.2%	6.8%	4.6%	3.1%	9190
*Average of 26 fields (excluding unknown)*	**14.27%**	**31.29%**	**23.83%**	**7.45%**	**6.99%**	**5.88%**	**3.77%**	**110,603**
Average of six* fields	**12.08%**	**32.74%**	**25.27%**	**7.12%**	**6.78%**	**7.12%**	**4.31%**	**36,493**

GTR: Goodreads text reviews; Mendeley: Mendeley readers; Twitter: Unique Twitter users; Facebook: Unique Facebook users; Wikipedia: Wikipedia articles; Blogs: Blog pages; News: News outlets

*Library Print and Electronic Holdings* OCLC extended results were collected by submitting ISBNs or title and author name to OCLC API (in January 2020) to extract ‘holding’ and ‘e-holding’ of books. The total holding in OCLC is the sum of these two metrics.

*Google Books Citations (GB)* Google Books offers perhaps the most promising source for identifying book citations to both journal articles (Kousha & Thelwall, 2008) and monographs (Kousha & Thelwall, 2015). However, as its results are prone to include publication advertisements and other non-relevant documents, it is important to clear the results to get true citation matches. The modification can be done automatically, for example, by the software Webometric Analyst (lexiurl.wlv.uk) in which Google Books citations can be automatically downloaded and analyzed. Here, Webometric Analyst’s preproduced protocol to harness Google Books API was used to extract Book-to-Book citations (Kousha & Thelwall, 2015) during December 2019. The least word counts of titles was set to four, in order to reduce, the possibility of false positive retrievals in the search results. The maximum word count in titles was set to six and up to three authors’ last names were included in the searches.

*Syllabus Mentions (SM)* Previous studies have used search engines such as Bing to identify mentions of journal articles (Kousha & Thelwall, 2008) as well as monographs (Kousha & Thelwall, 2016) in online syllabi and course reading lists. However, due to the limitations in Bing’s free searches, in this research the list of publications in Open Syllabus Project (opensyllabus.org) was used to identify books used for teaching. Open Syllabus Project (OSP) claims to collects list of over 1 million syllabi from about 10 to 15 years ago and mostly from English speaking countries. It also accepts syllabi of volunteer donors and copyright restricted syllabi might not be found in it. This, however, does not undermine the value of this list for studying teaching impact of resources as it might include more non-online syllabi due to receiving them directly from the universities which would, otherwise, remain unidentified with search engine-based exploration of resources. *Opensyllabus.org* was used to manually aggregate the count of university curricula referring to a book title in March and April 2020. Due to extensive manual checking required, only six fields (Dataset 2) were searched in the Open Syllabus explorer.

*Goodreads Engagements* The online social networking website of Goodreads has been introduced in previous studies as a suitable source for exploiting informal reader ratings about scholarly books (Zuccala et al., 2015). User text reviews and other Goodreads metrics are also shown to reflect various aspects of impact such as educational, cultural and research value about books and hence they can be considered as a multipurpose source of impact metrics (Kousha et al., 2017). The counts of users interacting with books in Goodreads, also known in this research as ***Goodreads Users (GU)***, star ratings or ***Goodreads Ratings (GR)***, Amazon and Goodreads textual comments or ***Goodreads Text Reviews (GTR)*** and the mean ratings given to books by users on a five-star assessment or ***Goodreads Average Rating (GAR)*** were extracted from *Goodreads.com* (October 2018) through submitting ISBNs or title and author of books via API. ISBN searches were run using the Webometric Analyst and the title and author searches were submitted via a custom software. In order to deal with numerous pages for some titles in Goodreads, total work engagements for the book rather than page statistics were used.

*Altmetric.com* Altmetric.com mainly offers mentions of research articles (Adie & Roe, 2013) on various websites and online social networking sites such as Twitter, Facebook, News Outlets, Blogs, and Wikipedia. It also offers mentions of scholarly books in the metrics it covers. For this study, all altmetric indicators were extracted from *altmetric.com* via API using ISBNs in January 2020. However, only five indicators of ***Mendeley readers****, ****Twitter unique users***, ***Facebook unique users****, ****Blog Mentions*** and ***News Stories*** are mentioned in this research, as other *altmetric.com* metrics showed only negligible counts for books. The general usability of the selected metric counts for books was also very limited (Maleki, 2020), but they are entered into the regression models in order to investigate the possible improvements in the prediction models.

*Publication Year of First Edition* Given the cumulative nature of library holding counts over time (Maleki, 2021), the first edition publication date of books were used instead of their republication dates. This mainly is because first edition year of books have a place in the cumulated events either as citations or holding and using it has been shown to normalise the effect that time has on citation accumulation across different fields (Thelwall & Fairclough, 2015). Publication years are grouped in six consecutive time periods including prior to 2003, 2003–2005, 2006–2008, 2009–2011, 2012–2014 and 2015–2017 in order to include enough books in each publication range to reach statistically meaningful calculations from 40 (2003–2005) for Arts to 3499 (2012–2014) for Business and Economics.

### Analysis

*Correlation Analysis* Spearman’s correlation results between the three library holding-based metrics (LPH, LEH, and TLH) and other indicators were analysed. Two datasets of books with zero metric counts and without zero counts were examined in a parallel way to produce more robust results for future comparisons. Due to dealing with citation data which often have skewed distribution, Spearman’s Correlation coefficients are used.

*Prediction Analysis* The regression models in present study were designed in two ways (a) to determine the relative ability of library print, electronic and total holdings in predicting citation and altmetric indicators; and (b) to explore the role of metrics in multivariate book assessment models for explaining variances for library print, electronic, and total holdings by other book indicators. Thus, the prediction tasks were supposed to show the extent to which and how library holding metrics, print or electronic, could be explained by other metrics, either combined or alone.

For prediction analysis, the Ordinary Least Squares Regression model was constructed on log-normalized counts of metrics (c) as proposed by Thelwall (2017). Log-transformed data create normalized distributions suitable for linear regression and produce more realistic results for metrics with many zero metric counts (Thelwall, 2017), which is apparent in current research for majority of indicators (see Table 1). By using this method all books either with zero or non-zero metric counts were included in the models in order to reach a more universal understanding of impact. In order to perform log-transformation, first all books with or without respective metric counts were added one unit (c + 1), and then natural logarithm was performed on them. To test all metrics equally for all 26 fields, *Enter* model is used which receives all independent variables at once rather than stepwise. Despite using all metrics in models, other combinations of metrics were also examined to aid discussions about the libcitation’s capabilities and differences as tools for impact assessment. Subject-level findings on coefficients and R^2^ scores for all the eleven metrics are in the Online Appendix.^2^ In order to meet the pre-conditions of linear regression, in addition to log-transformation, multicollinearity, condition index and tolerance of metrics are also controlled. In sum, because of strong correlation level between Goodreads Rating and Goodreads Users (*r* = 0.833, *p* < 0.001), Goodreads Ratings metric is removed from the regression analyses to prevent collinearity. All other variables met the collinearity requirements (condition index < 8) and were tolerable (VIF < 5) in the regression models.

## Findings

### Correlations across fields and over time: comparing library print, electronic and total holdings

In response to the first research question, Table 4 indicates the average of significant correlation coefficients across 26 fields. Six Spearman correlation analyses were performed for each field between library holding metrics (LPH, LEH and TLH) and one other metric across its two datasets, (a) zeros included and (b) zeros excluded. The average correlation coefficients between metrics and LPH with zero counts of metrics (−0.009 < *r* < 0.534) and without zero counts (−0.140 < *r* < 0.533) were significant weak or moderate and placed at higher ranges than TLH (−0.102 < *r* < 0.347 and −0.167 < *r* < 0.366, respectively) and substantially above LEH (−0.050 < *r* < 0.269 and −0.149 < *r* < 0.214, respectively). This suggests that TLH, which is the sum of LPH and LEH, depicts correlation coefficients in the middle range below LPH and above LEH. However, it needs to be noted that the average correlation coefficients across fields are used here to only indicate the general differences between LH metrics and other indicators. Thus, these coefficient values should not be used or need to be used cautiously as they are not the actual relationship coefficients as in field-level and the significance of the correlation levels cannot be specified to the average values. The correlation coefficients at subject level along with *significance* of coefficients need to be found and referenced if necessary from Figshare Online Appendix Tables 1–6.

**Table 4:** Average of significant spearman’s correlation coefficients across 26 fields (Dataset 1) between library holdings (Print, E-book and total) in two datasets of books (with zero metric counts and without zero metric counts) and other research metrics

Metrics	Print holding (LPH)		E-book holding (LEH)		Total holdings (TLH)	
	Non-zeros	With zeros	Non-zeros	With zeros	Non-zeros	With zeros
Scopus citations	**0.443**	**0.481**	0.167	0.234	0.292	0.347
Google books citations	**0.385**	**0.431**	0.025	0.075	0.187	0.232
Syllabus mentions	**0.349**	**0.534**	0.062	0.169	0.128	0.345
Goodreads users	**0.533**	**0.385**	0.214	0.269	0.366	0.342
Goodreads ratings	**0.482**	**0.392**	0.199	0.244	0.343	0.331
Goodreads text reviews	**0.358**	**0.289**	0.151	0.173	0.267	0.245
Goodreads average rating	** − 0.115**	**0.312**	− 0.075	0.211	− 0.093	0.272
Mendeley readers	0.263	− 0.009	0.183	**0.032**	**0.264**	0.006
Twitter users	− 0.140	** − 0.105**	− 0.149	− 0.001	** − 0.167**	− 0.077
Facebook walls	** − 0.048**	** − 0.112**	− 0.007	− 0.050	− 0.013	− 0.102
Wikipedia articles	0.131	0.069	**0.140**	0.075	0.118	**0.083**
Blog pages	0.221	**0.072**	0.192	0.045	**0.223**	0.061
News posts	**0.318**	**0.105**	− 0.128	0.067	0.149	0.072

Syllabus mentions is the exception where average is calculated for six fields in Dataset 2

Bold coefficients represent the strongest average

*Correlation Coefficients for Zero vs. Non-zero Metric Counts* Including books with zero metrics counts have generally *increased* the average correlation coefficients across fields for all metrics, except for Goodreads-related and online metrics. This increase was seen in the average correlation coefficients of the three holding metrics, however, more noticeably in LPH when comparing the dataset with non-zero to zero-included dataset for Scopus Citations (0.443 and 0.481, respectively), GB Citations (0.385 and 0.431), and Syllabus Mentions (0.349 and 0.534), suggesting that books without respective events can also have relatively lower library print holdings, probably due to prominence of academic library members in WorldCat. However, removing books with zero Goodreads Users *increased* the median correlations with LPH (0.385–0.533) and TLH (0.342–0.366) but *decreased* them with LEH (0.269–0.214). This suggests that print holdings of books without Goodreads Users can still be rather high, while books with more library *eholdings* are enjoying slightly better Goodreads engagement. This pattern is recurrent for Goodreads Ratings and Text Reviews, but not Average Ratings (Table 4).

*Impact of Time on Correlations* Because books also need time to accrue citations, it is important to check the changes in correlation levels over years. In order to cover the answer to the fifth research question, Table 5 shows average significant correlation coefficients between LPH and LEH and other metrics for six fields, over six time periods. Like the overall correlation coefficients, the strength of correlations between LPH and metrics is greater than that of LEH across all years and disciplines, despite occasional inconsistencies in years prior to 2008 in three areas of *Anthropology*, *Arts* and *Economics and Business* due to lower frequency of books (Online Appendix Tables 7 and 8).

**Table 5:** Average spearman’s correlation coefficients between library holdings and other metrics across six fields and over time (Dataset 2)

Subject	< 2003	2003–2005	2006–2008	2009–2011	2012–2014	2015–2017
Scopus citations	0.279	0.338	0.334	**0.494**	0.459	0.374
	**0.262**	0.166	0.144	0.158	0.187	0.150
Google books citations	0.350	**0.418**	0.354	0.336	0.315	0.295
	0.044	0.034	− 0.075		**0.100**	0.082
Syllabus mentions	0.474	**0.523**	0.433	0.473	0.406	0.191
	**0.178**	0.161	− 0.092	0.106	0.142	0.093
Goodreads users	0.299	0.410	0.393	**0.448**	0.419	0.313
	**0.272**	0.220	0.231	0.221	0.192	0.216
Goodreads ratings	0.329	0.394	0.375	**0.465**	0.453	0.356
	**0.306**	0.255	0.196	0.214	0.200	0.224
Goodreads text reviews	0.301	0.312	0.313	0.330	**0.336**	0.259
	0.184	**0.226**	0.147	0.156	0.162	0.159
Goodreads average ratings	0.250	**0.346**	0.302	0.338	**0.346**	0.285
	**0.265**	0.130	0.207	0.172	0.174	0.203
Mendeley readers	0.145		**0.272**	0.155	0.087	− 0.147
	0.198	**0.272**	− 0.010	− 0.060	− 0.026	0.158
Twitter users	0.164	0.161	**0.213**	0.188	0.188	0.067
	**0.166**	− 0.101	0.110	0.004	0.063	0.079
Facebook walls	**0.116**	− 0.116	− 0.079	0.083	0.078	0.079
	**0.142**	0.091	0.085	0.081	0.002	− 0.002
Wikipedia articles	0.103	**0.152**		0.055	0.093	0.052
	0.097	**0.099**	− 0.032	0.043	0.058	0.097
Blog pages	0.185	0.164	0.156	0.195	**0.208**	0.186
	0.103	**0.118**	− 0.009	0.082	0.107	0.071
News posts	**0.179**	0.139	0.117	0.112	0.144	0.143
	0.070	**0.139**	0.086	0.081	0.082	0.064

The above line in each cell indicates correlation coefficients with Library Print Holdings and the line below indicates that of Library Electronic Holdings

Bold coefficients represent the strongest year for each field

*Correlations Trend for LPH* Table 5 also suggests that the correlations between LPH and other metrics are significantly moderate or weak and despite a general downward trend, often have undulated over time. Overall, the patterns for different metrics tend to break in 2009–2011 when correlations are reinforced before falling again. The strongest average correlation coefficients across fields were frequently observed in 2003–2005 for the relationship between LPH and Syllabus Mentions at 0.523, GB Citations at 0.418, Goodreads Average Ratings at 0.346, as well as Wikipedia Articles at 0.152. Most of the coefficients between LPH and the altmetric indicators showed a weak peak below 0.3 at different time periods. However, for three indicators of Scopus Citation (0.494), Goodreads Users (0.448), and Goodreads Reviews (0.465), the strongest average correlation coefficients across fields are experienced in 2009–2011. This is mainly because library holding counts’ pattern was broken from 2010 onwards (Maleki, 2021), presumably reflecting the considerable rise in counts of print book holdings contemporary with the same rise in electronic holdings. At disciplinary level, the highest correlation coefficients observed in this research were in 2003–2005 between LPH and GB Citations in Arts at 0.664 (*n* = 33) and LPH and Syllabus Mentions in Political Science at 0.601 (*n* = 203), both significant at *p* < 0.001 (see Online Appendix Table 7).

*Correlations Trend for LEH* The correlations between LEH and most of the metrics similarly showed a downward trend, but did not significantly surpass 0.3, with the exception of LEH and Goodreads Rating (*r* = 0.42) in Arts 2003–2005 which is not essential, due to lower book frequency (*n* = 40). Correlations between LEH and Google Books Citations and Syllabus Mentions were even significant *negative* in some years in *Law* and *Medicine* (see Online Appendix Table 8), suggesting that electronic versions of older books with scholarly and educational usage are far less acquired compared to more recent ebooks.

### Comparing the predictive power of library print, electronic, and total holdings

In response to the second research question, the relative statistical power of print, electronic and total library holding counts in predicting other metrics was examined. Table 6 shows the average adjusted R square scores of four models across 26 fields: first model represents print holding along with eholding counts as two predictors of the model and the three remaining models represent one variable (Library Total Holdings, Library Print Holdings, or Library Eholdings) as the sole predictors of each metric. This is examined for all fields and the averages are reported for both Datasets 1 and 2.

**Table 6:** Average R^2^ scores of linear regression models across 26 fields (left) and six sampled fields (right)

Metrics/models	Mean adjusted R^2^ in 26 fields (Independant variables)				Mean adjusted R^2^ in 6 sampled fields (Independant variables)			
	LPH and LEH	Only TLH	only LPH	Only LEH	LPH and LEH	Only TLH	only LPH	Only LEH
Scopus citations	**24.4%**	12.8%	**21.9%**	5.1%	**25.4%**	**14.3%**	**23.9%**	5.3%
Google books citations	19.6%	6.6%	19.3%	1.1%	21.5%	7.6%	21.5%	1%
Syllabus mentions	–	–	–	–	23.8%	10.3%	23.7%	2.1%
Goodreads users	21.3%	**13%**	17.7%	**6.5%**	20.9%	12.9%	18.9%	**5.4%**
Goodreads ratings	19.1%	11.5%	16.8%	4.9%	18.8%	11.6%	17.4%	4.3%
Goodreads text reviews	10.0%	6%	8.9%	2.4%	10.0%	6.1%	9.4%	2%
Goodreads average rating	13.5%	8.5%	10.7%	4.8%	13.0%	8.8%	11.1%	4.3%
Mendeley readers	3.1%	2.0%	0.8%	2.8%	1.7%	1.5%	0.6%	2.1%
Twitter users	1.7%	1.4%	0.9%	1.3%	0.7%	0.9%	0.2%	0.6%
Facebook walls	1.3%	0.9%	0.9%	0.7%	1.0%	0.7%	0.7%	0.5%
Wikipedia articles	0.8%	0.6%	0.5%	0.6%	0.6%	0.4%	0.4%	0.3%
Blog pages	1.4%	0.4%	0.6%	0.3%	1.8%	0.5%	0.8%	0.4%
News posts	0.7%	0.8%	1.3%	0.6%	1.0%	0.8%	1.5%	0.5%

There are two independent variables only in the first model (LPH and LEH) and there is only one independent variable in the other three models

Bold percentages represent the metric with strongest prediction ratio in the regression model

*Comparing LPH Only models with LPH along with LEH* Table 6 gives the average R^2^ scores of various metrics across 26 fields. It indicates that when LPH and LEH together were entered as the combined predictors of the models (24.5%), the average R^2^ scores of Scopus Citations were slightly stronger than that of LPH as the sole predictor of models (21.9%), however substantially stronger than those of only TLH (12.8%) and only LEH predictor models (5.1%). This suggests that despite very weak prediction power of eholdings compared to print holdings, there still is an underlying ability in eholdings to slightly improve the average of prediction scores, when accompanying LPH, particularly for Goodreads Users (17.7% in only LPH vs. 21.3% in LPH and LEH), but very minimally for GB Citations (19.3% vs. 19.6%). The results in field level are offered in the Online Appendix Tables 9–11.

*Is LPH Always Stronger than LEH in Predicting Metrics?* In response to the third research question, Table 7 gives the average standardized coefficients across fields for the combined LPH- and LEH-predictor models. Since the purpose of prediction task in this research was only to speculate underlying differences between print holdings and eholdings rather than giving a prediction formula, the standardized *β* is reported as the regression coefficient instead of unstandardized B. Thus, for instance, the strongest average regression coefficient across fields in Table 7, which relates to Syllabus Mentions (0.480), suggests that by one standard deviation increase in Syllabus Mentions, on average LPH increases by 0.480 times of its own standard deviation across fields.

**Table 7:** Average standardized coefficients (*β*) in the linear regression model with two independent variables (LPH and LEH) across 26 fields (left) and six sampled fields (right)

Metrics	Mean regression coefficient (Beta)			
	For 26 fields		For 6 sampled fields	
	LPH	LEH	LPH	LEH
Scopus citations	**0.449**	0.149	**0.461**	0.123
Google books citations	**0.441**	− 0.031	**0.467**	− 0.054
Syllabus mentions	**–**	–	**0.480**	0.036
Goodreads users	**0.391**	0.183	**0.403**	0.136
Goodreads ratings	**0.385**	0.154	**0.391**	0.131
Goodreads text reviews	**0.279**	0.100	**0.290**	0.073
Goodreads average rating	**0.297**	0.165	**0.302**	0.128
Mendeley readers	0.046	**0.075**	0.018	**0.064**
Twitter users	** − 0.073**	0.017	** − 0.028**	− 0.020
Facebook walls	** − 0.113**	− 0.007	** − 0.077**	− 0.015
Wikipedia articles	**0.066**	0.061	**0.053**	0.048
Blog pages	**0.110**	0.046	**0.125**	0.001
News posts	**0.070**	0.039	**0.087**	0.056

Bold coefficients represent the library holding metric with stronger prediction rate for the metric

Table 7 suggests that metrics’ variance in all models are best explained by LPH, except for *Mendeley Readers*. The field level results for Mendeley show that in 18 (70%) out of 26 fields, LEH had stronger coefficients. At disciplinary level, library ebook holdings showed a significant advantage for a considerable number of fields in predicting Twitter (16 of 23 fields), Facebook (11 of 22 fields), and Wikipedia (11 of 21 fields) uptake of books, as well. The extended prediction results of each metric-field for Table 7 are given in the online appendix Tables 12–25.

**Fig. 2:**
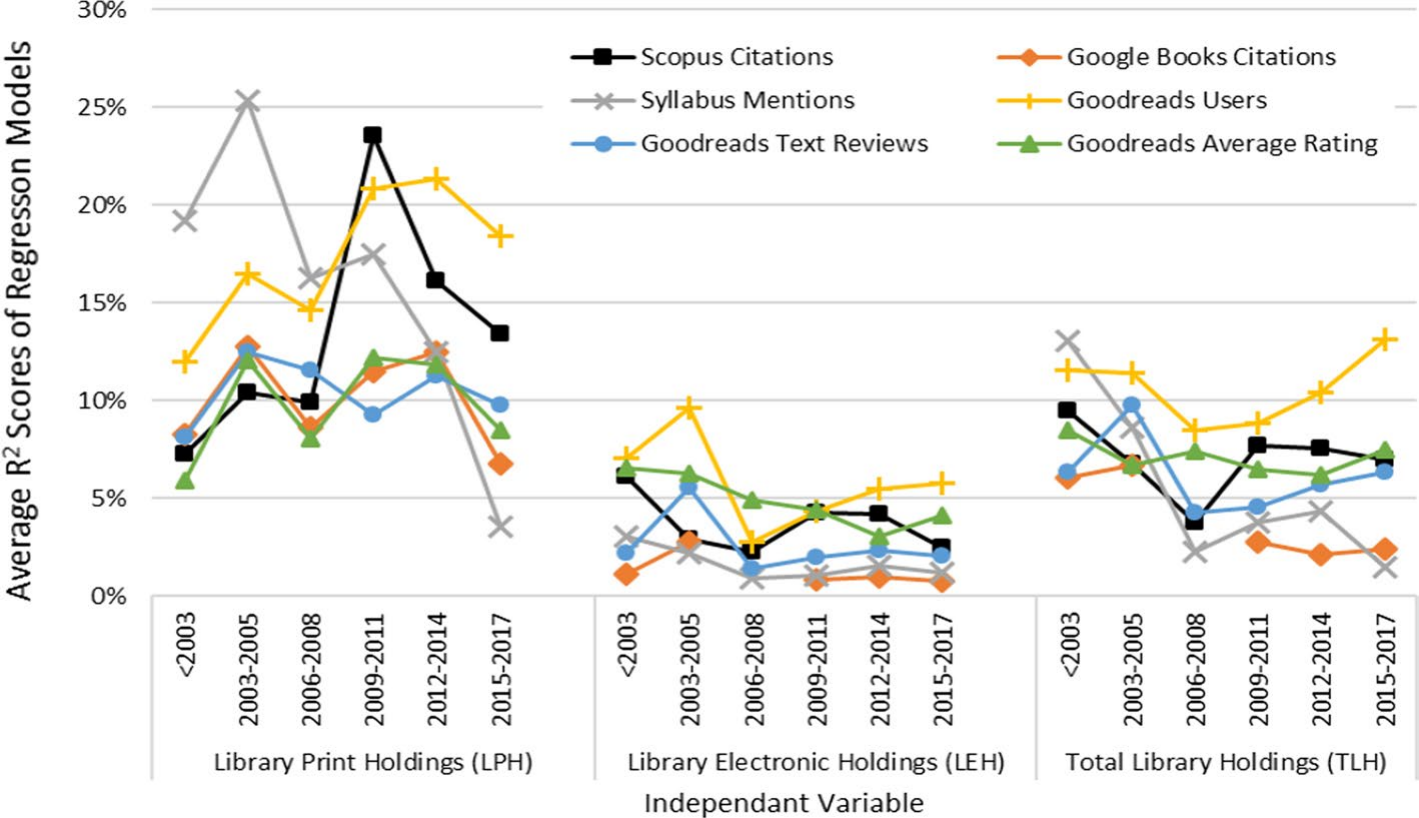
Average R^2^ scores of regression models with LPH, LEH or TLH as independent variable and one other metrics as the predicted variable across six sampled fields over time

*Robustness of Prediction Models over Time* Because of a friction in the pattern of library holding counts for books published from 2010 onwards (Maleki, 2021), the robustness of results produced in the above models is examined by repeating the regressions across the periods of years for six sample fields, illustrated for their averages in Fig. 2 (see field-based results in the online appendix Figs. 1–6). Results show that majority of the average R^2^ scores over years by LEH as the predictor were often below 5%; by TLH were between 5 and 10%; and by LPH were between 10 and 20%. This suggests that for various metrics the best predictions over years are made by LPH, despite broad variations and instability over time.

### Dimensions of library total, print and electronic holdings across disciplines

In order to investigate the underlying differences between LPH, LEH and TLH in terms of the impact they indicate, statistical prominence of aspects of impact in co-presence of different metrics is examined. In response to the fourth research question, we statistically explain various dimensions of LPH, LEH or TLH through regression models using various sets of all other metrics, this time as the independent variables. Table 8 shows average adjusted R^2^ scores of models that predict each of LPH, LEH, and TLH, by single or various combined sets of metrics across 26 fields (Syllabus mentions excluded) and six sampled fields where Syllabus Mentions metric is included in the models.

**Table 8:** Average Adjusted R^2^s of fields for various combinations of metrics. LPH, LEH and TLH are separately placed as the dependent variable

Model variables	Mean adjusted R^2^ (26 fields) (excluding syllabus mentions)			Mean adjusted R^2^ (6 fields) (including syllabus mentions)		
	LPH	LEH	TLH	LPH	LEH	TLH
Scopus	***21.9%***	5.1%	12.8%	***23.9%***	5.3%	14.3%
GB	***19.3%***	1.1%	6.6%	***21.5%***	1.0%	7.6%
SM				***23.7%***	2.1%	10.3%
GU	***17.7%***	6.5%	13.0%	***18.9%***	5.4%	12.9%
GR	***16.8%***	4.9%	11.5%	***17.4%***	4.3%	11.6%
GTR	***8.9%***	2.4%	6.0%	***9.4%***	2.0%	6.1%
GAR	***10.7%***	4.8%	8.5%	***11.1%***	4.3%	8.8%
Mendeley	0.8%	***2.8%***	2.0%	0.6%	***2.1%***	1.5%
Twitter	0.9%	1.3%	***1.4%***	0.2%	0.6%	***0.9%***
Facebook	0.9%	0.7%	***0.9%***	0.7%	0.5%	***0.7%***
Wiki	0.5%	0.6%	***0.6%***	0.4%	0.3%	***0.4%***
News	***0.6%***	0.3%	0.4%	***0.8%***	0.4%	0.5%
Blog	***1.3%***	0.6%	0.8%	***1.5%***	0.5%	0.8%
Scopus*GB	***29.2%***	5.6%	14.3%	***31.9%***	5.5%	16.0%
Scopus*GU	***29.8%***	8.8%	19.3%	***31.5%***	7.9%	19.9%
Scopus*GR	***29.7%***	7.7%	18.5%	***31.0%***	7.2%	19.3%
Scopus*GTR	***26.6%***	6.4%	16.1%	***28.3%***	6.2%	17.1%
Scopus*GAR	***26.1%***	7.8%	16.7%	***27.8%***	7.4%	18.0%
GB*GU	***31.8%***	7.0%	16.9%	***34.5%***	5.8%	17.7%
GB*GR	***31.2%***	5.4%	15.6%	***33.4%***	4.8%	16.6%
GB*GTR	***26.0%***	3.0%	11.2%	***28.3%***	2.8%	12.5%
GB*GAR	***26.7%***	5.4%	13.2%	***29.0%***	4.8%	14.6%
Scopus*GB*GU	***36.6%***	9.4%	20.7%	***39.3%***	8.0%	21.5%
Scopus*GB*GU*GTR*GAR	***36.8%***	9.6%	20.8%	***39.5%***	8.3%	21.8%
Mendeley*Twitter*Facebook*News* Blog*Wiki	***3.8%***	3.5%	3.4%	***3.3%***	2.2%	3.1%
Scopus*GB*GU*GTR*GAR*Mendeley *Wiki*Twitter*Facebook*News*Blog	***38.5%***	11.7%	22.5%	***40.5%***	9.9%	23.5%
SM*Scopus				***33.1%***	5.6%	17.2%
SM*GB				***33.8%***	2.5%	13.4%
SM*GU				***32.0%***	6.0%	17.5%
SM*GR				***30.4%***	4.9%	16.2%
SM*GTR				***27.6%***	3.3%	13.4%
SM*GAR				***28.6%***	5.3%	15.5%
SM*Scopus*GB				***38.4%***	5.8%	18.1%
SM*Scopus*GU				***37.9%***	8.0%	21.4%
SM*GB*GU				***41.0%***	6.2%	20.1%
SM*Scopus*GB*GU				***43.3%***	8.2%	22.5%
Scopus*GB*SM*GU*GTR*GAR				***43.5%***	8.4%	22.9%
Scopus*GB*SM*GU*GTR*GAR*Mendeley *Wiki*Twitter*Facebook*News*Blog				***44.7%***	10.0%	24.6%

Scopus: Scopus citations; GB: Google books citations; SM: Syllabus mentions; GU: Goodreads users; GR: Goodreads ratings; GTR: Goodreads text reviews; GAR: Goodreads average ratings; Empty cells are not tested because syllabus mentions was not available. Facebook: Facebook users; Wiki: Wikipedia articles; Twitter: Twitter users; Mendeley: Mendeley readers; News: News outlets; Blogs; Blog pages. ***Bold-Italic*** R^2^ scores indicate the strongest metric or set of metrics in prediction of relevant library holding statistics in each dataset

In terms of prediction rate, in all models, LPH has significantly better prediction rate than TLH and LEH, except for two single-independent-variable models of Mendeley and Twitter where LEH had very small but still larger R^2^ scores (2.8% and 1.3%, respectively) than LPH (0.8% and 0.9%, respectively). This is in line with the correlation results. In presence of all 12 metrics, average R^2^ scores show that LPH (44.7%) is 1.8 times better explained than TLH (24.6%), whilst LEH (10%) is as 0.4 times TLH prediction rate (Table 8).

*Single-independent-variable Models* On average the best prediction rate by one independent variable was made with Scopus Citations (21.9%) for LPH, whereas the highest for TLH and LEH was seen with Goodreads Users (6.5% and 13%, respectively), which is a persistent pattern between the 26- and six-field datasets.

*Two-independent-variable Models* The highest average R^2^ score for models with two independent variables in 26-field dataset, are for LPH predicted by GB*GU (31.8%); the best models for TLH and LEH consisted of Scopus*GU (19.3% and 8.8%, respectively). The same pattern persists in six-field dataset, suggesting the key role that GB Citations play in LPH prediction models and Scopus Citations in LEH and TLH prediction models.

*Three-independent-variable Models* The contribution of Syllabus Mentions (SM*GB*GU) to three independent variable models is slightly more substantial (41%) than Scopus Citations (Scopus*GB*GU) (39.3%) for LPH, whereas the reverse is true for TLH (20.1% and 21.5%, respectively) and LEH (6.2% and 8%, respectively) with higher contribution of Scopus Citations.

*More than Three Independent Variable Models* By increasing the number of variables in the model the adjusted R^2^ scores improved with one of the best average scores reached by the four major indicators of Scopus*GB*SM*GU which explained 43.3% of the variance for LPH, 22.5% of the variance for TLH and 8.2% of the variance for LEH. The contributions of GTR and GAR to the models were very minimal (at best 0.4% for TLH). Very minimal improvements (max. of 1.2% for LPH) in the models occurred when six *Altmetric* indicators, namely Mendeley readers, Wikipedia Citations, Twitter Unique Users, Facebook Unique Users, News Posts, and Blog Mentions, were included (Scopus*GB*SM*GU*GTR*GAR*Mendeley*Wiki*Twitter* Face*News*Blog) reaching to 44.7% for LPH, 10% for LEH and 24.6% for TLH.

*Prominent Dimensions in Multivariate Predictions* Tables 9, 10, 11 show the standardized coefficients (*β*) of the 12 independent variables in the last model for predicting TLH (Table 9), LPH (Table 10), and LEH (Table 11) in six sample fields. The major indicators explaining the variances for TLH are Scopus Citations (in 4 out of six fields), and Goodreads Users (2 fields); for LPH are Google Books Citations (4 fields), Scopus Citations (1 field) and Syllabus Mentions (1 field), and for LEH are Goodreads Users (3 fields), Scopus Citations (2 fields) and Mendeley Readers (1 field). Mendeley, Twitter, and Facebook had negative coefficients in LPH prediction models, whilst this is also true for LEH, the coefficients of Mendeley for LEH were more likely to be positive, particularly in Medicine (Table 11). The reverse was true for GB Citations whose coefficients in LPH models were always positive and in LEH insignificant or weak negative.

**Table 9:** Statistically significant linear regression standardized coefficients (*β*) in six sample fields for TLH

Fields	Scopus	GB	SM	GU	GTR	GAR	Mendeley	Twitter	Facebook	Wiki	Blogs	News	R^2^
Anthropology	**0.176**	0.149	0.104^**^	0.172^**^			− 0.095^**^	− 0.136					22.6%
Arts	**0.221**	0.110	0.068^*^	0.117^*^		0.178	− 0.101		− 0.074^**^	0.051^*^			28.2%
Economics	0.182	0.102	0.152	**0.223**			0.021^*^	− 0.048					23.1%
Law	0.179	0.133	0.107	**0.247**				− 0.121		.029^*^	− 0.044^**^	0.039^**^	26.7%
Medicine	**0.181**	0.046	0.125	0.157			0.121	− 0.040		0.019^*^	0.034^**^		18%
Political science	**0.214**	0.122	0.164	0.179		0.055^**^	− 0.083	− 0.089			− 0.037	0.027^*^	28.9%

The dependent metric is total library holding. Only Goodreads rating is excluded from the models due to causing collinearity

Bold coefficients represent the metric with the strongest prediction rate in the field

All R^2^s are significant at *p* < 0.001; All coefficients are significant at *p* < 0.001 except *p < 0.05; **p < 0.01

**Table 10:** Statistically significant linear regression standardized coefficients (*β*) in six sample fields for LPH

Fields	Scopus	GB	SM	GU	GTR	GAR	Mendeley	Twitter	Facebook	Wiki	Blogs	News	R^2^	R^2^ LPH/TLH
Anthropology	0.151	0.236	**0.271**	0.188	0.081^*^		− 0.097						40.8%	1.81
Arts	0.160	**0.270**	0.248	0.162	0.069^*^		− 0.150						42.4%	1.50
Economics	0.209	**0.305**	0.224	0.177			− 0.122	− 0.038	− 0.028		0.042		44.9%	1.94
Law	0.209	**0.290**	0.174	0.267			− 0.060	− 0.031^**^			0.032^**^		46.1%	1.73
Medicine	0.216	0.219	**0.277**	0.240	− 0.200^*^	0.027^**^	− 0.065	− 0.04		0.020	0.027^**^	0.020^*^	47.1%	2.62
Political science	**0.246**	**0.246**	0.231	0.201			− 0.104	− 0.041^**^				0.043	46.7%	1.62

The dependent metric is library print holding. Only Goodreads rating is excluded from the models due to causing collinearity

Bold coefficients represent the metric with the strongest prediction rate in the field

All R^2^s are significant at *p* < 0.001; All coefficients are significant at *p* < 0.001 except *p < 0.05; **p < 0.01

**Table 11:** Statistically significant linear regression standardized coefficients (β) in six sample fields for LEH

Fields	Scopus	GB	SM	GU	GTR	GAR	Mendeley	Twitter	Facebook	Wiki	Blogs	News	R^2^	R^2^ LEH/TLH
Anthropology	0.128			0.165^**^			− 0.076^*^	− 0.088^**^					7.3%	0.32
Arts	**0.204**					0.137^**^			− 0.067^*^				10.2%	0.36
Economics	0.133	− 0.080	0.040	**0.229**	− 0.032^*^		0.081				− 0.036^**^		9.4%	0.41
Law	0.133		0.039^*^	**0.170**			0.057	** − 0.177**			− 0.073	0.041^**^	11.5%	0.43
Medicine	0.172	− 0.055	− 0.035	0.069			**0.186**				0.024^*^		8.9%	0.49
Political science	**0.196**		0.063	0.130		0.069^**^	− 0.065	− 0.066			− 0.057		12.8%	0.44

The dependent metric is library electronic holding. Only Goodreads rating is excluded from the models due to causing collinearity

Bold coefficients represent the metric with the strongest prediction rate in the field

All R^2^s are significant at *p* < 0.001; All coefficients are significant at *p* < 0.001 except *p < 0.05; **p < 0.01

**Fig. 3:**
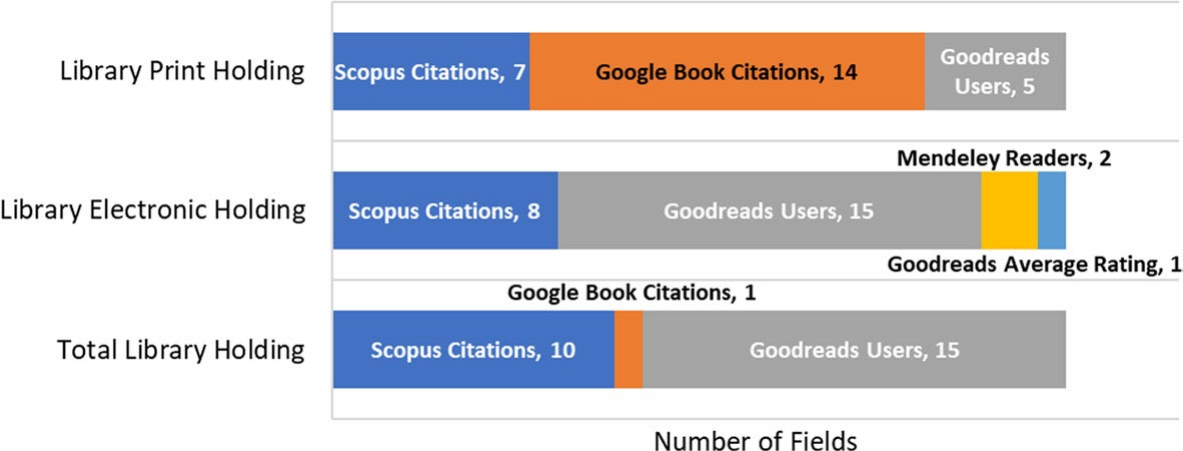
Number of fields with the highest significant standardized coefficient for the metric among 11 variables in the regression model (Scopus*GB*GU*GTR*GAR*Mendeley*Wiki*Twitter*Facebook*News*Blog) with LPH, LEH and TLH as the independent/predicted variable

The significant standardized regression coefficients (*β*) of the models with 11 independent variables (excluding Syllabus Mentions) are given for 26 fields in the Tables 26–28 in Online Appendix. Figure 3 summarizes the results for these syllabus mention-excluded models, in terms of the number of fields which had the strongest standardized regression coefficient for the respective metric. It was found that inclusion of Syllabus Mentions was helpful and important in the LPH prediction models of all fields (particularly in Medicine) (Table 10), otherwise, in absence of Syllabus Mentions, GB Citations (14 out of 26 fields), Scopus Citations (7 fields), or Goodreads Users (5 fields) would be the strongest prediction factors (Fig. 3). Majority of the fields in prediction of LEH and TLH had the strongest Goodreads User coefficients (both 15 fields), confirming the above results.

## Discussion

One limitation encountered in the analysis was that Goodreads Rating and Goodreads Users were highly correlated and it was not possible to include them simultaneously in all regression analysis. Therefore, Goodreads Rating were excluded from all models as their inclusion would have interfered with Goodreads Users with negative coefficient and caused collinearity in the regression models, whilst it would not have improved the predictive ability of the models nor incorporated minor improvements.

In answer to the first research question, the relationship between “Total Holdings” and other metrics in previous studies (Table 1), whilst considering the variations in dataset coverages and sizes, was generally close to the correlations reported in this research, perhaps except for Syllabus Mentions and Wikipedia Article Citations. The correlation between library holdings and Syllabus mentions in Kousha and Thelwall (2015) was slightly weaker (0.121) than current research (0.345), possibly due to the larger size of dataset in current study. However, in contrast, for Wikipedia Article Citations all correlations observed in current research were weaker than the best observation (*r* = 0.387 in 2010 Arts books) in Kousha and Thelwall (2017), possibly because of the differences in methods of data collection.

The average correlations with print holdings, despite much stronger than total holdings in this research, were slightly stronger than that of total holdings in previous studies. For Scopus Citations, all the correlation coefficients with Libcitations in previous studies were very weak (max *r* = 0.208 in Zuccala et al., 2015), whereas current research found higher average correlations at medium level for LPH both in zero (*r* = 0.481) and non-zero datasets (*r* = 0.443). For Google Books Citations, however, the average correlation in non-zeros datasets (*r* = 0.385) was only slightly stronger than the highest report in previous studies (*r* = 0.326 in Kousha & Thelwall, 2015), suggesting that print holdings have often produced weak correlation results with GB citations, despite some medium level correlations (*r* > 0.4) at subject level (12 out of 26 field, Table 2 in online appendix). For Goodreads Reviews, the average correlation for LPH at zeros dataset (*r* = 0.385) was slightly above some previous studies, such as *r* = 0.346 in Kousha and Thelwall (2017), and at non-zero dataset, current research showed stronger coefficients (*r* = 0.533) than Zuccala et al. (2015) (*r* = 0.446) (further compared in Table 12). This suggests that inclusion or exclusion of books with zero events in different metrics is important in the correlation analysis, since strength of correlations can substantially change. In sum, correlation coefficients observed in this research for LPH might be slightly bigger than in previous reports, but previous studies have tested various fields, datasets and years, making comparisons difficult. Thus, the importance of this research lies in the broad range of subjects, years and fields studied and simultaneous examination of total, print and electronic holdings that made it possible to indicate differences.

**Table 12:** A case comparison of correlation coefficients for books in the field of history between current research and Zuccala et al. (2015)

Research	Data properties: subject field = history	Correlation test variables		
		LH-scopus citations	LH-goodreads reviews/users	LH-goodreads ratings
Zuccala et al. (2015)	n = 997 (Scopus citations (≥ 4) and goodreads ratings (≥ 10) above the 75th percentile) Books in references of journals articles published in 2007–2011	ARL holdings *r* = 0.208 Non-ARL holdings *r* = 0.072	ARL holdings *r* = 0.072 Non-ARL holdings *r* = 0.448	ARL holdings *r* = 0.101 Non-ARL holdings *r* = 0.467
Current research	*n* = 448 Scopus citations (≥ 16) and goodreads ratings (≥ 11) above the 75th percentile Scopus pub. years ≤ **2017**	TLH *r* = 0.242 LPH *r* = 0.373 LEH r = −	TLH *r* = 0.152 LPH *r* = 0.417 LEH *r* = − 0.096*	TLH *r* = 0.214* LPH *r* = 0.495 LEH *r* = −
	n = 162 Scopus citations (≥ 26) and goodreads ratings (≥ 13) above the 75th percentile Scopus pub. years ≤ **2011**	TLH *r* = 0.366 LPH *r* = 0.364 LEH *r* = 0.218	TLH *r* = 0.247 LPH *r* = 0.478 LEH *r* = −	TLH *r* = 0.245 LPH *r* = 0.516 LEH *r* = −
	n = 8798 dataset **with zero** counts All years	TLH *r* = 0.370 LPH *r* = 0.485 LEH *r* = 0.253	TLH *r* = 0.371 LPH *r* = 0.426 LEH *r* = 0.276	TLH *r* = 0.380 LPH *r* = 0.434 LEH *r* = 0.275
	n = 4681 dataset **without zero** counts All years	TLH *r* = 0.286 LPH *r* = 0.460 LEH *r* = 0.149	TLH *r* = 0.395 LPH *r* = 0.550 LEH *r* = 0.238	TLH *r* = 0.337 LPH *r* = 0.488 LEH *r* = 0.171

All correlation coefficients are significant at *p* < 0.001; except *p < 0.05

In order to compare findings with previous studies, Table 12 offers a case comparison of correlation results for the field of *History*. Zuccala et al. (2015) had reported the strongest correlation coefficients for library holdings on 997 popular and academic History books cited in journal articles in 2007–2011 with Scopus Citations (≥ 4) and Goodreads Ratings (≥ 10) above the 75th percentile. All of the correlation coefficients in current research between TLH and Scopus Citation, Goodreads Rating or Goodreads User/Reviews were significant weak and below the observation in Zuccala et al. (2015) for Non-ARL Holdings (i.e. Non-American, Research Library Holdings) either in the top quartile or total dataset (Table 12). In fact, as the correlation coefficients at the top quartile of citations for current research were lower than total dataset, there is a possibility that the same has happened for the data in Zuccala et al. (2015) for both ARL and non-ARL libraries. The reason for the medium-level correlations at non-ARL libraries, but weak correlation level at ARL libraries in Zuccala et al. (2015) is not apparent, but a possible explanation can be more upload of *print collections* from non-ARL libraries into WorldCat rather than electronic ones which is more common in ARL or American Research Libraries. This inference needs a distinct investigation, perhaps like Zuccala et al. (2021) and Zhou and Zhang’s study (2020b), as they give some evidence to suggest that there is a need to make distinction between the libraries in the US and other parts of the world or at least take into account that geographical factors might also have a place in the distribution of print and electronic holdings in WorldCat. Interestingly, in current research, all correlation coefficients between LPH and Goodreads metrics for History books were at the medium level and, particularly in the dataset without zero counts were above non-ARL Holdings’ results, both for Goodreads Ratings (*r* = 0.488) and Goodreads Reviews/Users (*r* = 0.550). This suggests that Library *Print* Holdings is still providing stronger coefficients in relationship to other metric counts than total library holdings.

Findings for correlation trends suggest that unlike the results in previous studies, library print holdings can be used to *moderately* indicate various aspects of impact. But the interesting result is that scholarly research impact shown with Scopus Citations and cultural impact depicted by Goodreads Users and Ratings are probably more sensitive to the uptake of print version of books by libraries *in the short-term*. Educational curricula and book-sourced citations take much more time to take effect and become moderately related to print uptake of books *in the long-term* but always much more useful than library eholdings. Correspondingly, indicators accrued in long term (such as journal- or book-sourced formal citations, and syllabus mentions) can produce stronger correlation results with print holdings when zero counts are included in the dataset. However, metric counts accumulated online (all Goodreads-sourced metrics and altmetric indicators) do not indicate significant long-term improvement and can only produce stronger correlation with non-zero datasets, suggesting their limitations for assessment of all books.

In answer to the second RQ, various models based on LPH and LEH and a mixture of them were tested indicating that LPH has significantly better prediction results than LEH. The robustness of prediction models was tested over time and with varying mixture of metrics. Figure 2 highlighted a few interesting results. **Firstly**, the sudden jumps in library holdings, that have occurred for books from 2010 onwards, have not created a better predictability of metrics by eholdings, but have been surprisingly contemporary with a significant upgrade in the predictions made by print holdings particularly for Scopus Citations. This is likely to suggest that the expansion of library print collection have been in line with scholarly usage. It is, however, less known whether this results from libraries prioritizing to order print book when university research staff request for a title. Furthermore, R^2^ scores for Goodreads Users in both LPH and LEH predictor models show a sudden jump in 2009–2011, ending in 2015–2017 significantly above that of Scopus Citations (particularly for LPH), suggesting faster accumulation of online users for more recent books. **Secondly**, GB Citations prediction scores over time (max = 13%) with LPH were significantly lower than the average estimate without time scale (21.5%), suggesting that, despite lower variation within time periods, distinctions across time periods are broader in the extent of book-to-book citations accrued. Likewise, the overall prediction of Syllabus Mentions (23.7%) is stronger than that of most periods except for 2006–2008 (25%), suggesting that LPH is a better predictor of Syllabus Mentions after a longer time period, perhaps at least four (12%) to seven years (17%) after first publication date. These results are reasonable, because they clearly depict that for more recent books slow accumulation of citations results in weak explanation of variances by LPH; whilst these improve over time for LPH, the predictions with LEH hardly improve and always remain weak.

In answer to the third research question, prediction models suggested that there is a clear difference between LPH, LEH and TLH. LPH mainly reflects conventional aspects of book impact, and particularly depicts the importance of traditional research and educational impact, whereas LEH mainly associates with online engagement of users with books either in Goodreads or Mendeley. Nevertheless, TLH indicates aspects of impact inspired by both LPH and LEH, with varying patterns across fields, but overall balance slightly shifted in favor of online uptake of books, since the frequency of LEH counts is higher than LPH. With regards to Mendeley, results suggested that electronic availability of books significantly associates with their addition to Mendeley by users. The exceptions were Arts, Ethics and Religion, Law, Library Science and Bibliography, Philosophy, Psychology and Political Science, where LPH exceeded LEH (Online Appendix Table 20), which clearly suggest the lower application of Mendeley for Social Science and Humanities.

As with the fourth research question, along the explored dimensions, it was possible to suggest that major book indicators including Scopus citations, Google Books Citations, Syllabus Mentions, and Goodreads engagements were explicitly best explained by library print holding counts across almost all fields; however, *none* of the combinations of metrics can create particularly prominent results for predicting electronic holdings, even in the exceptional fields of Mathematics and Engineering and Technology, where there were trivial advantages in favor of library ebook holding counts (Table 28 in Online Appendix). However, success of *electronic* collection might be seen in fields that Goodreads, that has a better prediction of online impact, had the highest coefficients in *print holding prediction* compared to eholdings; these fields are Medicine, Library Science and Bibliographies, Mathematics, Education, and Ethics and Religion, since ebook holdings surpluses the effectiveness of print collection.

## Conclusion

Since it was generally overlooked how massive collection of ebooks in libraries affect the library holding counts, this research examined the relationship between library holdings across two formats and other metrics showing that library print holdings are more likely to indicate multiple aspects of impact. Librarians select book formats based on research and educational requests and sometimes financial priorities, however, their administration on e-book selection is more likely to be partial because they are primarily offered in packages by publishers and dealers (e.g. ProQuest or EBSCO). Although sometimes selection process in a library is likely to end up with the choice of an unneeded print book as much as an unused e-book, the extent to which this helps to measure an impactful library collection was less known. Consequently, this research investigated whether and how library holding formats can indicate impact or use from various perspectives.

The present study confirmed correlation levels observed for total library holdings in previous studies and contributed additional evidence that print holdings can moderately reflect research, educational and even online impact of books at higher level than total library holdings. It was also suggested that ebook holdings indicate only weak relationships, below that of total library holdings. As citation-like characteristics were observed in library holding data, it was normally seen that correlation levels for books had increased by age as more library holdings were accumulated. The results showed simultaneous dramatic jumps of library holding counts in 2010 for both print and particularly in sizable quantity for ebooks, while the correlation levels of Scopus Citations and Goodreads engagements only rose significantly with print holdings, and did not change with eholdings. This presumably suggests that print book acquisition is often in demand and effective, but current rate of library eholdings does not statistically imply significant levels of impact or use. This suggests that widespread availability of ebooks in libraries, despite probably in more circulation and use, cannot give enough statistical evidence to measure impact. Furthermore, inclusion of books with zero metric counts produces stronger correlation results with traditional citations and syllabus mentions, but decreases for all online impact indicators. Books without library print book holdings are perhaps less likely to have research and educational usages, while highly acquired print books in libraries may not necessarily indicate online impact and therefore online indicators have limited utility in impact assessment.

In scrutiny of differences between library print and ebook holdings, a broad range of metrics and fields were explored in this research. The overall results suggested that print holdings predominantly indicate traditional aspects of impact such as research and educational impact. Eholdings and total holdings, because of more numerous counts of eholdings than print holdings, are basically more likely to lean toward showing online engagements of users. The prediction of metrics could slightly improve if eholdings accompanied print holdings in the models but not for total holdings. Library print holdings alone were consistently more efficient than eholdings and total holdings in predicting book metric counts across fields and over time, except for Mendeley for which eholdings slightly prevailed. This apparently indicates that library ebooks are more likely to be saved in Mendeley than library print books.

In summary, as previous studies have suggested that library holdings for books are plenty to be considered as the most promising source of scholarly impact assessment (Torres-Salinas et al., 2017), current research suggests to draw a distinction between Library Print Holding counts and Library Eholding counts in terms of level of impact captured and aspects of impact. Print Holding counts is a promising indicator of book impact as it has both statistically and theoretically good underlying assumptions for prediction of book impact compared to total holdings or the accumulated count of print and electronic holdings that so far has been in use in previous studies. Library eholding statistics show a tendency to indicate online readership statistics as in Mendeley, but the current rate of impact associated with that is also weak. It is important to note that massive collection of ebooks in libraries has undermined the statistical results of predicting impact of books almost ubiquitously across all fields for both eholdings and total library holdings, and correspondingly this research proposed library print holding as a better indicator of book impact assessment instead of total library holdings.

There is a hope that this analysis will contribute evidence about how and where library holding metrics can be used to produce the best statistical results in book impact assessment. It particularly sounds more useful to see a growth in the studies that aim to understand “library ebook usage” as supported by for instance SUSHI and COUNTER protocols to investigate the usefulness of ebooks for scholarly book impact assessment.

## Supplementary Information

Below is the link to the electronic supplementary material.

Supplementary file1 (DOCX 521 KB)

## Footnotes

1 https://www.oclc.org/en/worldcat/inside-worldcat.html.

2 10.6084/m9.figshare.14593506.
