# Supplementary material for: Why does library holding format really matter for book impact assessment?: Modelling the relationship between citations and altmetrics with print and electronic holdings
Source: Scientometrics. 2021 Dec 20;127(2):1129–60. doi: 10.1007/s11192-021-04239-9 (PMC8686103; doi:10.1007/s11192-021-04239-9)
Supplement: Supplementary file 1 — Supplementary file1 (DOCX 521 KB) [file 11192_2021_4239_MOESM1_ESM.docx]

**Supplementary Tables and Figures to the Article:**

“Why Does Library Holding Format Really Matter for Book Impact Assessment?: Modelling the Relationship between Citations and Altmetrics with Print and Electronic Holdings”

Ashraf Maleki

*Senior Researcher, University of Turku, Turku, Finland*

*ashraf.maleki@utu.fi*

# Abbreviations

LPH: Library Print Holdings

LEH: Library Electronic Holdings

TLH: Total Library Holdings

GB: Google Books

SM: Syllabus Mentions

GU: Goodreads Users

GR: Goodreads Ratings

GTR: Goodreads Text Reviews

GAR: Goodreads Average Ratings

## Tables

**Correlation Analyses: Zero vs. Non-zero Metric Counts across 26 Fields**

[Table 1. Significant Spearman’s Correlation Coefficients between **Library Print Holdings (LPH)** and other metrics **With Zero Counts** for 26 fields 3](#_Toc70058340)

[Table 2. Significant Spearman’s Correlation Coefficients between **Library Print Holdings (LPH)** and other metrics **Without Zero Counts** for 26 fields 5](#_Toc70058341)

[Table 3. Significant Spearman’s Correlation Coefficients between **Library Electronic Holdings (LEH)** and other metrics **With Zero Counts** for 26 fields 7](#_Toc70058342)

[Table 4. Significant Spearman’s Correlation Coefficients between **Library Electronic Holdings (LEH)** and other metrics **Without Zero Counts** for 26 fields 9](#_Toc70058343)

[Table 5. Significant Spearman’s Correlation Coefficients between **Total Library Holdings (TLH)** and other metrics **With Zero Counts** for 26 fields 11](#_Toc70058344)

[Table 6. Significant Spearman’s Correlation Coefficients between **Total Library Holdings (TLH)** and other metrics **Without Zero Counts** for 26 fields 13](#_Toc70058345)

### Correlation Analyses: Trend in Six Fields

[Table 7. Spearman’s Correlation Coefficients between](#_Toc70058346) **[Library Print Holdings (LPH)](#_Toc70058346)** [and other metrics across six fields and over time (Dataset2) 15](#_Toc70058346)

[Table 8. Spearman’s Correlation Coefficients between **Library Electronic Holdings (LEH)** and other metrics across six fields and over time (Dataset2) 17](#_Toc70058347)

### Regression Analyses: Single-independent-variable Models across 26 Fields

[Table 9. Adjusted R](#_Toc70058348)^[2](#_Toc70058348)^ [of Linear Squares regression of](#_Toc70058348) **[Library Print Holdings (LPH)](#_Toc70058348)** [with other metrics each as a sole independant variable. 21](#_Toc70058348)

[Table 10. Adjusted R^2^ of Linear Squares regression of **Library Eholdings (LEH)** with other metrics each as a sole independant variable. 23](#_Toc70058349)

[Table 11. Adjusted R^2^ of Linear Squares regression of **Total Library Holdings (TLH)** with other metrics each as a sole independant variable. 25](#_Toc70058350)

**Regression Analyses: LPH and LEH Combined Predictor Models across 26 Fields**

[Table 12. Statistically significant linear regression standardized coefficients (**β**) in 26 fields. The dependent metric is **Scopus Citations** and predictors are LPH and LEH. 27](#_Toc70058351)

[Table 13. Statistically significant linear regression standardized coefficients (**β**) in 26 fields. The dependent metric is **Google Books Citations** and predictors are LPH and LEH. 28](#_Toc70058352)

[Table 14. Statistically significant linear regression standardized coefficients (**β**) in six sample fields. The dependent metric is **Syllabus Mentions (of opensyllabus.org)** and predictors are LPH and LEH. 29](#_Toc70058353)

[Table 15. Statistically significant linear regression standardized coefficients (**β**) in 26 fields. The dependent metric is **Syllabus Mentions (of altmetric.com)** and predictors are LPH and LEH. 29](#_Toc70058354)

[Table 16. Statistically significant linear regression standardized coefficients (**β**) in 26 fields. The dependent metric is **Goodreads Users (GU)** and predictors are LPH and LEH. 30](#_Toc70058355)

[Table 17. Statistically significant linear regression standardized coefficients (**β**) in 26 fields. The dependent metric is **Goodreads Ratings (GR)** and predictors are LPH and LEH. 31](#_Toc70058356)

[Table 18. Statistically significant linear regression standardized coefficients (**β**) in 26 fields. The dependent metric is **Goodreads Text Reviews (GTR)** and predictors are LPH and LEH. 32](#_Toc70058357)

[Table 19. Statistically significant linear regression standardized coefficients (**β**) in 26 fields. The dependent metric is **Goodreads Average Ratings (GAR)** and predictors are LPH and LEH. 33](#_Toc70058358)

[Table 20. Statistically significant linear regression standardized coefficients (**β**) in 26 fields. The dependent metric is **Mendeley Readers** and predictors are LPH and LEH. 34](#_Toc70058359)

[Table 21. Statistically significant linear regression standardized coefficients (**β**) in 26 fields. The dependent metric is **Twitter Users** and predictors are LPH and LEH. 35](#_Toc70058360)

[Table 22. Statistically significant linear regression standardized coefficients (**β**) in 26 fields. The dependent metric is **Facebook Walls** and predictors are LPH and LEH. 36](#_Toc70058361)

[Table 23. Statistically significant linear regression standardized coefficients (**β**) in 26 fields. The dependent metric is **Wikipedia Articles** and predictors are LPH and LEH. 37](#_Toc70058362)

[Table 24. Statistically significant linear regression standardized coefficients (**β**) in 26 fields. The dependent metric is **Blog Pages** and predictors are LPH and LEH. 38](#_Toc70058363)

[Table 25. Statistically significant linear regression standardized coefficients (**β**) in 26 fields. The dependent metric is **News Posts** and predictors are LPH and LEH. 39](#_Toc70058364)

### Regression Analyses: Multi-variate Models across 26 Fields

[Table 26. Statistically significant linear regression standardized coefficients (](#_Toc70058365)**[β](#_Toc70058365)**[) in 26 fields. The dependent metric is](#_Toc70058365) **[Total Library Holding](#_Toc70058365)**[. Only Goodreads Ratings is excluded from models due to causing collinearity. 40](#_Toc70058365)

[Table 27. Statistically significant linear regression standardized coefficients (**β**) in 26 fields. The dependent metric is **Library Print Holding**. Only Goodreads Ratings is excluded from models due to causing collinearity. 41](#_Toc70058366)

[Table 28. Statistically significant linear regression standardized coefficients (**β**) in 26 fields. The dependent metric is **Library Electronic Holding**. Only Goodreads Ratings is excluded from models due to causing collinearity. 42](#_Toc70058367)

## Figures

### Regression Models: Predictability of Metrics with LPH, LEH, and TLH across Six Fields

[Figure 1. Statistically significant R^2^ scores of regression models with LPH, LEH or TLH as independent variable and with one other metrics as the predicted variable in **Anthropology** over time 19](#_Toc70034008)

[Figure 2. Statistically significant R^2^ scores of regression models with LPH, LEH or TLH as independent variable and with one other metrics as the predicted variable in **Arts** over time 19](#_Toc70034009)

[Figure 3. Statistically significant R^2^ scores of regression models with LPH, LEH or TLH as independent variable and with one other metrics as the predicted variable in **Business and Economics** over time 19](#_Toc70034010)

[Figure 4. Statistically significant R^2^ scores of regression models with LPH, LEH or TLH as independent variable and with one other metrics as the predicted variable in **Law** over time 20](#_Toc70034011)

[Figure 5. Statistically significant R^2^ scores of regression models with LPH, LEH or TLH as independent variable and with one other metrics as the predicted variable in **Medicine** over time 20](#_Toc70034012)

[Figure 6. Statistically significant R^2^ scores of regression models with LPH, LEH or TLH as independent variable and with one other metrics as the predicted variable in **Political Science** over time 20](#_Toc70034013)

**Correlation Analyses: Zero vs. Non-zero Metric Counts across 26 Fields**

Table 1. Significant Spearman’s Correlation Coefficients between Library Print Holdings (LPH) and other metrics With Zero Counts for 26 fields

| **Fields\Metrics** | **Scopus** | **GB** | **SM** | **GU** | **GR** | **GTR** | **GAR** | **# of Books** |
| --- | --- | --- | --- | --- | --- | --- | --- | --- |
| Agriculture | 0.479 | 0.441 | 0.183 | 0.367 | 0.354 | 0.235 | 0.32 | 1,775 |
| Anthropology* | 0.43 | 0.394 | 0.266 | 0.364 | 0.402 | 0.346 | 0.28 | 1,198 |
| Arts* | 0.494 | 0.463 | 0.272 | 0.384 | 0.419 | 0.31 | 0.31 | 1,245 |
| Business and Economics* | 0.526 | 0.485 | 0.256 | 0.329 | 0.323 | 0.261 | 0.268 | 11,987 |
| Chemistry | 0.436 | 0.436 | 0.201 | 0.253 | 0.236 | 0.123 | 0.231 | 1,651 |
| Education | 0.417 | 0.307 | 0.213 | 0.336 | 0.361 | 0.287 | 0.292 | 4,475 |
| Engineering and Technology | 0.313 | 0.382 | 0.155 | 0.161 | 0.213 | 0.161 | 0.195 | 14,140 |
| Ethics and Religion | 0.566 | 0.458 | 0.307 | 0.471 | 0.474 | 0.365 | 0.279 | 4,354 |
| Geography | 0.463 | 0.46 | 0.225 | 0.342 | 0.368 | 0.251 | 0.322 | 1,367 |
| History | 0.485 | 0.452 | 0.292 | 0.426 | 0.434 | 0.344 | 0.28 | 8,798 |
| Languages and Literature | 0.457 | 0.452 | 0.31 | 0.377 | 0.381 | 0.267 | 0.278 | 9,798 |
| Law* | 0.551 | 0.442 | 0.278 | 0.498 | 0.449 | 0.314 | 0.408 | 4,734 |
| Library Science and Bibliography | 0.378 | 0.238 | 0.306 | 0.312 | 0.312 | 0.243 | 0.191 | 563 |
| Mathematics | 0.373 | 0.362 | 0.238 | 0.237 | 0.247 | 0.206 | 0.213 | 4,093 |
| Medicine* | 0.503 | 0.456 | 0.2 | 0.457 | 0.425 | 0.261 | 0.386 | 12,227 |
| Military Science | 0.542 | 0.592 | 0.293 | 0.485 | 0.46 | 0.399 | 0.385 | 822 |
| Music | 0.504 | 0.454 | 0.272 | 0.455 | 0.471 | 0.313 | 0.343 | 1,161 |
| Natural History and Biology | 0.537 | 0.457 | 0.301 | 0.474 | 0.462 | 0.339 | 0.415 | 1,640 |
| Philosophy | 0.550 | 0.454 | 0.276 | 0.448 | 0.446 | 0.332 | 0.32 | 1,935 |
| Physics | 0.371 | 0.41 | 0.262 | 0.323 | 0.382 | 0.294 | 0.348 | 2,628 |
| Physiology | 0.584 | 0.515 | 0.239 | 0.453 | 0.443 | 0.279 | 0.417 | 1,716 |
| Political Sciences* | 0.562 | 0.432 | 0.293 | 0.424 | 0.429 | 0.317 | 0.361 | 5,098 |
| Psychology | 0.502 | 0.456 | 0.244 | 0.355 | 0.385 | 0.333 | 0.262 | 1,996 |
| Recreation and Leisure | 0.442 | 0.385 | 0.264 | 0.349 | 0.374 | 0.244 | 0.234 | 777 |
| Social Sciences | 0.526 | 0.456 | 0.283 | 0.411 | 0.43 | 0.331 | 0.34 | 9,645 |
| Zoology | 0.507 | 0.361 | 0.275 | 0.509 | 0.52 | 0.371 | 0.439 | 758 |
| **Average of 26 fields** | **0.481** | **0.431** | **0.258** | **0.385** | **0.392** | **0.289** | **0.312** | **110,603** |
| **Average of six* fields** | **0.511** | **0.445** | **0.261** | **0.409** | **0.408** | **0.302** | **0.336** | **36,493** |

GB: Google Books Citations; SM: Syllabus Mentions; GU: Goodreads Users; GR: Goodreads Ratings; GTR: Goodreads Text Reviews; GAR: Goodreads Average Ratings; All coefficients significant at p<0.001. Gray cells: correlation is performed with altmetric.com syllabus mention results rather than opensyllabus.org explorer results.

Continuation for Table 1. Significant Spearman’s Correlation Coefficients between Library Print Holdings (LPH) and other metrics With Zero Counts for 26 fields.

| **Fields\Metrics** | **Mendeley** | **Twitter** | **Facebook** | **Wikipedia** | **Blogs** | **News** | **# of Books** |
| --- | --- | --- | --- | --- | --- | --- | --- |
| Agriculture | -0.089 | -0.131 | -0.111 | 0.076 |  |  | 1,775 |
| Anthropology* |  | -0.074* | -0.103 |  | 0.105 | 0.126 | 1,198 |
| Arts* | -0.069* | -0.127 | -0.143 |  |  | 0.057* | 1,245 |
| Business and Economics* | -0.058 | -0.074 | -0.093 | 0.035 | 0.064 | 0.116 | 11,987 |
| Chemistry |  | -0.092 | -0.092 | 0.100 | 0.057* | 0.087 | 1,651 |
| Education | -0.071 | -0.136 | -0.143 | 0.064 | 0.044 | 0.075 | 4,475 |
| Engineering and Technology | -0.099 | -0.111 | -0.128 | 0.043 | 0.026 | 0.053 | 14,140 |
| Ethics and Religion | 0.097 |  | -0.089 | 0.081 | 0.08 | 0.105 | 4,354 |
| Geography |  | -0.117 | -0.134 |  |  | 0.074 | 1,367 |
| History |  |  | -0.074 | 0.061 | 0.077 | 0.086 | 8,798 |
| Languages and Literature | -0.028 | -0.112 | -0.109 | 0.037 | 0.032 |  | 9,798 |
| Law* | 0.077 |  | -0.056 | 0.060 | 0.077 | 0.081 | 4,734 |
| Library Science and Bibliography | -0.132 | -0.212 | -0.256 |  |  |  | 563 |
| Mathematics | -0.052 | -0.052 | -0.097 | 0.077 |  | 0.098 | 4,093 |
| Medicine* |  | -0.051 | -0.072 | 0.079 | 0.081 | 0.132 | 12,227 |
| Military Science |  |  |  | 0.083* |  | 0.129 | 822 |
| Music | 0.070* | -0.111 | -0.111 |  |  |  | 1,161 |
| Natural History and Biology |  | -0.082 | -0.152 | 0.062* |  | 0.124 | 1,640 |
| Philosophy | 0.052* | -0.062 | -0.089 | 0.059 |  | 0.109 | 1,935 |
| Physics | 0.040* |  | -0.061 | 0.069 |  | 0.114 | 2,628 |
| Physiology | 0.125 |  |  | 0.113 | 0.095 | 0.169 | 1,716 |
| Political Sciences* |  | -0.054 | -0.097 | 0.058 | 0.109 | 0.116 | 5,098 |
| Psychology | 0.082 | -0.064 | -0.114 | 0.079 | 0.075 | 0.103 | 1,996 |
| Recreation and Leisure | -0.075* | -0.25 | -0.135 |  |  | 0.08* | 777 |
| Social Sciences | -0.026* | -0.084 | -0.105 | 0.070 | 0.069 | 0.106 | 9,645 |
| Zoology |  |  | -0.113 |  | 0.088 | 0.171 | 758 |
| **Average of 26 fields** | **-0.009** | **-0.105** | **-0.112** | **0.069** | **0.072** | **0.105** | **110,603** |
| **Average of six* fields** | **-0.017** | **-0.076** | **-0.094** | **0.058** | **0.087** | **0.105** | **36,493** |

Mendeley: Mendeley Readers; Twitter: Unique count of Twitter Users; Facebook: Unique count of Facebook Walls; Wikipedia: Wikipedia Articles; Blogs: Blog Posts; News: News Posts; All coefficients significant at p<0.001 except* p<0.05.

Table 2. Significant Spearman’s Correlation Coefficients between Library Print Holdings (LPH) and other metrics Without Zero Counts for 26 fields

| **Fields\Metrics** | **Scopus** | **GB** | **SM** | **GU** | **GR** | **GTR** | **GAR** | **# of Books** |
| --- | --- | --- | --- | --- | --- | --- | --- | --- |
| Agriculture | 0.343 | 0.417 |  | 0.487 | 0.447 | 0.229 | -0.173 | 1,775 |
| Anthropology* | 0.435 | 0.405 | 0.423 | 0.572 | 0.495 | 0.421 | -0.114 | 1,198 |
| Arts* | 0.419 | 0.359 |  | 0.548 | 0.480 | 0.378 | -0.148 | 1,245 |
| Business and Economics* | 0.474 | 0.403 | 0.399 | 0.481 | 0.451 | 0.412 | -0.100 | 11,987 |
| Chemistry | 0.406 | 0.386 |  | 0.386 | 0.543 | 0.574 |  | 1,651 |
| Education | 0.386 | 0.238 | 0.226 | 0.577 | 0.507 | 0.400 | -0.131 | 4,475 |
| Engineering and Technology | 0.338 | 0.343 | 0.400 | 0.476 | 0.446 | 0.319 | -0.074 | 14,140 |
| Ethics and Religion | 0.537 | 0.447 | 0.325 | 0.601 | 0.538 | 0.420 | -0.155 | 4,354 |
| Geography | 0.430 | 0.440 | 0.316 | 0.485 | 0.421 | 0.331 | -0.144 | 1,367 |
| History | 0.460 | 0.414 | 0.430 | 0.550 | 0.488 | 0.384 | -0.097 | 8,798 |
| Languages and Literature | 0.438 | 0.451 | 0.404 | 0.444 | 0.398 | 0.266 | -0.078 | 9,798 |
| Law* | 0.499 | 0.323 | 0.335 | 0.517 | 0.472 | 0.340 | -0.058* | 4,734 |
| Library Science and Bibliography | 0.450 | 0.239 | 0.487 | 0.557 | 0.494 | 0.462 |  | 563 |
| Mathematics | 0.338 | 0.364 | 0.430 | 0.454 | 0.364 | 0.234 |  | 4,093 |
| Medicine* | 0.444 | 0.375 | 0.332 | 0.543 | 0.492 | 0.362 | -0.101 | 12,227 |
| Military Science | 0.424 | 0.424 | 0.371 | 0.631 | 0.553 | 0.418 |  | 822 |
| Music | 0.504 | 0.454 | 0.343 | 0.540 | 0.462 | 0.236 |  | 1,161 |
| Natural History and Biology | 0.454 | 0.380 | 0.441 | 0.563 | 0.502 | 0.203 |  | 1,640 |
| Philosophy | 0.534 | 0.458 | 0.291 | 0.544 | 0.468 | 0.424 |  | 1,935 |
| Physics | 0.352 | 0.378 | 0.455 | 0.559 | 0.523 | 0.292 | -0.095* | 2,628 |
| Physiology | 0.536 | 0.468 | 0.508 | 0.592 | 0.528 | 0.320 |  | 1,716 |
| Political Sciences* | 0.504 | 0.376 | 0.381 | 0.541 | 0.499 | 0.418 | -0.067 | 5,098 |
| Psychology | 0.494 | 0.452 | 0.332 | 0.522 | 0.484 | 0.399 | -0.144 | 1,996 |
| Recreation and Leisure | 0.380 | 0.288 | 0.560 | 0.469 | 0.423 | 0.333 | -0.143 | 777 |
| Social Sciences | 0.485 | 0.394 | 0.367 | 0.584 | 0.531 | 0.364 | -0.078 | 9,645 |
| Zoology | 0.459 | 0.337 |  | 0.646 | 0.519 | 0.369 | -0.174 | 758 |
| **Average of 26 fields** | **0.443** | **0.385** | **0.389** | **0.533** | **0.482** | **0.358** | **-0.115** | **110,603** |
| **Average of six* fields** | **0.463** | **0.374** | **0.374** | **0.534** | **0.482** | **0.389** | **-0.098** | **36,493** |

GB: Google Books Citations; SM: Syllabus Mentions; GU: Goodreads Users; GR: Goodreads Ratings; GTR: Goodreads Text Reviews; GAR: Goodreads Average Ratings; All coefficients significant at p<0.001 except* p<0.05. Gray cells: correlation is performed with altmetric.com syllabus mention results rather than opensyllabus.org explorer results.

Continuation for Table 2. Significant Spearman’s Correlation Coefficients between Library Print Holdings (LPH) and other metrics Without Zero Counts for 26 fields

| **Fields\Metrics** | **Mendeley** | **Twitter** | **Facebook** | **Wikipedia** | **Blogs** | **News** | **# of Books** |
| --- | --- | --- | --- | --- | --- | --- | --- |
| Agriculture | 0.088* |  | -0.138* |  |  |  | 1,775 |
| Anthropology* | 0.205 | -0.109* |  |  | 0.359 |  | 1,198 |
| Arts* | 0.201 |  |  |  | 0.329* |  | 1,245 |
| Business and Economics* | 0.225 |  | -0.097* |  | 0.324 |  | 11,987 |
| Chemistry | 0.331 |  |  |  | 0.235* |  | 1,651 |
| Education | 0.190 | -0.086 |  |  | 0.232 |  | 4,475 |
| Engineering and Technology | 0.269 | -0.115 |  | 0.107* | 0.161 |  | 14,140 |
| Ethics and Religion | 0.386 | -0.109 |  |  | 0.226 |  | 4,354 |
| Geography | 0.249 | -0.113* |  |  |  |  | 1,367 |
| History | 0.336 | -0.098 | -0.116* | 0.098 | 0.132 | 0.172 | 8,798 |
| Languages and Literature | 0.248 | -0.195 |  |  | 0.188 |  | 9,798 |
| Law* | 0.302 | -0.134 |  |  | 0.091* |  | 4,734 |
| Library Science and Bibliography |  |  |  |  |  |  | 563 |
| Mathematics | 0.358 |  |  | 0.189 |  |  | 4,093 |
| Medicine* | 0.294 | 0.052 |  |  | 0.129 | 0.112* | 12,227 |
| Military Science | 0.192 | -0.187 |  |  |  |  | 822 |
| Music | 0.207 | -0.243 |  |  |  |  | 1,161 |
| Natural History and Biology | 0.228 | -0.123 |  |  |  |  | 1,640 |
| Philosophy | 0.444 |  |  |  | 0.312 |  | 1,935 |
| Physics | 0.323 |  |  |  |  |  | 2,628 |
| Physiology | 0.238 |  | 0.159* |  | 0.252* |  | 1,716 |
| Political Sciences* | 0.338 | -0.135 |  |  | 0.153 | 0.210 | 5,098 |
| Psychology | 0.276 | -0.132 |  |  | 0.204* |  | 1,996 |
| Recreation and Leisure | 0.201 | -0.298 |  |  |  |  | 777 |
| Social Sciences | 0.282 | -0.128 |  |  | 0.210 | 0.096* | 9,645 |
| Zoology | 0.157 | -0.233 |  |  |  |  | 758 |
| **Average of 26 fields** | **0.263** | **-0.14** | **-0.048** | **0.131** | **0.221** | **0.318** | **110,603** |
| **Average of six* fields** | **0.261** | **-0.082** | **-0.097** |  | **0.231** | **0.161** | **36,493** |

Mendeley: Mendeley Readers; Twitter: Unique count of Twitter Users; Facebook: Unique count of Facebook Walls; Wikipedia: Wikipedia Articles; Blogs: Blog Posts; News: News Posts; All coefficients significant at p<0.001 except* p<0.05.

Table 3. Significant Spearman’s Correlation Coefficients between Library Electronic Holdings (LEH) and other metrics With Zero Counts for 26 fields

| **Fields\Metrics** | **Scopus** | **GB** | **SM** | **GU** | **GR** | **GTR** | **GAR** | **# of Books** |
| --- | --- | --- | --- | --- | --- | --- | --- | --- |
| Agriculture | 0.193 |  | 0.117 | 0.204 | 0.182 | 0.113 | 0.168 | 1,775 |
| Anthropology* | 0.202 | 0.075 | 0.094 | 0.245 | 0.247 | 0.203 | 0.187 | 1,198 |
| Arts* | 0.280 | 0.098 | 0.100 | 0.307 | 0.307 | 0.180 | 0.281 | 1,245 |
| Business and Economics* | 0.229 | 0.028 | 0.114 | 0.260 | 0.237 | 0.184 | 0.202 | 11,987 |
| Chemistry | 0.166 | -0.089 | 0.052* | 0.229 | 0.156 | 0.072 | 0.157 | 1,651 |
| Education | 0.321 | 0.145 | 0.110 | 0.287 | 0.250 | 0.164 | 0.221 | 4,475 |
| Engineering and Technology | 0.316 | -0.018* | 0.086 | 0.377 | 0.275 | 0.150 | 0.267 | 14,140 |
| Ethics and Religion | 0.240 | 0.191 | 0.138 | 0.236 | 0.222 | 0.154 | 0.153 | 4,354 |
| Geography | 0.180 |  | 0.138 | 0.204 | 0.184 | 0.127 | 0.166 | 1,367 |
| History | 0.253 | 0.117 | 0.089 | 0.276 | 0.275 | 0.188 | 0.222 | 8,798 |
| Languages and Literature | 0.171 | 0.065 | 0.042 | 0.249 | 0.224 | 0.154 | 0.181 | 9,798 |
| Law* | 0.261 | 0.131 | 0.134 | 0.269 | 0.237 | 0.168 | 0.220 | 4,734 |
| Library Science and Bibliography | 0.302 | -0.118 | 0.129 | 0.303 | 0.263 | 0.216 | 0.254 | 563 |
| Mathematics | 0.281 | 0.044 | 0.129 | 0.388 | 0.315 | 0.209 | 0.285 | 4,093 |
| Medicine* | 0.226 | 0.074 | 0.063 | 0.164 | 0.118 | 0.078 | 0.110 | 12,227 |
| Military Science | 0.143 |  |  | 0.199 | 0.221 | 0.201 | 0.194 | 822 |
| Music | 0.269 | 0.095 | 0.098 | 0.273 | 0.274 | 0.166 | 0.237 | 1,161 |
| Natural History and Biology | 0.230 | 0.114 | 0.108 | 0.309 | 0.300 | 0.219 | 0.281 | 1,640 |
| Philosophy | 0.209 | 0.149 | 0.08 | 0.252 | 0.250 | 0.156 | 0.218 | 1,935 |
| Physics | 0.261 | 0.093 | 0.119 | 0.281 | 0.216 | 0.137 | 0.208 | 2,628 |
| Physiology | 0.191 | 0.103 | 0.078 | 0.161 | 0.137 | 0.099 | 0.128 | 1,716 |
| Political Sciences* | 0.271 | 0.136 | 0.135 | 0.266 | 0.261 | 0.186 | 0.223 | 5,098 |
| Psychology | 0.327 | 0.094 | 0.159 | 0.274 | 0.267 | 0.217 | 0.208 | 1,996 |
| Recreation and Leisure | 0.161 |  | 0.169 | 0.369 | 0.349 | 0.304 | 0.251 | 777 |
| Social Sciences | 0.262 | 0.057 | 0.123 | 0.273 | 0.265 | 0.216 | 0.219 | 9,645 |
| Zoology | 0.145 |  | 0.11 | 0.326 | 0.299 | 0.226 | 0.241 | 758 |
| **Average of 26 fields** | **0.234** | **0.075** | **0.109** | **0.269** | **0.244** | **0.173** | **0.211** | **110,603** |
| **Average of six* fields** | **0.245** | **0.09** | **0.107** | **0.252** | **0.235** | **0.167** | **0.204** | **36,493** |

GB: Google Books Citations; SM: Syllabus Mentions; GU: Goodreads Users; GR: Goodreads Ratings; GTR: Goodreads Text Reviews; GAR: Goodreads Average Ratings; All coefficients significant at p<0.001 except* p<0.05. Gray cells: correlation is performed with altmetric.com syllabus mention results rather than opensyllabus.org explorer results.

Continuation for Table 3. Significant Spearman’s Correlation Coefficients between Library Electronic Holdings (LEH) and other metrics With Zero Counts for 26 fields

| **Fields\Metrics** | **Mendeley** | **Twitter** | **Facebook** | **Wikipedia** | **Blogs** | **News** | **# of Books** |
| --- | --- | --- | --- | --- | --- | --- | --- |
| Agriculture | 0.105 | 0.071 | 0.053* | 0.088 |  |  | 1,775 |
| Anthropology* | -0.124 | -0.142 | -0.105 |  |  |  | 1,198 |
| Arts* | -0.095 | -0.071* | -0.128 |  |  |  | 1,245 |
| Business and Economics* | 0.105 | 0.056 |  | 0.046 | 0.052 | 0.055 | 11,987 |
| Chemistry | 0.257 | 0.184 | 0.096 | 0.096 | 0.049* | 0.089 | 1,651 |
| Education | 0.100 |  |  | 0.085 | 0.032* | 0.054 | 4,475 |
| Engineering and Technology | 0.294 | 0.198 | 0.089 | 0.116 | 0.096 | 0.089 | 14,140 |
| Ethics and Religion | -0.092 | -0.07 | -0.082 |  | -0.033* |  | 4,354 |
| Geography | 0.105 |  |  | 0.060 |  |  | 1,367 |
| History | -0.150 | -0.115 | -0.093 |  |  |  | 8,798 |
| Languages and Literature | -0.113 | -0.082 | -0.068 | 0.039 |  |  | 9,798 |
| Law* | 0.067 | -0.057 | -0.049 | 0.058 | 0.045 | -0.032* | 4,734 |
| Library Science and Bibliography | -0.111 | 0.129 |  |  |  | 0.083* | 563 |
| Mathematics | 0.266 | 0.157 | 0.031* | 0.108 | 0.040 | 0.082 | 4,093 |
| Medicine* | 0.183 | 0.050 |  | 0.081 | 0.076 | 0.102 | 12,227 |
| Military Science | 0.070* |  |  |  |  |  | 822 |
| Music | -0.195 | -0.191 | -0.150 |  |  |  | 1,161 |
| Natural History and Biology | 0.055* |  | -0.083 | 0.076 |  | 0.079 | 1,640 |
| Philosophy | -0.088 | -0.099 | -0.134 |  |  |  | 1,935 |
| Physics | 0.222 | 0.102 | 0.039* | 0.123 |  | 0.095 | 2,628 |
| Physiology | 0.129 | 0.059* |  | 0.064 |  | 0.068 | 1,716 |
| Political Sciences* | -0.039 | -0.058 | -0.070 | 0.029 |  |  | 5,098 |
| Psychology | 0.074 | 0.071 |  | 0.057 | 0.066 |  | 1,996 |
| Recreation and Leisure | -0.170 | -0.166 | -0.100 | 0.095 |  |  | 777 |
| Social Sciences | -0.052 | -0.044 | -0.046 | 0.048 | 0.031 | 0.038 | 9,645 |
| Zoology |  |  | -0.097 | 0.075 |  |  | 758 |
| **Average of 26 fields** | **0.032** | **-0.001** | **-0.050** | **0.075** | **0.045** | **0.067** | **110,603** |
| **Average of six* fields** | **0.016** | **-0.037** | **-0.088** | **0.054** | **0.058** | **0.042** | **36,493** |

Mendeley: Mendeley Readers; Twitter: Unique count of Twitter Users; Facebook: Unique count of Facebook Walls; Wikipedia: Wikipedia Articles; Blogs: Blog Posts; News: News Posts; All coefficients significant at p<0.001 except* p<0.05.

Table 4. Significant Spearman’s Correlation Coefficients between Library Electronic Holdings (LEH) and other metrics Without Zero Counts for 26 fields

| **Fields\Metrics** | **Scopus** | **GB** | **SM** | **GU** | **GR** | **GTR** | **GAR** | **# of Books** |
| --- | --- | --- | --- | --- | --- | --- | --- | --- |
| Agriculture | 0.129 |  |  | 0.174 | 0.161 |  |  | 1,775 |
| Anthropology* | 0.107 |  |  | 0.213 | 0.164 |  | -0.119 | 1,198 |
| Arts* | 0.181 |  |  | 0.264 | 0.115 |  |  | 1,245 |
| Business and Economics* | 0.192 |  |  | 0.279 | 0.268 | 0.118 | -0.059 | 11,987 |
| Chemistry | 0.112 | -0.124 |  | 0.143 |  |  |  | 1,651 |
| Education | 0.210 | 0.090 |  | 0.200 | 0.188 | 0.126 | -0.079 | 4,475 |
| Engineering and Technology | 0.199 | -0.112 |  | 0.225 | 0.236 | 0.157 | -0.051* | 14,140 |
| Ethics and Religion | 0.158 | 0.155 |  | 0.198 | 0.159 | 0.064* | -0.075 | 4,354 |
| Geography | 0.168 |  |  | 0.220 | 0.215 |  |  | 1,367 |
| History | 0.149 | 0.074 |  | 0.238 | 0.171 | 0.076 |  | 8,798 |
| Languages and Literature | 0.070 |  | -0.105 | 0.239 | 0.178 | 0.126 |  | 9,798 |
| Law* | 0.186 | 0.061 |  | 0.219 | 0.194 |  |  | 4,734 |
| Library Science and Bibliography | 0.200 | -0.130 |  | 0.156 |  |  |  | 563 |
| Mathematics | 0.239 | -0.040* |  | 0.197 | 0.270 | 0.178 | -0.080 | 4,093 |
| Medicine* | 0.151 |  |  | 0.068 | 0.082 | 0.129 |  | 12,227 |
| Military Science | 0.117* |  |  | 0.237 | 0.183 |  |  | 822 |
| Music | 0.152 | 0.089 |  | 0.169 | 0.154 | 0.179 |  | 1,161 |
| Natural History and Biology | 0.229 | 0.061* |  | 0.285 | 0.281 | 0.175* |  | 1,640 |
| Philosophy | 0.096 | 0.136 |  | 0.176 | 0.136 |  |  | 1,935 |
| Physics | 0.146 | -0.061* |  | 0.129 | 0.103* |  |  | 2,628 |
| Physiology | 0.122 |  |  | 0.111 |  |  |  | 1,716 |
| Political Sciences* | 0.221 | 0.115 | 0.115* | 0.236 | 0.210 | 0.112 | -0.064 | 5,098 |
| Psychology | 0.269 |  |  | 0.225 | 0.219 | 0.222 | -0.072* | 1,996 |
| Recreation and Leisure | 0.178 |  |  | 0.375 | 0.330 | 0.188* |  | 777 |
| Social Sciences | 0.204 | 0.030 |  | 0.304 | 0.264 | 0.168 |  | 9,645 |
| Zoology | 0.151 |  |  | 0.272 | 0.305 | 0.240 |  | 758 |
| **Average of 26 fields** | **0.167** | **0.025** | **0.005** | **0.214** | **0.199** | **0.151** | **-0.075** | **110,603** |
| **Average of six* fields** | **0.173** | **0.088** | **0.115** | **0.213** | **0.172** | **0.12** | **-0.081** | **36,493** |

GB: Google Books Citations; SM: Syllabus Mentions; GU: Goodreads Users; GR: Goodreads Ratings; GTR: Goodreads Text Reviews; GAR: Goodreads Average Ratings; All coefficients significant at p<0.001 except* p<0.05. Gray cells: correlation is performed with altmetric.com syllabus mention results rather than opensyllabus.org explorer results.

Continuation for Table 4. Significant Spearman’s Correlation Coefficients between Library Electronic Holdings (LEH) and other metrics Without Zero Counts for 26 fields

| **Fields\Metrics** | **Mendeley** | **Twitter** | **Facebook** | **Wikipedia** | **Blogs** | **News** | **# of Books** |
| --- | --- | --- | --- | --- | --- | --- | --- |
| Agriculture | 0.160 |  | -0.161* |  |  |  | 1,775 |
| Anthropology* |  |  |  |  |  |  | 1,198 |
| Arts* | 0.169 |  |  |  |  |  | 1,245 |
| Business and Economics* | 0.209 | -0.074 |  |  | 0.128 |  | 11,987 |
| Chemistry |  |  |  |  |  |  | 1,651 |
| Education | 0.252 | -0.060* | 0.142 |  | 0.316 |  | 4,475 |
| Engineering and Technology | 0.155 | -0.140 |  | 0.140 | 0.160 |  | 14,140 |
| Ethics and Religion | 0.201 | -0.115 |  |  |  | 0.216 | 4,354 |
| Geography | 0.242 |  |  |  |  |  | 1,367 |
| History | 0.141 | -0.088 |  |  |  |  | 8,798 |
| Languages and Literature | 0.174 | -0.148 | -0.112* |  |  |  | 9,798 |
| Law* | 0.261 | -0.249 |  |  |  |  | 4,734 |
| Library Science and Bibliography | 0.213 |  |  |  |  |  | 563 |
| Mathematics | 0.222 |  | -0.167 |  |  |  | 4,093 |
| Medicine* | 0.188 |  |  |  | 0.223 | 0.178 | 12,227 |
| Military Science |  |  | 0.410* |  |  |  | 822 |
| Music |  | -0.221 |  |  |  |  | 1,161 |
| Natural History and Biology | 0.186 | -0.162 |  |  |  |  | 1,640 |
| Philosophy | 0.166 |  |  |  |  |  | 1,935 |
| Physics | 0.163 |  |  |  | 0.170* |  | 2,628 |
| Physiology | 0.098 | -0.189 | -0.154* |  | 0.244* |  | 1,716 |
| Political Sciences* | 0.151 | -0.181 |  |  |  |  | 5,098 |
| Psychology | 0.171 |  |  |  |  |  | 1,996 |
| Recreation and Leisure | 0.167 | -0.200 |  |  |  |  | 777 |
| Social Sciences | 0.203 | -0.113 |  |  | 0.104 | 0.096* | 9,645 |
| Zoology | 0.127* |  |  |  |  |  | 758 |
| **Average of 26 fields** | **0.183** | **-0.149** | **-0.007** | **0.140** | **0.192** | **-0.128** | **110,603** |
| **Average of six* fields** | **0.196** | **-0.168** |  |  | **0.176** | **0.178** | **36,493** |

Mendeley: Mendeley Readers; Twitter: Unique count of Twitter Users; Facebook: Unique count of Facebook Walls; Wikipedia: Wikipedia Articles; Blogs: Blog Posts; News: News Posts; All coefficients significant at p<0.001 except* p<0.05.

Table 5. Significant Spearman’s Correlation Coefficients between Total Library Holdings (TLH) and other metrics With Zero Counts for 26 fields

| **Fields\Metrics** | **Scopus** | **GB** | **SM** | **GU** | **GR** | **GTR** | **GAR** | **# of Books** |
| --- | --- | --- | --- | --- | --- | --- | --- | --- |
| Agriculture | 0.278 | 0.151 | 0.157 | 0.267 | 0.254 | 0.174 | 0.227 | 1,775 |
| Anthropology* | 0.308 | 0.240 | 0.177 | 0.312 | 0.333 | 0.287 | 0.231 | 1,198 |
| Arts* | 0.407 | 0.278 | 0.183 | 0.393 | 0.421 | 0.282 | 0.359 | 1,245 |
| Business and Economics* | 0.379 | 0.245 | 0.198 | 0.311 | 0.300 | 0.243 | 0.249 | 11,987 |
| Chemistry | 0.259 | 0.077 | 0.125 | 0.278 | 0.225 | 0.111 | 0.220 | 1,651 |
| Education | 0.402 | 0.252 | 0.167 | 0.335 | 0.317 | 0.234 | 0.267 | 4,475 |
| Engineering and Technology | 0.376 | 0.111 | 0.126 | 0.368 | 0.298 | 0.176 | 0.286 | 14,140 |
| Ethics and Religion | 0.380 | 0.336 | 0.216 | 0.348 | 0.343 | 0.257 | 0.213 | 4,354 |
| Geography | 0.309 | 0.208 | 0.183 | 0.277 | 0.282 | 0.203 | 0.247 | 1,367 |
| History | 0.370 | 0.288 | 0.179 | 0.371 | 0.380 | 0.284 | 0.271 | 8,798 |
| Languages and Literature | 0.306 | 0.259 | 0.150 | 0.329 | 0.315 | 0.223 | 0.240 | 9,798 |
| Law* | 0.400 | 0.270 | 0.202 | 0.391 | 0.352 | 0.254 | 0.322 | 4,734 |
| Library Science and Bibliography | 0.381 |  | 0.219 | 0.350 | 0.325 | 0.263 | 0.273 | 563 |
| Mathematics | 0.372 | 0.180 | 0.201 | 0.414 | 0.355 | 0.250 | 0.318 | 4,093 |
| Medicine* | 0.326 | 0.195 | 0.122 | 0.281 | 0.244 | 0.164 | 0.218 | 12,227 |
| Military Science | 0.239 | 0.213 | 0.097 | 0.258 | 0.286 | 0.270 | 0.239 | 822 |
| Music | 0.381 | 0.257 | 0.180 | 0.375 | 0.386 | 0.252 | 0.299 | 1,161 |
| Natural History and Biology | 0.351 | 0.243 | 0.200 | 0.395 | 0.392 | 0.289 | 0.358 | 1,640 |
| Philosophy | 0.356 | 0.308 | 0.158 | 0.358 | 0.361 | 0.254 | 0.291 | 1,935 |
| Physics | 0.352 | 0.230 | 0.186 | 0.340 | 0.306 | 0.217 | 0.285 | 2,628 |
| Physiology | 0.309 | 0.236 | 0.155 | 0.281 | 0.273 | 0.182 | 0.251 | 1,716 |
| Political Sciences* | 0.411 | 0.280 | 0.211 | 0.358 | 0.359 | 0.269 | 0.302 | 5,098 |
| Psychology | 0.445 | 0.315 | 0.222 | 0.340 | 0.352 | 0.310 | 0.248 | 1,996 |
| Recreation and Leisure | 0.280 | 0.204 | 0.215 | 0.408 | 0.407 | 0.347 | 0.270 | 777 |
| Social Sciences | 0.384 | 0.235 | 0.201 | 0.347 | 0.354 | 0.288 | 0.280 | 9,645 |
| Zoology | 0.267 | 0.180 | 0.175 | 0.405 | 0.391 | 0.293 | 0.316 | 758 |
| **Average of 26 fields** | **0.347** | **0.232** | **0.177** | **0.342** | **0.331** | **0.245** | **0.272** | **110,603** |
| **Average of six* fields** | **0.372** | **0.251** | **0.182** | **0.341** | **0.335** | **0.25** | **0.28** | **36,493** |

GB: Google Books Citations; SM: Syllabus Mentions; GU: Goodreads Users; GR: Goodreads Ratings; GTR: Goodreads Text Reviews; GAR: Goodreads Average Ratings; All coefficients significant at p<0.001. Gray cells: correlation is performed with altmetric.com syllabus mention results rather than opensyllabus.org explorer results.

Continuation for Table 5. Significant Spearman’s Correlation Coefficients between Total Library Holdings (TLH) and other metrics With Zero Counts for 26 fields

| **Fields\Metrics** | **Mendeley** | **Twitter** | **Facebook** | **Wikipedia** | **Blogs** | **News** | **# of Books** |
| --- | --- | --- | --- | --- | --- | --- | --- |
| Agriculture |  |  |  | 0.083 |  |  | 1,775 |
| Anthropology* | -0.091 | -0.166 | -0.141 |  | 0.070* |  | 1,198 |
| Arts* | -0.102 | -0.127 | -0.180 | 0.057* |  |  | 1,245 |
| Business and Economics* | 0.074 |  | -0.036 | 0.055 | 0.071 | 0.082 | 11,987 |
| Chemistry | 0.201 | 0.095 |  | 0.117 | 0.058* | 0.099 | 1,651 |
| Education | 0.065 | -0.072 | -0.063 | 0.088 | 0.034* | 0.061 | 4,475 |
| Engineering and Technology | 0.231 | 0.133 | 0.035 | 0.112 | 0.091 | 0.089 | 14,140 |
| Ethics and Religion | -0.050 | -0.075 | -0.107 |  |  | 0.038* | 4,354 |
| Geography |  | -0.104 | -0.080 |  |  | 0.055* | 1,367 |
| History | -0.128 | -0.114 | -0.117 |  |  | 0.028 | 8,798 |
| Languages and Literature | -0.137 | -0.152 | -0.122 | 0.042 |  | -0.026* | 9,798 |
| Law* | 0.071 | -0.076 | -0.067 | 0.067 | 0.064 |  | 4,734 |
| Library Science and Bibliography | -0.155 |  |  |  |  | 0.093* | 563 |
| Mathematics | 0.216 | 0.116 |  | 0.123 | 0.033* | 0.103 | 4,093 |
| Medicine* | 0.141 |  | -0.029 | 0.089 | 0.083 | 0.119 | 12,227 |
| Military Science |  |  |  |  |  |  | 822 |
| Music | -0.133 | -0.214 | -0.181 |  |  |  | 1,161 |
| Natural History and Biology |  | -0.049* | -0.136 | 0.074 |  | 0.104 | 1,640 |
| Philosophy | -0.072 | -0.121 | -0.164 |  |  |  | 1,935 |
| Physics | 0.203 | 0.065 |  | 0.134 |  | 0.111 | 2,628 |
| Physiology | 0.097 |  | -0.071 | 0.077 | 0.053* | 0.100 | 1,716 |
| Political Sciences* | -0.036* | -0.099 | -0.111 | 0.04 | 0.047 | 0.031* | 5,098 |
| Psychology | 0.081 |  | -0.080 | 0.096 | 0.079 | 0.068 | 1,996 |
| Recreation and Leisure | -0.193 | -0.290 | -0.154 | 0.1 |  |  | 777 |
| Social Sciences | -0.069 | -0.108 | -0.094 | 0.063 | 0.043 | 0.060 | 9,645 |
| Zoology | -0.089* | -0.101 | -0.136 | 0.079* |  | 0.087* | 758 |
| **Average of 26 fields** | **0.006** | **-0.077** | **-0.102** | **0.083** | **0.061** | **0.072** | **110,603** |
| **Average of six* fields** | **0.010** | **-0.117** | **-0.094** | **0.062** | **0.067** | **0.077** | **36,493** |

Mendeley: Mendeley Readers; Twitter: Unique count of Twitter Users; Facebook: Unique count of Facebook Walls; Wikipedia: Wikipedia Articles; Blogs: Blog Posts; News: News Posts; All coefficients significant at p<0.001 except* p<0.05.

Table 6. Significant Spearman’s Correlation Coefficients between Total Library Holdings (TLH) and other metrics Without Zero Counts for 26 fields

| **Fields\Metrics** | **Scopus** | **GB** | **SM** | **GU** | **GR** | **GTR** | **GAR** | **# of Books** |
| --- | --- | --- | --- | --- | --- | --- | --- | --- |
| Agriculture | 0.198 | 0.135 |  | 0.317 | 0.308 | 0.240 | -0.102* | 1,775 |
| Anthropology* | 0.235 | 0.184 |  | 0.375 | 0.326 | 0.235 | -0.149 | 1,198 |
| Arts* | 0.293 | 0.197 |  | 0.409 | 0.271 | 0.253 |  | 1,245 |
| Business and Economics* | 0.329 | 0.204 | 0.141 | 0.404 | 0.397 | 0.274 | -0.086 | 11,987 |
| Chemistry | 0.239 |  |  | 0.296 | 0.361 | 0.435* |  | 1,651 |
| Education | 0.310 | 0.196 |  | 0.355 | 0.338 | 0.284 | -0.117 | 4,475 |
| Engineering and Technology | 0.301 | 0.052 | 0.274 | 0.337 | 0.355 | 0.265 | -0.064 | 14,140 |
| Ethics and Religion | 0.304 | 0.311 | 0.119* | 0.371 | 0.335 | 0.238 | -0.118 | 4,354 |
| Geography | 0.311 | 0.179 |  | 0.371 | 0.357 | 0.329 |  | 1,367 |
| History | 0.286 | 0.250 | 0.163 | 0.395 | 0.337 | 0.248 | -0.068 | 8,798 |
| Languages and Literature | 0.226 | 0.210 | 0.066* | 0.355 | 0.299 | 0.226 | -0.032* | 9,798 |
| Law* | 0.321 | 0.187 | 0.114* | 0.362 | 0.346 | 0.225 |  | 4,734 |
| Library Science and Bibliography | 0.320 |  | 0.414 | 0.333 | 0.288 | 0.245 |  | 563 |
| Mathematics | 0.320 | 0.109 | 0.273 | 0.333 | 0.364 | 0.244 |  | 4,093 |
| Medicine* | 0.267 | 0.138 | 0.180 | 0.255 | 0.282 | 0.281 | -0.074 | 12,227 |
| Military Science | 0.246 | 0.093* |  | 0.372 | 0.313 |  |  | 822 |
| Music | 0.310 | 0.273 |  | 0.352 | 0.322 | 0.240 |  | 1,161 |
| Natural History and Biology | 0.323 | 0.183 |  | 0.423 | 0.412 | 0.212 |  | 1,640 |
| Philosophy | 0.259 | 0.302 | 0.173* | 0.339 | 0.280 | 0.236 |  | 1,935 |
| Physics | 0.263 | 0.095 |  | 0.308 | 0.283 | 0.194 | -0.089* | 2,628 |
| Physiology | 0.279 | 0.161 | 0.276* | 0.322 | 0.319 | 0.196* | -0.117* | 1,716 |
| Political Sciences* | 0.353 | 0.253 | 0.227 | 0.392 | 0.364 | 0.302 | -0.074 | 5,098 |
| Psychology | 0.422 | 0.287 |  | 0.387 | 0.391 | 0.374 | -0.119 | 1,996 |
| Recreation and Leisure | 0.273 | 0.173 |  | 0.470 | 0.429 | 0.270 |  | 777 |
| Social Sciences | 0.334 | 0.209 | 0.187 | 0.449 | 0.415 | 0.297 | -0.043 | 9,645 |
| Zoology | 0.267 | 0.118 |  | 0.422 | 0.413 | 0.327 | -0.141* | 758 |
| **Average of 26 fields** | **0.292** | **0.187** | **0.201** | **0.366** | **0.343** | **0.267** | **-0.093** | **110,603** |
| **Average of six* fields** | **0.300** | **0.194** | **0.166** | **0.366** | **0.331** | **0.262** | **-0.096** | **36,493** |

GB: Google Books Citations; SM: Syllabus Mentions; GU: Goodreads Users; GR: Goodreads Ratings; GTR: Goodreads Text Reviews; GAR: Goodreads Average Ratings; All coefficients significant at p<0.001 except* p<0.05. Gray cells: correlation is performed with altmetric.com syllabus mention results rather than opensyllabus.org explorer results.

Continuation for Table 6. Significant Spearman’s Correlation Coefficients between Total Library Holdings (TLH) and other metrics Without Zero Counts for 26 fields

| **Fields\Metrics** | **Mendeley** | **Twitter** | **Facebook** | **Wikipedia** | **Blogs** | **News** | **# of Books** |
| --- | --- | --- | --- | --- | --- | --- | --- |
| Agriculture | 0.208 |  | -0.183 |  |  |  | 1,775 |
| Anthropology* | 0.211 | -0.126* |  |  |  |  | 1,198 |
| Arts* | 0.296 |  |  |  |  |  | 1,245 |
| Business and Economics* | 0.287 | -0.086 |  |  | 0.270 |  | 11,987 |
| Chemistry | 0.175 |  |  |  |  |  | 1,651 |
| Education | 0.308 | -0.088 | 0.151 |  | 0.313 |  | 4,475 |
| Engineering and Technology | 0.222 | -0.160 |  | 0.149 | 0.202 |  | 14,140 |
| Ethics and Religion | 0.353 | -0.139 |  |  |  | 0.159* | 4,354 |
| Geography | 0.311 | -0.144 |  |  | 0.231* |  | 1,367 |
| History | 0.307 | -0.124 |  |  |  |  | 8,798 |
| Languages and Literature | 0.250 | -0.233 | -0.119* |  |  |  | 9,798 |
| Law* | 0.358 | -0.258 |  |  |  |  | 4,734 |
| Library Science and Bibliography |  |  |  |  |  |  | 563 |
| Mathematics | 0.327 |  | -0.143* |  |  |  | 4,093 |
| Medicine* | 0.273 | -0.043* |  | 0.087* | 0.230 | 0.170 | 12,227 |
| Military Science | 0.187 |  | 0.410* |  |  |  | 822 |
| Music | 0.116* | -0.301 |  |  |  |  | 1,161 |
| Natural History and Biology | 0.239 | -0.196 |  |  |  |  | 1,640 |
| Philosophy | 0.373 | -0.102* | -0.194* |  |  |  | 1,935 |
| Physics | 0.250 |  |  |  |  |  | 2,628 |
| Physiology | 0.183 | -0.232 |  |  | 0.286 |  | 1,716 |
| Political Sciences* | 0.306 | -0.198 |  |  | 0.103* |  | 5,098 |
| Psychology | 0.335 | -0.118 |  |  | 0.194* |  | 1,996 |
| Recreation and Leisure | 0.213 | -0.311 |  |  |  |  | 777 |
| Social Sciences | 0.326 | -0.150 |  |  | 0.178 | 0.117 | 9,645 |
| Zoology | 0.194 | -0.157* |  |  |  |  | 758 |
| **Average of 26 fields** | **0.264** | **-0.167** | **-0.013** | **0.118** | **0.223** | **0.149** | **110,603** |
| **Average of six* fields** | **0.289** | **-0.142** |  | **0.087** | **0.201** | **0.170** | **36,493** |

Mendeley: Mendeley Readers; Twitter: Unique count of Twitter Users; Facebook: Unique count of Facebook Walls; Wikipedia: Wikipedia Articles; Blogs: Blog Posts; News: News Posts; All coefficients significant at p<0.001 except* p<0.05.

### Correlation Analyses: Trend in Six Fields

Table 7. Spearman’s Correlation Coefficients between Library Print Holdings (LPH) and other metrics across six fields and over time (Dataset2)

| **Metrics** | **Subject** | **<2003** | **2003-2005** | **2006-2008** | **2009-2011** | **2012-2014** | **2015-2017** |
| --- | --- | --- | --- | --- | --- | --- | --- |
| Scopus Citations | Anthropology |  | 0.305* | 0.290 | 0.327 | 0.353 | **0.374** |
|  | Arts | 0.347 |  | 0.276 | **0.479** | 0.333 | 0.266 |
|  | Economics | 0.18 | 0.451 | 0.386 | **0.543** | 0.520 | 0.371 |
|  | Law | 0.303 | 0.371 | 0.425 | **0.574** | 0.492 | 0.413 |
|  | Medicine | 0.215 | 0.226 | 0.334 | 0.473 | **0.512** | 0.342 |
|  | Political Sciences | 0.351 | 0.335 | 0.291 | **0.565** | 0.542 | 0.478 |
| Google Books Citations | Anthropology | 0.337 | 0.41 | **0.439** | 0.217 | 0.181 | 0.224 |
|  | Arts | 0.443 | **0.664** | 0.266 | 0.276 | 0.328 | 0.242 |
|  | Economics | 0.337 | **0.414** | 0.372 | 0.378 | 0.363 | 0.277 |
|  | Law | 0.388 | 0.394 | **0.419** | 0.388 | 0.344 | 0.347 |
|  | Medicine | 0.088 | 0.302 | 0.281 | 0.410 | **0.432** | 0.365 |
|  | Political Sciences | **0.508** | 0.326 | 0.344 | 0.345 | 0.243 | 0.314 |
| Syllabus Mentions | Anthropology | 0.505 | 0.596 | **0.415** | 0.373 | 0.389 |  |
|  | Arts | **0.549** | 0.508 | 0.276 | 0.466 | 0.303 |  |
|  | Economics | 0.424 | **0.572** | 0.478 | 0.479 | 0.432 | 0.218 |
|  | Law | 0.367 | 0.336 | 0.416 | **0.458** | 0.402 | 0.173 |
|  | Medicine | 0.531 | 0.523 | 0.504 | **0.547** | 0.425 | 0.163 |
|  | Political Sciences | 0.470 | **0.601** | 0.509 | 0.516 | 0.485 | 0.21 |
| Goodreads Users | Anthropology | 0.192* | 0.374 | 0.418 | 0.430 | **0.439** | 0.393 |
|  | Arts | 0.328 |  | 0.420 | **0.475** | 0.438 | 0.217 |
|  | Economics | 0.195 | 0.361 | 0.276 | **0.401** | 0.361 | 0.29 |
|  | Law | 0.438 | **0.503** | 0.388 | 0.483 | 0.409 | 0.317 |
|  | Medicine | 0.403 | 0.359 | 0.357 | **0.428** | 0.408 | 0.297 |
|  | Political Sciences | 0.235 | 0.453 | **0.498** | 0.472 | 0.461 | 0.363 |
| Goodreads Reviews | Anthropology |  | 0.366* | 0.369 | 0.417 | **0.434** | 0.4 |
|  | Arts | 0.318 |  | 0.416 | **0.475** | 0.414 | 0.258 |
|  | Economics | 0.197 | 0.387 | 0.271 | **0.43** | 0.427 | 0.333 |
|  | Law | 0.435 | 0.49 | 0.428 | **0.538** | 0.485 | 0.381 |
|  | Medicine | 0.429 | 0.332 | 0.334 | 0.444 | **0.466** | 0.389 |
|  | Political Sciences | 0.265 | 0.394 | 0.43 | 0.484 | **0.493** | 0.375 |
| Goodreads Text Reviews | Anthropology |  | 0.308* | 0.326 | 0.366 | **0.392** | 0.352 |
|  | Arts | **0.427** |  | 0.347 | 0.368 | 0.332 | 0.16 |
|  | Economics | 0.226 | 0.326 | 0.258 | **0.312** | 0.31 | 0.271 |
|  | Law | 0.326 | 0.222 | 0.324 | 0.307 | **0.343** | 0.293 |
|  | Medicine | 0.264 | 0.264 | 0.233 | **0.300** | 0.281 | 0.21 |
|  | Political Sciences | 0.260 | **0.442** | 0.390 | 0.324 | 0.358 | 0.268 |
| Goodreads Average Ratings | Anthropology |  |  | 0.265* | 0.271 | 0.303 | 0.333 |
|  | Arts |  |  | **0.388** | 0.236 | 0.326 | 0.19 |
|  | Economics | 0.167 | 0.279 | 0.175 | **0.330** | 0.305 | 0.266 |
|  | Law | 0.352 | **0.437** | 0.323 | 0.435 | 0.364 | 0.298 |
|  | Medicine | 0.322 | 0.301 | 0.295 | 0.368 | **0.385** | 0.284 |
|  | Political Sciences | 0.158 | 0.366 | 0.365 | 0.39 | **0.394** | 0.337 |
| Altmetric Total Posts | Anthropology | **0.256** |  |  | 0.133* | 0.137 |  |
|  | Arts |  |  | **0.288** | 0.208 | 0.147 | -0.125 |
|  | Economics | 0.23 | **0.316** | 0.239 | 0.292 | 0.227 | 0.149 |
|  | Law | 0.237 | 0.175 | 0.287 | **0.337** | 0.246 | 0.112 |
|  | Medicine | 0.242 | 0.175 | 0.169 | 0.265 | **0.300** | 0.226 |
|  | Political Sciences | 0.22 | 0.286 | 0.234 | 0.299 | **0.302** | 0.089 |
| Mendeley Readers | Anthropology | **0.239** |  |  |  |  | -0.144* |
|  | Arts |  |  | 0.272 |  |  | **-0.301** |
|  | Economics | 0.092 |  |  | **0.157** | 0.039* | -0.123 |
|  | Law |  |  |  | **0.214** | 0.151 |  |
|  | Medicine | **0.103** |  |  | 0.065 | 0.043* | -0.059 |
|  | Political Sciences |  |  |  | **0.185** | 0.116 | -0.107 |
| Twitter Unique Users | Anthropology | **0.194*** |  |  |  |  |  |
|  | Arts |  |  | **0.313** | 0.192 |  | -0.138 |
|  | Economics | **0.190** | 0.172 | 0.133 | 0.186 | 0.147 | 0.131 |
|  | Law | 0.189 | 0.198 | 0.195 | **0.209** | 0.146 | 0.082 |
|  | Medicine | 0.104 | 0.113 | 0.155 | 0.178 | 0.237 | **0.208** |
|  | Political Sciences | 0.145 |  | **0.270** | 0.173 | 0.221 | 0.054* |
| Facebook Unique Users | Anthropology |  | **-0.311*** | -0.216* |  |  |  |
|  | Arts |  |  |  |  |  |  |
|  | Economics |  |  |  | **0.061** |  | 0.045* |
|  | Law |  |  |  |  |  |  |
|  | Medicine | 0.104 | 0.079* | 0.058* | 0.105 | 0.094 | **0.113** |
|  | Political Sciences | **0.127*** |  |  |  | 0.061* |  |
| Wikipedia Article Citations | Anthropology |  |  |  |  |  |  |
|  | Arts |  |  |  |  |  |  |
|  | Economics | **0.104** |  |  | 0.045* | 0.085 |  |
|  | Law |  |  |  | 0.064* | **0.085** | 0.066* |
|  | Medicine | **0.102** |  |  |  | 0.098 | 0.038* |
|  | Political Sciences |  | **0.152*** |  |  | 0.102 |  |
| Blog Mentions | Anthropology | **0.325** |  |  | 0.168 |  | 0.138* |
|  | Arts |  |  | **0.204*** |  |  |  |
|  | Economics | 0.152 | 0.138 | 0.114 | **0.226** | 0.225 | 0.193 |
|  | Law | 0.188 |  | 0.173 | 0.181 | 0.187 | **0.194** |
|  | Medicine | 0.116 | 0.119 | 0.147 | 0.195 | 0.197 | **0.181** |
|  | Political Sciences | 0.142 | **0.235** | 0.141* | 0.205 | 0.222 | 0.225 |
| News Posts | Anthropology | **0.302** |  |  |  | 0.142 |  |
|  | Arts |  |  |  |  | 0.100* |  |
|  | Economics | **0.151** | 0.104* | 0.062* | 0.109 | 0.144 | 0.149 |
|  | Law |  |  | **0.177** | 0.076 | 0.109 | 0.108 |
|  | Medicine | 0.085 | **0.160** | 0.086 | 0.129 | 0.142 | 0.124 |
|  | Political Sciences |  | 0.153* | 0.142* | 0.133 | **0.225** | 0.19 |

All coefficients are significant at p<0.001 except *p<0.05. Bold coefficients represent the strongest year for each field.

Table 8. Spearman’s Correlation Coefficients between Library Electronic Holdings (LEH) and other metrics across six fields and over time (Dataset2)

| **Metrics** | **Subject** | **<2003** | **2003-2005** | **2006-2008** | **2009-2011** | **2012-2014** | **2015-2017** |
| --- | --- | --- | --- | --- | --- | --- | --- |
| Scopus Citations | Anthropology |  |  |  |  |  | **0.25** |
|  | Arts | **0.357** |  | 0.301 | 0.184 |  | 0.237 |
|  | Economics | **0.27** | 0.114 | 0.068* | 0.149 | 0.17 | 0.074 |
|  | Law | 0.147* |  |  | **0.191** | 0.161 | 0.091 |
|  | Medicine | 0.196 | 0.233 | 0.063* | 0.081 | **0.245** | 0.114 |
|  | Political Sciences | **0.341** | 0.151* |  | 0.187 | 0.172 | 0.136 |
| Google Books Citations | Anthropology |  |  |  |  |  | *0.162* |
|  | Arts |  |  |  |  |  | *0.127* |
|  | Economics | 0.09 | **0.111*** |  |  |  | -0.103 |
|  | Law |  | **-0.204** |  |  | 0.051* | 0.142 |
|  | Medicine | -0.125 |  | -0.075 |  | **0.149** |  |
|  | Political Sciences | 0.167 | **0.195*** |  |  |  |  |
| Syllabus Mentions | Anthropology | **0.185*** |  |  |  |  | 0.17 |
|  | Arts |  |  |  |  |  | **0.118*** |
|  | Economics | **0.254** | 0.143 |  | 0.11 | 0.133 | 0.057 |
|  | Law | 0.128* |  |  | 0.096 | **0.146** | 0.06 |
|  | Medicine | **0.17** |  | -0.092 |  | 0.109 | 0.058 |
|  | Political Sciences | 0.153 | **0.179*** |  | 0.113 | **0.179** |  |
| Goodreads Users | Anthropology | **0.326** |  |  | 0.302 | 0.146 | 0.199 |
|  | Arts |  | **0.428** | 0.303 | 0.286 | 0.19 | 0.284 |
|  | Economics | **0.334** | 0.226 | 0.128 | 0.149 | 0.22 | 0.189 |
|  | Law | **0.197** | 0.152* |  | 0.177 | 0.217 | 0.189 |
|  | Medicine | **0.22** | 0.075* |  |  | 0.143 |  |
|  | Political Sciences | **0.282** |  | 0.262 | 0.19 | 0.238 | 0.217 |
| Goodreads Reviews | Anthropology | **0.33** |  |  | 0.264 | 0.128 | 0.275 |
|  | Arts | 0.253 | **0.423** | 0.348 | 0.313 | 0.149 | 0.306 |
|  | Economics | **0.363** | 0.204 | 0.152 | 0.157 | 0.257 | 0.186 |
|  | Law | 0.236 |  |  | 0.17 | 0.233 | **0.244** |
|  | Medicine | **0.281** | 0.139 | 0.058* |  | 0.188 | 0.07 |
|  | Political Sciences | **0.373** |  | 0.224 | 0.166 | 0.246 | 0.261 |
| Text Reviews | Anthropology | **0.277** |  |  | 0.233 | 0.161 | 0.165 |
|  | Arts |  | **0.430** |  | 0.248 | 0.116* | 0.157 |
|  | Economics | **0.244** |  | 0.092 | 0.154 | 0.207 | 0.157 |
|  | Law | 0.151* | **0.184*** | 0.173 | 0.103 | 0.168 | 0.181 |
|  | Medicine | **0.122** | 0.074* |  | 0.054 | 0.113 |  |
|  | Political Sciences | 0.125* | **0.214** | 0.176 | 0.145 | 0.206 | 0.136 |
| Goodreads Average Ratings | Anthropology | **0.327** |  |  | 0.209 |  | 0.181 |
|  | Arts |  |  | **0.312** | 0.236 | 0.184 | 0.279 |
|  | Economics | **0.325** | 0.179 | 0.085 | 0.112 | 0.171 | 0.173 |
|  | Law | 0.182 |  |  | 0.145 | **0.198** | 0.181 |
|  | Medicine | **0.215** | 0.08* |  |  | 0.131 |  |
|  | Political Sciences | **0.275** |  | 0.223 | 0.16 | 0.185 | 0.202 |
| Altmetric Total Posts | Anthropology |  | **-0.382** |  | -0.172 |  |  |
|  | Arts |  |  |  |  |  |  |
|  | Economics | **0.201** | 0.097* | 0.118 | 0.143 | 0.184 | 0.096 |
|  | Law | **0.135*** |  | 0.125 | 0.074* | 0.064 |  |
|  | Medicine | **0.298** | 0.232 | 0.113* | 0.101 | 0.183 | 0.076 |
|  | Political Sciences | **0.205** |  |  | 0.102 | 0.056* | 0.058* |
| Mendeley Readers | Anthropology |  |  |  | **-0.190** | -0.25 |  |
|  | Arts |  |  | -0.269 | **-0.279** | -0.112* |  |
|  | Economics | 0.128 |  | 0.143 | 0.057 | 0.111 | **0.183** |
|  | Law |  |  |  | 0.059* | 0.063* | **0.084** |
|  | Medicine | **0.268** | 0.272 | 0.235 | 0.054 | 0.136 | 0.265 |
|  | Political Sciences |  |  | **-0.148** |  | -0.105 | 0.098 |
| Twitter Unique Users | Anthropology |  | **-0.423** |  | -0.252 | -0.121* |  |
|  | Arts |  |  |  |  |  |  |
|  | Economics | 0.129 |  | 0.101 | 0.101 | **0.165** | 0.091 |
|  | Law |  |  |  |  |  |  |
|  | Medicine | 0.202 | **0.222** | 0.119 | 0.103 | 0.146 | 0.066 |
|  | Political Sciences |  |  |  | **0.062*** |  |  |
| Facebook Unique Users | Anthropology |  |  |  |  | **-0.113*** |  |
|  | Arts |  |  |  |  |  | **-0.119*** |
|  | Economics |  |  |  | 0.058 | 0.055 | **0.059** |
|  | Law |  |  |  |  |  |  |
|  | Medicine | **0.142** | 0.091 | 0.085 | 0.104 | 0.064 | 0.054 |
|  | Political Sciences |  |  |  |  |  |  |
| Wikipedia Article Citations | Anthropology |  |  |  |  |  | **0.150*** |
|  | Arts |  |  |  |  |  |  |
|  | Economics | **0.064*** |  |  | 0.043* | 0.061 |  |
|  | Law |  |  |  |  | 0.052* | **0.084** |
|  | Medicine | **0.130** | 0.099 | 0.079 |  | 0.061 | 0.057 |
|  | Political Sciences |  |  | **-0.143*** |  |  |  |
| Blog Mentions | Anthropology |  |  |  |  |  |  |
|  | Arts |  |  | **-0.210*** |  |  |  |
|  | Economics | 0.066* | 0.094* | **0.118** | 0.050* | 0.087 |  |
|  | Law |  |  |  |  |  |  |
|  | Medicine | 0.139 | **0.142** | 0.064* | 0.113 | 0.126 | 0.071 |
|  | Political Sciences |  |  |  |  |  |  |
| News Posts | Anthropology |  |  |  |  |  |  |
|  | Arts |  |  |  |  |  |  |
|  | Economics | 0.068* |  |  | 0.077 | **0.089** | 0.069 |
|  | Law |  |  |  |  | 0.053* | **0.058*** |
|  | Medicine | 0.071* | 0.139 | 0.055* | 0.093 | **0.103** |  |
|  | Political Sciences |  |  | **0.116*** | 0.074 |  |  |

All coefficients are significant at p<0.001 except *p<0.05. Bold coefficients represent the strongest year for each field. Negative coefficients are shown in red.

### Regression Models: Predictability of Metrics with LPH, LEH, and TLH across Six Fields


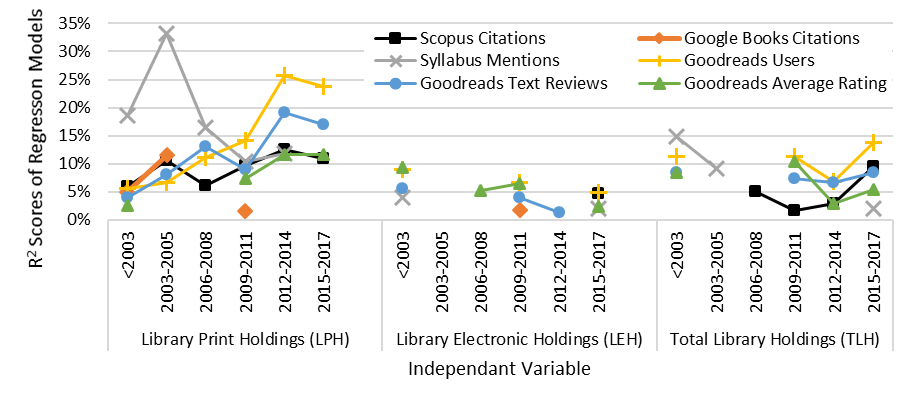


Figure 1. Statistically significant R^2^ scores of regression models with LPH, LEH or TLH as independent variable and with one other metrics as the predicted variable in Anthropology over time


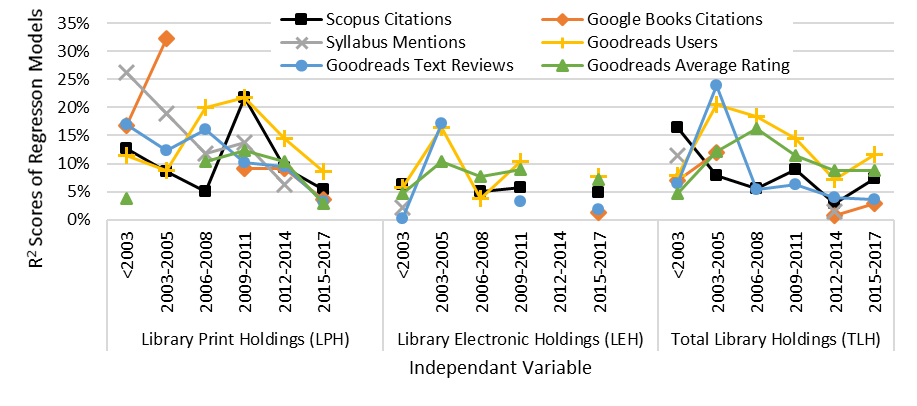


Figure 2. Statistically significant R^2^ scores of regression models with LPH, LEH or TLH as independent variable and with one other metrics as the predicted variable in Arts over time


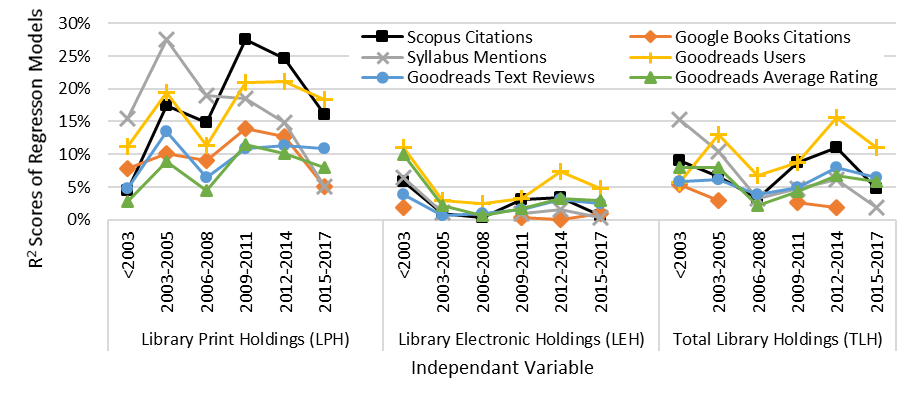


Figure 3. Statistically significant R^2^ scores of regression models with LPH, LEH or TLH as independent variable and with one other metrics as the predicted variable in Business and Economics over time


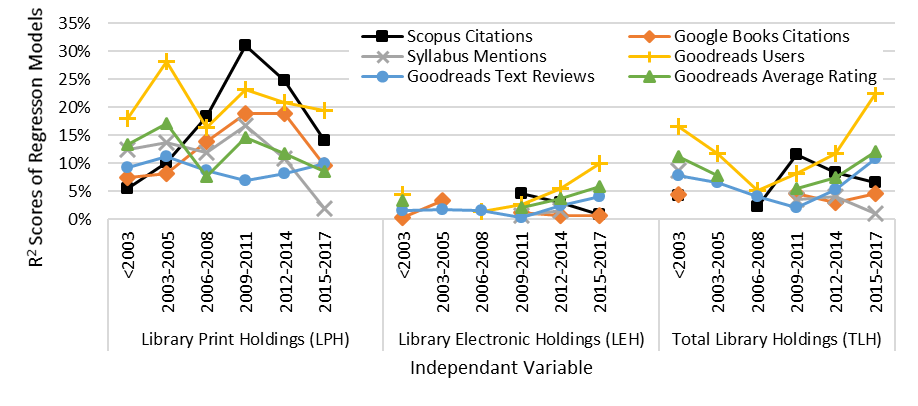


Figure 4. Statistically significant R^2^ scores of regression models with LPH, LEH or TLH as independent variable and with one other metrics as the predicted variable in Law over time


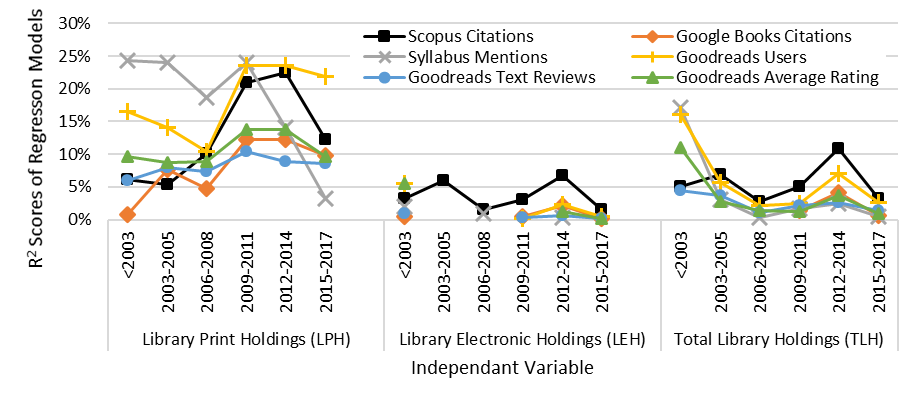


Figure 5. Statistically significant R^2^ scores of regression models with LPH, LEH or TLH as independent variable and with one other metrics as the predicted variable in Medicine over time


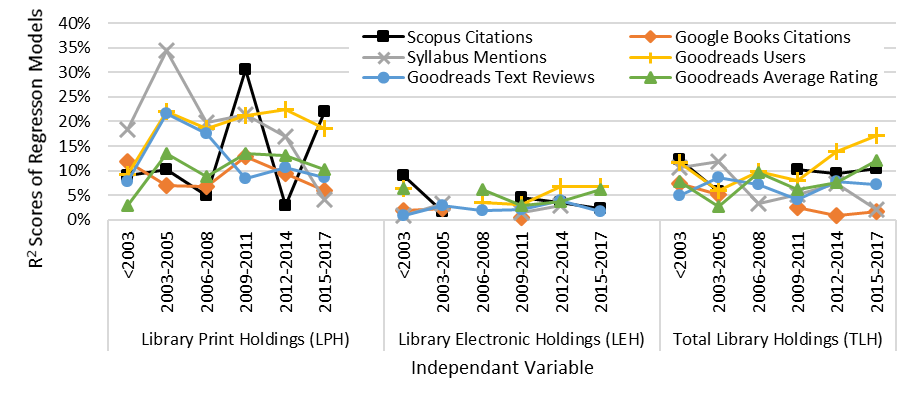


Figure 6. Statistically significant R^2^ scores of regression models with LPH, LEH or TLH as independent variable and with one other metrics as the predicted variable in Political Science over time

### Regression Analyses: Single-independent-variable Models across 26 Fields

Table 9. Adjusted R^2^ of Linear Squares regression of Library Print Holdings (LPH) with other metrics each as a sole independant variable.

| **Fields\Metrics** | **Scopus** | **GB** | **SM** | **GU** | **GR** | **GTR** | **GAR** | **# of Books** |
| --- | --- | --- | --- | --- | --- | --- | --- | --- |
| Agriculture | **20.8%** | 19.5% |  | 15.9% | 13.1% | 5.0% | 10.5% | 1,775 |
| Anthropology | 17.2% | 16.7% | **25.1%** | 16.2% | 18.0% | 12.6% | 8.5% | 1,198 |
| Arts | 21.0% | 21.1% | **21.2%** | 17.1% | 18.6% | 11.1% | 9.8% | 1,245 |
| Business and Economics | **26.2%** | 24.5% | 23.2% | 14.3% | 12.7% | 7.5% | 7.4% | 11,987 |
| Chemistry | **19.9%** | 17.6% |  | 9.3% | 8.0% | 2.0% | 6.0% | 1,651 |
| Education | **17.4%** | 12.8% |  | 17.2% | 17.1% | 9.9% | 9.1% | 4,475 |
| Engineering and Technology | 11.1% | **13.2%** |  | 6.5% | 6.6% | 2.9% | 4.0% | 14,140 |
| Ethics and Religion | **26.0%** | 20.2% |  | 21.5% | 22.2% | 14.4% | 9.5% | 4,354 |
| Geography | **20.6%** | 19.1% |  | 13.7% | 13.5% | 6.3% | 10.1% | 1,367 |
| History | **19.5%** | 19.0% |  | 18.5% | 19.2% | 12.3% | 9.4% | 8,798 |
| Languages and Literature | 17.5% | **19.3%** |  | 14.1% | 13.8% | 6.9% | 7.9% | 9,798 |
| Law | **26.6%** | 24.2% | 19.6% | 23.2% | 18.1% | 8.6% | 14.4% | 4,734 |
| Library Science and Bibliography | **16.6%** | 6.3% |  | 15.7% | 15.7% | 10.0% | 5.0% | 563 |
| Mathematics | **14.5%** | 13.7% |  | 9.6% | 8.8% | 4.8% | 4.7% | 4,093 |
| Medicine | 23.7% | 20.2% | **26.3%** | 23.4% | 19.1% | 7.2% | 15.0% | 12,227 |
| Military Science | 27.0% | **35.8%** |  | 23.1% | 19.1% | 12.3% | 14.5% | 822 |
| Music | **22.3%** | 21.2% |  | 19.8% | 20.4% | 8.6% | 12.8% | 1,161 |
| Natural History and Biology | **28.1%** | 21.3% |  | 24.6% | 21.0% | 10.1% | 17.2% | 1,640 |
| Philosophy | **24.3%** | 17.8% |  | 19.8% | 19.5% | 12.0% | 11.0% | 1,935 |
| Physics | 14.5% | **16.7%** |  | 15.5% | 15.8% | 8.5% | 12.2% | 2,628 |
| Physiology | **31.5%** | 26.7% |  | 25.7% | 21.8% | 8.5% | 18.2% | 1,716 |
| Political Sciences | **28.5%** | 22.3% | 26.6% | 19.0% | 17.7% | 9.6% | 11.5% | 5,098 |
| Psychology | **24.3%** | 20.0% |  | 16.4% | 17.7% | 11.7% | 8.6% | 1,996 |
| Recreation and Leisure | **19.7%** | 17.7% |  | 13.2% | 14.5% | 6.7% | 7.9% | 777 |
| Social Sciences | **26.3%** | 23.0% |  | 20.3% | 19.7% | 10.5% | 12.2% | 9,645 |
| Zoology | 24.8% | 12.8% |  | **27.0%** | 25.2% | 12.3% | 19.9% | 758 |
| **Total** | 18.0% | 21.3% | **23.9%** | 19.6% | 17.4% | 9.3% | 12.4% | 110,603 |

GB: Google Books Citations; SM: Syllabus Mentions; GU: Goodreads Users; GR: Goodreads Ratings; GTR: Goodreads Text Reviews; GAR: Goodreads Average Ratings; All R^2^s are significant at p<0.001; Empty cells are not tested.

Continuation for Table 9. Adjusted R^2^ of Linear Squares regression of Library Print Holdings (LPH) with other metrics each as a sole independant variable.

| **Fields\Metrics** | **Mendeley** | **Twitter** | **Facebook** | **Wikipedia** | **Blogs** | **News** | **# of Books** |
| --- | --- | --- | --- | --- | --- | --- | --- |
| Agriculture | 0.6%** | 1.0% | 1.1% | 0.4%** |  |  | 1,775 |
| Anthropology* |  |  | 1.1% |  | 2.5% | 1.6% | 1,198 |
| Arts* | 0.4%* | 0.5%** | 1.4% |  | 0.3%* |  | 1,245 |
| Business and Economics* |  | 0.1% | 0.6% | 0.2% | 2.1% | 0.6% | 11,987 |
| Chemistry | 0.5%** | 0.4%** | 0.3%* | 0.9% | 0.9% |  | 1,651 |
| Education | 0.2% | 1.0% | 1.2% | 0.3% | 0.9% | 0.2%** | 4,475 |
| Engineering and Technology |  | 0.5% | 1.0% | 0.2% | 0.4% | 0.1% | 14,140 |
| Ethics and Religion | 1.5% | 0.6%** | 0.4% | 0.6% | 1.2% | 0.8% | 4,354 |
| Geography |  |  | 1.0% |  | 0.7%** |  | 1,367 |
| History | 0.3% |  | 0.3% | 0.4% | 0.7% | 0.6% | 8,798 |
| Languages and Literature |  | 1.0% | 0.8% | 0.1%** | 0.0%* | 0.1% | 9,798 |
| Law* | 1.5% |  |  | 0.4% | 0.9% | 0.4% | 4,734 |
| Library Science and Bibliography | 1.2%** | 2.3% | 3.5% |  |  |  | 563 |
| Mathematics |  |  | 0.5% | 0.7% | 1.1% |  | 4,093 |
| Medicine* | 0.2% |  | 0.2% | 0.7% | 1.6% | 0.5% | 12,227 |
| Military Science |  |  |  | 0.7%* | 1.6% |  | 822 |
| Music | 1.3% | 1.1% | 0.7%** | 0.3%* |  |  | 1,161 |
| Natural History and Biology |  | 0.5%** | 1.7% | 0.3%* | 1.5% |  | 1,640 |
| Philosophy | 0.9% |  | 0.6% | 0.3%** | 1.1% |  | 1,935 |
| Physics | 0.7% |  | 0.1%* | 0.6% | 1.0% |  | 2,628 |
| Physiology | 1.9% |  |  | 1.1% | 3.0% | 0.6%** | 1,716 |
| Political Sciences* | 0.3% | 0.1%* | 0.3% | 0.3% | 1.4% | 1.1% | 5,098 |
| Psychology | 1.0% | 0.2%* | 1.0% | .4%** | 1.5% | 0.5%** | 1,996 |
| Recreation and Leisure |  | 4.8% | 1.0%** |  | 0.7%* |  | 777 |
| Social Sciences | 0.1%** | 0.3% | 0.5% | 0.5% | 1.5% | 0.6% | 9,645 |
| Zoology |  | 0.4%* | 0.8%** |  | 2.2% |  | 758 |
| **Average of 26 fields** | **0.8%** | **0.9%** | **0.9%** | **0.5%** | **1.3%** | **0.6%** | **110,603** |
| **Average of six* fields** | **0.6%** | **0.2%** | **0.7%** | **0.4%** | **1.5%** | **0.8%** | **36,493** |

Mendeley: Mendeley Readers; Twitter: Unique count of Twitter Users; Facebook: Unique count of Facebook Walls; Wikipedia: Wikipedia Articles; Blogs: Blog Posts; News: News Posts; All R^2^s are significant at p<0.001 except* p<0.05 and ** p<0.01.

Table 10. Adjusted R^2^ of Linear Squares regression of Library Eholdings (LEH) with other metrics each as a sole independant variable.

| **Fields\Metrics** | **Scopus** | **GB** | **SM** | **GU** | **GR** | **GTR** | **GAR** | **# of Books** |
| --- | --- | --- | --- | --- | --- | --- | --- | --- |
| Agriculture | **4.6%** | - |  | **4.6%** | 3.1% | 0.9% | 3.4% | 1,775 |
| Anthropology | 2.9% | 0.1% | 0.9% | **5.0%** | 4.2% | 2.4% | 4.1% | 1,198 |
| Arts | 5.9% | 0.6% | 0.8% | 5.6% | 4.3% | 1.6% | **6.0%** | 1,245 |
| Business and Economics | 4.2% | 0% | 2.4% | **6.9%** | 5.3% | 2.5% | 4.2% | 11,987 |
| Chemistry | 3.2% | 0.7% |  | **4.1%** | 1.4% | 0.4% | 2.0% | 1,651 |
| Education | **8.8%** | 3.7% |  | 7.7% | 5.3% | 2.0% | 5.4% | 4,475 |
| Engineering and Technology | 9.7% | 0.1% |  | **10.8%** | 5.4% | 1.4% | 6.7% | 14,140 |
| Ethics and Religion | **5.1%** | 2.9% |  | 4.7% | 3.7% | 1.6% | 3.2% | 4,354 |
| Geography | 1.6% | - |  | **3.7%** | 2.5% | 0.8% | 2.7% | 1,367 |
| History | 5.5% | 1.1% |  | **6.4%** | 5.5% | 2.5% | 5.8% | 8,798 |
| Languages and Literature | 2.3% | 0.1% |  | **5.7%** | 4.3% | 2.1% | 3.6% | 9,798 |
| Law | 6.4% | 2.3% | 3.4% | **6.6%** | 4.9% | 2.2% | 4.8% | 4,734 |
| Library Science and Bibliography | 8.0% | 1.2% |  | **8.9%** | 6.5% | 4.1% | 7.0% | 563 |
| Mathematics | 6.0% | - |  | **12.1%** | 7.7% | 2.9% | 9.0% | 4,093 |
| Medicine | **4.7%** | 0.4% | 0.2% | 1.5% | 0.7% | 0.5% | 1.0% | 12,227 |
| Military Science | 2.8% | 0.5%* |  | **3.9%** | 3.6% | 2.2% | **3.9%** | 822 |
| Music | 5.4% | 0.5% |  | **7.0%** | 6.6% | 2.8% | 6.7% | 1,161 |
| Natural History and Biology | 2.5% | 0.3%* |  | **7.7%** | 5.6% | 2.8% | 6.2% | 1,640 |
| Philosophy | 3.2% | 1.7% |  | **5.4%** | 4.8% | 2.1% | 5.0% | 1,935 |
| Physics | **8.6%** | 0.8% |  | 6.9% | 3.6% | 1.5% | 4.5% | 2,628 |
| Physiology | **3.7%** | 1.2% |  | 1.7% | 0.9% | 0.5%* | 1.3% | 1,716 |
| Political Sciences | **7.7%** | 2.5% | 4.8% | 6.7% | 6.1% | 2.9% | 5.4% | 5,098 |
| Psychology | **9.6%** | 1.1% |  | 6.9% | 6.6% | 3.9% | 5.3% | 1,996 |
| Recreation and Leisure | 2.5% | - |  | **12.1%** | 10.9% | 7.5% | 7.3% | 777 |
| Social Sciences | 6.0% | 0.3% |  | **7.3%** | 6.7% | 3.8% | 5.1% | 9,645 |
| Zoology | 0.7%* | - |  | **9.1%** | 6.9% | 3.5% | 5.8% | 758 |
| Total | 4.9% | 0.5% | 1.6% | **7.3%** | 5.1% | 2.3% | 5.4% | 110,603 |

* significant at p<0.05, All other R^2^s are significant at P<0.001; – shows insignificant regression results; Empty cells are not tested; Percentages in Red font are greater for LEH than their equivalent percentages for LPH.

Continuation for Table 10. Adjusted R^2^ of Linear Squares regression of Library Eholdings (LEH) with other metrics each as a sole independant variable.

| **Fields\Metrics** | **Mendeley** | **Twitter** | **Facebook** | **Wikipedia** | **Blogs** | **News** | **# of Books** |
| --- | --- | --- | --- | --- | --- | --- | --- |
| Agriculture | 3.4% | 1.3% | 0.6% | 0.7% | 0.3%* | 0.3%* | 1,775 |
| Anthropology* | 0.3%* | 0.8%** |  |  |  |  | 1,198 |
| Arts* |  | 0.4%* | 1.1% |  |  |  | 1,245 |
| Business and Economics* | 1.7% | 0.3% |  | 0.2% | 0.4% | 0.3% | 11,987 |
| Chemistry | 5.5% | 2.2% | 0.9% | 0.7%** | 0.7% |  | 1,651 |
| Education | 1.8% |  |  | 0.6%** | 0.5% | 0.1%** | 4,475 |
| Engineering and Technology | 9.7% | 3.7% | 1.5% | 1.1% | 0.7% | 0.6% | 14,140 |
| Ethics and Religion | 0.2%** | 0.5% | 0.5% |  |  |  | 4,354 |
| Geography | 2.5% |  |  | 0.4%* | 0.4%* |  | 1,367 |
| History | 0.8% | 0.8% | 0.5% |  |  |  | 8,798 |
| Languages and Literature | 0.4% | 0.7% | 0.4% | 0.1% |  |  | 9,798 |
| Law* | 0.8% | 0.7% | 0.1%* | 0.3% | 0.1%* | 0.2%** | 4,734 |
| Library Science and Bibliography | 0.7%* | 0.8%* |  |  | 0.5%* |  | 563 |
| Mathematics | 11.2% | 3.8% | 0.7% | 1.1% | 0.7% | 0.1%** | 4,093 |
| Medicine* | 5.5% | 0.8% | 0.3% | 0.7% | 1.1% | 0.6% | 12,227 |
| Military Science | 0.7%** |  |  |  |  |  | 822 |
| Music | 2.8% | 3.8% | 1.8% |  |  |  | 1,161 |
| Natural History and Biology | 2.5% | 0.3%* |  | 0.6%** | 0.7% |  | 1,640 |
| Philosophy | 0.3%* | 0.9% | 1.6% |  |  |  | 1,935 |
| Physics | 7.8% | 2.0% | 0.6% | 1.4% | 1.1% | 0.3%** | 2,628 |
| Physiology | 4.1% | 1.0% |  | 0.5%** | 0.8% |  | 1,716 |
| Political Sciences* |  | 0.6%* | 0.4% | 0.1%* |  |  | 5,098 |
| Psychology | 0.8% | 0.3%** |  | 0.3%* | 0.2%* | 0.4%** | 1,996 |
| Recreation and Leisure | 1.2%** | 2.5% |  | 0.7%* |  |  | 777 |
| Social Sciences |  | 0.2% | 0.1%** | 0.2% | 0.20% | 0.2% | 9,645 |
| Zoology | 0.5%* |  |  | 1.2%** |  |  | 758 |
| **Average of 26 Fields** | **2.8%** | **1.3%** | **0.7%** | **0.6%** | **0.6%** | **0.3%** | **110,603** |
| **Average of six* fields** | **2.1%** | **0.6%** | **0.5%** | **0.3%** | **0.5%** | **0.4%** | **36,493** |

Mendeley: Mendeley Readers; Twitter: Unique count of Twitter Users; Facebook: Unique count of Facebook Walls; Wikipedia: Wikipedia Articles; Blogs: Blog Posts; News: News Posts; All R^2^s are significant at p<0.001 except* p<0.05 and ** p<0.01.

Table 11. Adjusted R^2^ of Linear Squares regression of Total Library Holdings (TLH) with other metrics each as a sole independant variable.

| **Fields\Metrics** | **Scopus** | **GB** | **SM** | **GU** | **GR** | **GTR** | **GAR** | **# of Books** |
| --- | --- | --- | --- | --- | --- | --- | --- | --- |
| Agriculture | **10.3%** | 3.55 |  | 9.2% | 7.1% | 2.6% | 6.0% | 1,775 |
| Anthropology | 10.7% | 6.6% | 9.0% | 11.1% | **11.5%** | 7.6% | 7.0% | 1,198 |
| Arts | **15.8%** | 7.9% | 7.2% | 15.7% | 15.5% | 7.5% | 14.0% | 1,245 |
| Business and Economics | **15.1%** | 7.0% | 12.4% | 12.5% | 10.9% | 6.0% | 6.7% | 11,987 |
| Chemistry | 7.5% | 0.7%** |  | **8.3%** | 5.4% | 1.5% | 4.7% | 1,651 |
| Education | **15.7%** | 7.9% |  | 13.0% | 11.1% | 5.6% | 7.8% | 4,475 |
| Engineering and Technology | **14.2%** | 1.6% |  | 13.1% | 8.6% | 2.9% | 7.8% | 14,140 |
| Ethics and Religion | **14.8%** | 12.5% |  | 13.3% | 12.6% | 7.0% | 7.3% | 4,354 |
| Geography | **10.5%** | 5.1% |  | 9.0% | 8.4% | 4.0% | 6.4% | 1,367 |
| History | 13.3% | 8.5% |  | **14.7%** | 14.4% | 8.1% | 10.1% | 8,798 |
| Languages and Literature | 9.1% | 6.9% |  | **11.9%** | 10.7% | 5.4% | 7.0% | 9,798 |
| Law | **15.6%** | 9.4% | 10.5% | 15.5% | 12.2% | 5.6% | 10.3% | 4,734 |
| Library Science and Bibliography | **15.7%** | - |  | 14.1% | 12.3% | 8.0% | 7.7% | 563 |
| Mathematics | 12.4% | 2.9% |  | **16.9%** | 12.8% | 6.0% | 10.3% | 4,093 |
| Medicine | **11.5%** | 4.9% | 7.4% | 8.5% | 6.6% | 2.9% | 5.1% | 12,227 |
| Military Science | 8.1% | 6.8% |  | **8.6%** | 8.1% | 5.3% | 6.6% | 822 |
| Music | 14.2% | 7.1% |  | **14.7%** | 14.6% | 6.1% | 11.6% | 1,161 |
| Natural History and Biology | 13.2% | 6.5% |  | **16.4%** | 14.0% | 6.8% | 12.6% | 1,640 |
| Philosophy | 11.6% | 8.5% |  | **13.1%** | 12.5% | 6.5% | 9.4% | 1,935 |
| Physics | 11.1% | 4.4% |  | **11.6%** | 8.6% | 4.3% | 7.9% | 2,628 |
| Physiology | **11.6%** | 8.3% |  | 9.8% | 8.0% | 3.3% | 6.7% | 1,716 |
| Political Sciences | **16.8%** | 10.0% | 15.0% | 13.9% | 13.1% | 6.9% | 9.6% | 5,098 |
| Psychology | **19.9%** | 11.4% |  | 14.1% | 14.4% | 9.8% | 8.2% | 1,996 |
| Recreation and Leisure | 8.9% | 4.9% |  | 17.0% | **17.1%** | 10.8% | 9.6% | 777 |
| Social Sciences | **15.3%** | 7.0% |  | 14.8% | 14.2% | 7.9% | 8.8% | 9,645 |
| Zoology | 9.6% | 4.0% |  | **16.8%** | 14.6% | 7.1% | 11.5% | 758 |
| **Total** | 12.5% | 6.8% | **10.6%** | 14.6% | 12.4% | 6.5% | 9.5% | 110,603 |

** significant at p<0.01; Red fonts show R^2^ estimates stronger in TLH than LPH.

Continuation for Table 11. Adjusted R^2^ of Linear Squares regression of Total Library Holdings (TLH) with other metrics each as a sole independant variable.

| **Fields\Metrics** | **Mendeley** | **Twitter** | **Facebook** | **Wikipedia** | **Blogs** | **News** | **# of Books** |
| --- | --- | --- | --- | --- | --- | --- | --- |
| Agriculture | 1.3% | 0.3%* |  | 0.7% | 0.5%** | 0.3%* | 1,775 |
| Anthropology* |  | 1.8% | 1% |  | 0.4%* | 0.5%** | 1,198 |
| Arts* | 0.3%* | 1% | 2.5% | 0.2%* |  |  | 1,245 |
| Business and Economics* | 0.9% |  | 0.1%** | 0.3% | 1% | 0.6% | 11,987 |
| Chemistry | 4.2% | 0.7%** |  | 1.1% | 0.9% |  | 1,651 |
| Education | 0.7% | 0.4% | 0.1%** | 0.7% | 1% | 0.1%** | 4,475 |
| Engineering and Technology | 5.6% | 1.5% | 0.3% | 1.1% | 0.9% | 0.7% | 14,140 |
| Ethics and Religion |  | 0.6% | 0.9% | 0.1%* | 0.2%** |  | 4,354 |
| Geography | 0.9% | 0.3%* |  | 0.2%* | 0.5%** |  | 1,367 |
| History | 0.2% | 0.8%* | 0.9% | 0.1%** | 0.1% | 0.1%* | 8,798 |
| Languages and Literature | 0.4% | 2.2% | 1.2% | 0.2% |  |  | 9,798 |
| Law* | 1.1% | 0.7% | 0.1%** | 0.4% |  | 0.3% | 4,734 |
| Library Science and Bibliography | 1.6%** |  |  |  | 0.9%* |  | 563 |
| Mathematics | 6.1% | 1.6% |  | 1.3% | 1.2% | 0.1%* | 4,093 |
| Medicine* | 3.6% | 0.2% | 0.0* | 0.9% | 1.6% | 0.7% | 12,227 |
| Military Science | 0.9%** |  |  |  |  |  | 822 |
| Music | 0.4%* | 5.2% | 2.6% |  |  |  | 1,161 |
| Natural History and Biology | 0.9% |  | 0.7% | 0.5%** | 1.1% |  | 1,640 |
| Philosophy |  | 1.2% | 2.2% |  | 0.2%* |  | 1,935 |
| Physics | 4.7% | 0.4%** |  | 1.7% | 1.2% | 0.1%* | 2,628 |
| Physiology | 3.8% | 0.3%* |  | 0.8% | 1.5% | 0.3%* | 1,716 |
| Political Sciences* |  | 0.9% | 0.7% | 0.2%** | 0.1%** | 0.3% | 5,098 |
| Psychology | 1.3% |  | 0.2%* | 0.8% | 0.8% | 0.5%** | 1,996 |
| Recreation and Leisure | 1.8% | 8.0% | 1.2%** | 0.9%** |  |  | 777 |
| Social Sciences |  | 0.8% | 0.4% | 0.3% | 0.6% | 0.3% | 9,645 |
| Zoology |  |  |  | 1.0%** | 0.9%** |  | 758 |
| **Average of 26 fields** | **2.0%** | **1.4%** | **0.9%** | **0.6%** | **0.8%** | **0.4%** | **110,603** |
| **Average of six* fields** | **1.5%** | **0.9%** | **0.7%** | **0.4%** | **0.8%** | **0.5%** | **36,493** |

Mendeley: Mendeley Readers; Twitter: Unique count of Twitter Users; Facebook: Unique count of Facebook Walls; Wikipedia: Wikipedia Articles; Blogs: Blog Posts; News: News Posts; All R^2^s are significant at p<0.001 except* p<0.05 and ** p<0.01.

**Regression Analyses: LPH and LEH Combined Predictor Models across 26 Fields**

Table 12. Statistically significant linear regression standardized coefficients (β) in 26 fields. The dependent metric is Scopus Citations and predictors are LPH and LEH.

| Subject Fields | Regression Coefficients (Beta) | | Adjusted R^2^ |
| --- | --- | --- | --- |
|  | LPH | LEH |  |
| Agriculture | **0.433** | 0.145 | 22.80% |
| Anthropology | **0.397** | 0.095 | 18% |
| Arts | **0.428** | 0.166 | 23.60% |
| Business and Economics | **0.493** | 0.132 | 28% |
| Chemistry | **0.472** | 0.231 | 25.20% |
| Education | **0.378** | 0.233 | 22.70% |
| Engineering and Technology | **0.376** | 0.356 | 23.60% |
| Ethics and Religion | **0.485** | 0.095 | 26.80% |
| Geography | **0.445** | 0.064 | 20.90% |
| History | **0.408** | 0.105 | 20.40% |
| Languages and Literature | **0.404** | 0.061 | 17.80% |
| Law | **0.484** | 0.099 | 27.40% |
| Library Science and Bibliography | **0.388** | 0.253 | 22.80% |
| Mathematics | **0.387** | 0.254 | 21.00% |
| Medicine | **0.465** | 0.145 | 25.80% |
| Military Science | **0.508** |  | 27.20% |
| Music | **0.443** | 0.103 | 23.20% |
| Natural History and Biology | **0.519** | 0.057** | 28.40% |
| Philosophy | **0.480** | 0.046* | 24.40% |
| Physics | **0.379** | 0.290 | 22.90% |
| Physiology | **0.545** | 0.078 | 32.10% |
| Political Sciences | **0.498** | 0.102 | 29.40% |
| Psychology | **0.443** | 0.194 | 27.80% |
| Recreation and Leisure | **0.432** |  | 19.80% |
| Social Sciences | **0.482** | 0.119 | 27.60% |
| Zoology | **0.506** |  | 24.80% |

All R^2^s are significant at P<0.001; All coefficients are significant at p<0.001 except *p<0.05; ** p<0.01.

Table 13. Statistically significant linear regression standardized coefficients (β) in 26 fields. The dependent metric is Google Books Citations and predictors are LPH and LEH.

| Subject Fields | Regression Coefficients (Beta) | | Adjusted R^2^ |
| --- | --- | --- | --- |
|  | LPH | LEH |  |
| Agriculture | **0.449** | -0.046* | 19.6% |
| Anthropology | **0.416** |  | 16.7% |
| Arts | **0.460** |  | 21.0% |
| Business and Economics | **0.504** | -0.054 | 24.8% |
| Chemistry | **0.415** | -0.044* | 17.7% |
| Education | **0.334** | 0.136 | 14.5% |
| Engineering and Technology | **0.365** |  | 13.2% |
| Ethics and Religion | **0.435** | 0.054 | 20.5% |
| Geography | **0.448** | -0.068** | 19.5% |
| History | **0.449** | -0.039 | 19.2% |
| Languages and Literature | **0.455** | -0.067 | 19.7% |
| Law | **0.493** |  | 24.1% |
| Library Science and Bibliography | **0.266** | -0.138** | 8.0% |
| Mathematics | **0.371** | 0.035* | 13.8% |
| Medicine | **0.452** |  | 20.2% |
| Military Science | **0.611** | -0.057* | 36.0% |
| Music | **0.480** | -0.062* | 21.5% |
| Natural History and Biology | **0.468** |  | 21.3% |
| Philosophy | **0.419** |  | 17.8% |
| Physics | **0.409** | 0.087 | 17.5% |
| Physiology | **0.516** |  | 26.6% |
| Political Sciences | **0.476** |  | 22.3% |
| Psychology | **0.451** |  | 20.0% |
| Recreation and Leisure | **0.443** | -0.082* | 18.2% |
| Social Sciences | **0.499** | -0.074 | 23.5% |
| Zoology | **0.376** | -0.075* | 13.2% |

All R^2^s are significant at P<0.001; All coefficients are significant at p<0.001 except *p<0.05; ** p<0.01.

Table 14. Statistically significant linear regression standardized coefficients (β) in six sample fields. The dependent metric is Syllabus Mentions (of opensyllabus.org) and predictors are LPH and LEH.

| Subject Fields | Regression Coefficients (Beta) | | Adjusted R^2^ |
| --- | --- | --- | --- |
|  | LPH | LEH |  |
| Anthropology | **.502** |  | 25.1% |
| Arts | **.460** |  | 21.1% |
| Business and Economics | **.469** | .085 | 23.9% |
| Law | **.428** | .049 | 19.8% |
| Medicine | **.518** | -.032 | 26.4% |
| Political Sciences | **.501** | .042** | 26.7% |

All R^2^s are significant at P<0.001; All coefficients are significant at p<0.001 except ** p<0.01.

Table 15. Statistically significant linear regression standardized coefficients (β) in 26 fields. The dependent metric is Syllabus Mentions (of altmetric.com) and predictors are LPH and LEH.

| Subject Fields | Regression Coefficients (Beta) | | Adjusted R^2^ |
| --- | --- | --- | --- |
|  | LPH | LEH |  |
| Agriculture | **0.168** |  | 3.2% |
| Anthropology | **0.253** |  | 6.3% |
| Arts | **0.219** |  | 5.1% |
| Business and Economics | **0.229** | 0.055 | 5.9% |
| Chemistry | **0.179** | 0.071** | 3.3% |
| Education | **0.194** | 0.054 | 4.3% |
| Engineering and Technology | **0.160** | 0.093 | 3.1% |
| Ethics and Religion | **0.236** | 0.038* | 6.2% |
| Geography | **0.186** | 0.090** | 4.6% |
| History | **0.276** |  | 7.4% |
| Languages and Literature | **0.267** | -0.036 | 6.8% |
| Law | **0.228** | 0.041** | 5.9% |
| Library Science and Bibliography | **0.297** | 0.088 | 9.7% |
| Mathematics | **0.228** | 0.111* | 6.3% |
| Medicine | **0.188** | 0.024** | 3.7% |
| Military Science | **0.250** |  | 5.7% |
| Music | **0.254** |  | 6.3% |
| Natural History and Biology | **0.276** |  | 7.9% |
| Philosophy | **0.225** |  | 5.1% |
| Physics | **0.242** | 0.089 | 6.6% |
| Physiology | **0.249** |  | 6.1% |
| Political Sciences | **0.239** | 0.050** | 6.7% |
| Psychology | **0.201** | 0.083 | 5.5% |
| Recreation and Leisure | **0.235** | 0.091* | 7.2% |
| Social Sciences | **0.250** | 0.045 | 7.0% |
| Zoology | **0.239** |  | 5.9% |

All R^2^s are significant at P<0.001; All coefficients are significant at p<0.001 except *p<0.05; ** p<0.01.

Table 16. Statistically significant linear regression standardized coefficients (β) in 26 fields. The dependent metric is Goodreads Users (GU) and predictors are LPH and LEH.

| Subject Fields | Regression Coefficients (Beta) | | Adjusted R^2^ |
| --- | --- | --- | --- |
|  | LPH | LEH |  |
| Agriculture | **0.374** | 0.156 | 18.2% |
| Anthropology | **0.374** | 0.154 | 18.4% |
| Arts | **0.383** | 0.169 | 19.8% |
| Business and Economics | **0.347** | 0.210 | 18.6% |
| Chemistry | **0.332** | 0.241 | 15.0% |
| Education | **0.379** | 0.213 | 21.6% |
| Engineering and Technology | 0.299 | **0.365** | 19.6% |
| Ethics and Religion | **0.437** | 0.098 | 22.4% |
| Geography | **0.350** | 0.145 | 15.7% |
| History | **0.388** | 0.130 | 20.0% |
| Languages and Literature | **0.339** | 0.161 | 16.6% |
| Law | **0.445** | 0.115 | 24.4% |
| Library Science and Bibliography | **0.375** | 0.269 | 22.7% |
| Mathematics | 0.318 | **0.354** | 22.1% |
| Medicine | **0.477** | 0.048 | 23.6% |
| Military Science | **0.460** | 0.100** | 24.0% |
| Music | **0.403** | 0.147 | 21.7% |
| Natural History and Biology | **0.460** | 0.187 | 28.0% |
| Philosophy | **0.412** | 0.118 | 21.0% |
| Physics | **0.392** | 0.260 | 22.2% |
| Physiology | **0.502** |  | 25.7% |
| Political Sciences | **0.394** | 0.120 | 20.3% |
| Psychology | **0.362** | 0.169 | 19.0% |
| Recreation and Leisure | **0.293** | 0.272 | 20.0% |
| Social Sciences | **0.405** | 0.175 | 23.1% |
| Zoology | **0.476** | 0.193 | 30.5% |

All R^2^s are significant at P<0.001; All coefficients are significant at p<0.001 except *p<0.05; ** p<0.01.

Table 17. Statistically significant linear regression standardized coefficients (β) in 26 fields. The dependent metric is Goodreads Ratings (GR) and predictors are LPH and LEH.

| Subject Fields | Regression Coefficients (Beta) | | Adjusted R^2^ |
| --- | --- | --- | --- |
|  | LPH | LEH |  |
| Agriculture | **0.343** | 0.123 | 14.5% |
| Anthropology | **0.400** | 0.130 | 19.6% |
| Arts | **0.407** | 0.134 | 20.3% |
| Business and Economics | **0.330** | 0.181 | 15.9% |
| Chemistry | **0.301** | 0.156 | 10.4% |
| Education | **0.385** | 0.165 | 19.7% |
| Engineering and Technology | **0.290** | 0.267 | 13.7% |
| Ethics and Religion | **0.452** | 0.071 | 22.7% |
| Geography | **0.353** | 0.109 | 14.6% |
| History | **0.405** | 0.105 | 20.2% |
| Languages and Literature | **0.342** | 0.129 | 15.4% |
| Law | **0.394** | 0.096 | 18.9% |
| Library Science and Bibliography | **0.379** | 0.227 | 20.6% |
| Mathematics | **0.304** | 0.284 | 16.9% |
| Medicine | **0.434** |  | 19.1% |
| Military Science | **0.416** | 0.101** | 20.0% |
| Music | **0.412** | 0.137 | 22.0% |
| Natural History and Biology | **0.428** | 0.153 | 23.2% |
| Philosophy | **0.412** | 0.104 | 20.4% |
| Physics | **0.396** | 0.187 | 19.3% |
| Physiology | **0.468** |  | 21.8% |
| Political Sciences | **0.382** | 0.112 | 18.8% |
| Psychology | **0.379** | 0.159 | 20.0% |
| Recreation and Leisure | **0.317** | 0.248 | 20.1% |
| Social Sciences | **0.404** | 0.155 | 21.9% |
| Zoology | **0.467** | 0.158 | 27.5% |

All R^2^s are significant at P<0.001; All coefficients are significant at p<0.001 except *p<0.05; ** p<0.01.

Table 18. Statistically significant linear regression standardized coefficients (β) in 26 fields. The dependent metric is Goodreads Text Reviews (GTR) and predictors are LPH and LEH.

| Subject Fields | Regression Coefficients (Beta) | | Adjusted R^2^ |
| --- | --- | --- | --- |
|  | LPH | LEH |  |
| Agriculture | **0.213** | 0.065** | 5.3% |
| Anthropology | **0.338** | 0.091** | 13.3% |
| Arts | **0.321** | 0.071** | 11.5% |
| Business and Economics | **0.256** | 0.118 | 8.9% |
| Chemistry | **0.154** | 0.082** | 2.7% |
| Education | **0.299** | 0.092 | 10.7% |
| Engineering and Technology | **0.187** | 0.139 | 4.8% |
| Ethics and Religion | **0.373** |  | 14.5% |
| Geography | **0.244** | 0.059* | 6.6% |
| History | **0.336** | 0.050 | 12.6% |
| Languages and Literature | **0.241** | 0.091 | 7.6% |
| Law | **0.275** | 0.060 | 9.0% |
| Library Science and Bibliography | **0.304** | 0.181 | 13.1% |
| Mathematics | **0.222** | 0.176 | 7.9% |
| Medicine | **0.264** | 0.028** | 7.3% |
| Military Science | **0.335** | 0.079** | 12.8% |
| Music | **0.268** | 0.091** | 9.3% |
| Natural History and Biology | **0.297** | 0.110 | 11.2% |
| Philosophy | **0.333** | 0.051* | 12.2% |
| Physics | **0.292** | 0.121 | 10.0% |
| Physiology | **0.290** |  | 8.5% |
| Political Sciences | **0.285** | 0.070 | 10.0% |
| Psychology | **0.312** | 0.117 | 12.9% |
| Recreation and Leisure | 0.202 | **0.222** | 11.2% |
| Social Sciences | **0.293** | 0.119 | 11.8% |
| Zoology | **0.326** | 0.114 | 13.4% |

All R^2^s are significant at P<0.001; All coefficients are significant at p<0.001 except *p<0.05; ** p<0.01.

Table 19. Statistically significant linear regression standardized coefficients (β) in 26 fields. The dependent metric is Goodreads Average Ratings (GAR) and predictors are LPH and LEH.

| Subject Fields | Regression Coefficients (Beta) | | Adjusted R^2^ |
| --- | --- | --- | --- |
|  | LPH | LEH |  |
| Agriculture | **0.303** | 0.136 | 12.3% |
| Anthropology | **0.264** | 0.153 | 10.7% |
| Arts | **0.279** | 0.165 | 13.4% |
| Business and Economics | **0.248** | 0.167 | 10.1% |
| Chemistry | **0.266** | 0.173 | 8.9% |
| Education | **0.269** | 0.188 | 12.5% |
| Engineering and Technology | 0.235 | **0.288** | 12.2% |
| Ethics and Religion | **0.280** | 0.104 | 10.5% |
| Geography | **0.300** | 0.123 | 11.5% |
| History | **0.255** | 0.160 | 11.7% |
| Languages and Literature | **0.251** | 0.132 | 9.6% |
| Law | **0.346** | 0.109 | 15.5% |
| Library Science and Bibliography | 0.207 | **0.251** | 11.1% |
| Mathematics | 0.223 | **0.305** | 14.0% |
| Medicine | **0.381** | 0.041 | 15.2% |
| Military Science | **0.355** | 0.121 | 15.8% |
| Music | **0.309** | 0.168 | 15.3% |
| Natural History and Biology | **0.381** | 0.175 | 20.1% |
| Philosophy | **0.292** | 0.142 | 12.8% |
| Physics | **0.348** | 0.211 | 16.6% |
| Physiology | **0.421** |  | 18.2% |
| Political Sciences | **0.294** | 0.130 | 13.0% |
| Psychology | **0.251** | 0.166 | 11.1% |
| Recreation and Leisure | **0.226** | 0.214 | 12.0% |
| Social Sciences | **0.312** | 0.144 | 14.1% |
| Zoology | **0.413** | 0.148 | 21.9% |

All R^2^s are significant at P<0.001; All coefficients are significant at p<0.001 except *p<0.05; ** p<0.01.

Table 20. Statistically significant linear regression standardized coefficients (β) in 26 fields. The dependent metric is Mendeley Readers and predictors are LPH and LEH.

| Subject Fields | Regression Coefficients (Beta) | | Adjusted R^2^ |
| --- | --- | --- | --- |
|  | LPH | LEH |  |
| Agriculture | -0.113 | **0.205** | 4.6% |
| Anthropology |  | **-0.068*** | 0.4%* |
| Arts | **-0.066*** |  | 0.3%* |
| Business and Economics | -0.032 | **0.134** | 1.7% |
| Chemistry | 0.102 | **0.248** | 6.5% |
| Education | -0.053 | **0.143** | 2.0% |
| Engineering and Technology |  | **0.311** | 9.7% |
| Ethics and Religion | **0.148** | -0.091 | 2.3% |
| Geography |  | **0.163** | 2.5% |
| History | 0.092 | **-0.122** | 1.6% |
| Languages and Literature | 0.030** | **-0.070** | 0.5% |
| Law | **0.103** | 0.059 | 1.7% |
| Library Science and Bibliography | **-0.110**** | -0.085* | 1.7%** |
| Mathematics |  | **0.335** | 11.2% |
| Medicine |  | **0.232** | 5.5% |
| Military Science |  | **0.081*** | 0.8%* |
| Music | **0.183** | **-0.222** | 5.7% |
| Natural History and Biology |  | **0.163** | 2.5% |
| Philosophy | **0.122** | -0.093 | 1.6% |
| Physics | 0.085 | **0.279** | 8.5% |
| Physiology | 0.099 | **0.183** | 5.0% |
| Political Sciences | **0.068** | -0.037** | 0.4% |
| Psychology | **0.083** | 0.068** | 1.3% |
| Recreation and Leisure |  | **-0.116**** | 1.1%** |
| Social Sciences | **0.036**** |  | 0.1%** |
| Zoology |  | **0.094** | 0.8%* |

All R^2^s and coefficients are significant at p <0.001, except *p<0.05; ** p<0.01.

Table 21. Statistically significant linear regression standardized coefficients (β) in 26 fields. The dependent metric is Twitter Users and predictors are LPH and LEH.

| Subject Fields | Regression Coefficients (Beta) | | Adjusted R^2^ |
| --- | --- | --- | --- |
|  | LPH | LEH |  |
| Agriculture | -0.125 | **0.138** | 2.8% |
| Anthropology |  | **-0.084**** | 0.8%** |
| Arts | **-0.068*** |  | 0.8%** |
| Business and Economics | -0.047 | **0.058** | 0.5% |
| Chemistry | -0.051* | **0.144** | 2.4% |
| Education | **-0.108** | 0.045** | 1.2% |
| Engineering and Technology | -0.051 | **0.187** | 4.0% |
| Ethics and Religion |  | **-0.073** | 0.5% |
| Geography | **-0.088**** |  | 0.7%** |
| History | 0.027* | **-0.096** | 0.8% |
| Languages and Literature | **-0.085** | -0.066 | 1.4% |
| Law | 0.030* | **-0.092** | 0.7% |
| Library Science and Bibliography | **-0.167** | 0.112** | 3.4% |
| Mathematics |  | **0.194** | 3.8% |
| Medicine | -0.028** | **0.093** | 0.8% |
| Military Science |  |  |  |
| Music |  | **-0.108** | 4.0% |
| Natural History and Biology | **-0.087**** | 0.075** | 1.0% |
| Philosophy |  | **-0.092** | 0.8% |
| Physics |  | **0.143** | 2.0% |
| Physiology |  | **0.102** | 1.0% |
| Political Sciences |  | **-0.077** | 0.6% |
| Psychology | -0.065** | **-0.077**** | 0.6%** |
| Recreation and Leisure | **-0.192** | -0.110** | 5.8% |
| Social Sciences | **-0.047** | -0.032** | 0.4% |
| Zoology | **-0.083*** |  |  |

All R^2^s and coefficients are significant at p <0.001, except *p<0.05; ** p<0.01.

Table 22. Statistically significant linear regression standardized coefficients (β) in 26 fields. The dependent metric is Facebook Walls and predictors are LPH and LEH.

| Subject Fields | Regression Coefficients (Beta) | | Adjusted R^2^ |
| --- | --- | --- | --- |
|  | LPH | LEH |  |
| Agriculture | **-0.123** | 0.104 | 2.1% |
| Anthropology | **-0.100**** |  | 1.1% |
| Arts | **-0.104** | -0.090** | 2.1% |
| Business and Economics | **-0.085** | 0.030** | 0.7% |
| Chemistry | -0.054* | **0.092** | 1.1% |
| Education | **-0.116** | 0.043** | 1.3% |
| Engineering and Technology | -0.085 | **0.113** | 2.2% |
| Ethics and Religion | -0.048** | **-0.060** | 0.7% |
| Geography | **-0.110** |  | 1.1% |
| History | -0.036** | **-0.059** | 0.6% |
| Languages and Literature | **-0.077** | -0.045 | 0.9% |
| Law |  | **-0.031*** |  |
| Library Science and Bibliography | **-0.199** |  | 3.9% |
| Mathematics | **-0.680** | 0.085 | 1.2% |
| Medicine | -0.053 | **0.066** | 0.6% |
| Military Science |  |  |  |
| Music |  | **-0.120** | 2.0% |
| Natural History and Biology |  | **-0.135** | 1.6% |
| Philosophy | -0.047* | **-0.144** | 1.7% |
| Physics | -0.044* | **0.078** | 0.7% |
| Physiology |  |  |  |
| Political Sciences | -0.043** | **-0.050**** | 0.5% |
| Psychology | **-0.110** |  | 1.0% |
| Recreation and Leisure | **-0.098**** |  | 1.0%** |
| Social Sciences | **-0.067** |  | 0.5% |
| Zoology | **-0.102**** |  | 0.7%* |

All R^2^s and coefficients are significant at p <0.001, except *p<0.05; ** p<0.01.

Table 23. Statistically significant linear regression standardized coefficients (β) in 26 fields. The dependent metric is Wikipedia Articles and predictors are LPH and LEH.

| Subject Fields | Regression Coefficients (Beta) | | Adjusted R^2^ |
| --- | --- | --- | --- |
|  | LPH | LEH |  |
| Agriculture | 0.056* | **0.080**** | 1.0% |
| Anthropology |  |  |  |
| Arts |  |  |  |
| Business and Economics | 0.035 | **0.038** | 0.3% |
| Chemistry | **0.109** | 0.097 | 1.8% |
| Education | 0.047 | **0.070** | 0.8% |
| Engineering and Technology | 0.058 | **0.111** | 1.4% |
| Ethics and Religion | **0.086** | -0.033* | 0.6% |
| Geography |  | **0.064*** | 0.3%* |
| History | **0.075** | -0.026* | 0.5% |
| Languages and Literature | 0.026* | **0.030**** | 0.2% |
| Law | **0.051**** | 0.036* | 0.5% |
| Library Science and Bibliography |  |  |  |
| Mathematics | 0.088 | **0.108** | 1.8% |
| Medicine | 0.073 | 0.070 | 1.2% |
| Military Science | **0.091*** |  | 0.5%* |
| Music | **0.068*** |  |  |
| Natural History and Biology | 0.050* | **0.068**** | 0.7%** |
| Philosophy | **0.060*** |  | 0.3%* |
| Physics | 0.079 | **0.118** | 2.0% |
| Physiology | **0.097** | 0.057 | 1.4% |
| Political Sciences | **0.052** |  | 0.3% |
| Psychology | **0.060*** |  | 0.5% |
| Recreation and Leisure |  | **0.078*** | 0.8%* |
| Social Sciences | **0.060** | 0.032** | 0.5% |
| Zoology |  | **0.107**** | 1.2% |

All R^2^s and coefficients are significant at p <0.001, except *p<0.05; ** p<0.01.

Table 24. Statistically significant linear regression standardized coefficients (β) in 26 fields. The dependent metric is Blog Pages and predictors are LPH and LEH.

| Subject Fields | Regression Coefficients (Beta) | | Adjusted R^2^ |
| --- | --- | --- | --- |
|  | LPH | LEH |  |
| Agriculture |  | **0.054*** | 0.4%* |
| Anthropology | **0.169** |  | 2.6% |
| Arts | **0.070*** |  | 0.4%* |
| Business and Economics | **0.138** | 0.041 | 2.2% |
| Chemistry | **0.107** | 0.098 | 1.8% |
| Education | **0.085** | 0.056 | 1.2% |
| Engineering and Technology | 0.072 | **0.093** | 1.2% |
| Ethics and Religion | **0.120** | -0.031* | 1.3% |
| Geography | **0.080**** | 0.055* | 0.9%** |
| History | **0.092** |  | 0.7% |
| Languages and Literature | **0.026*** |  | 0.0%* |
| Law | **0.121** | -0.070 | 1.4% |
| Library Science and Bibliography |  |  | 0.8%* |
| Mathematics | **0.109** | 0.085 | 1.8% |
| Medicine | **0.115** | 0.086 | 2.4% |
| Military Science | **0.129** |  | 1.4% |
| Music |  |  |  |
| Natural History and Biology | 0.113 | 0.066** | 1.9% |
| Philosophy | **0.110** |  | 1.1% |
| Physics | 0.099 | **0.106** | 2.1% |
| Physiology | **0.162** | 0.057* | 3.2% |
| Political Sciences | **0.139** | -0.053 | 1.6% |
| Psychology | **0.120** |  | 1.5% |
| Recreation and Leisure | **0.089*** |  | 0.5% |
| Social Sciences | **0.121** |  | 1.5% |
| Zoology | **0.144** |  | 2.2% |

All R^2^s and coefficients are significant at p <0.001, except *p<0.05; ** p<0.01.

Table 25. Statistically significant linear regression standardized coefficients (β) in 26 fields. The dependent metric is News Posts and predictors are LPH and LEH.

| Subject Fields | Regression Coefficients (Beta) | | Adjusted R^2^ |
| --- | --- | --- | --- |
|  | LPH | LEH |  |
| Agriculture |  | **0.054** | 0.3%* |
| Anthropology | **0.13** |  | 1.5% |
| Arts |  |  |  |
| Business and Economics | **0.071** | 0.042 | 0.8% |
| Chemistry |  |  |  |
| Education | **0.045**** | 0.031* | 0.3% |
| Engineering and Technology | 0.044 | **0.083** | 0.8% |
| Ethics and Religion | **0.101** | -0.035* | 0.9% |
| Geography | **0.092** | -0.040 | 0.8% |
| History | **0.036** |  | 0.1%** |
| Languages and Literature |  |  |  |
| Law | **0.059** |  | 0.4% |
| Library Science and Bibliography |  |  |  |
| Mathematics |  | **0.041**** | 0.1%* |
| Medicine | 0.061 | **0.069** | 1.0% |
| Military Science |  |  |  |
| Music |  |  |  |
| Natural History and Biology |  |  |  |
| Philosophy |  |  |  |
| Physics |  | **0.056**** | 0.3%** |
| Physiology | **0.076**** |  | 0.6%** |
| Political Sciences | **0.116** |  | 1.2% |
| Psychology | **0.060** | 0.049 | 0.7%** |
| Recreation and Leisure |  |  |  |
| Social Sciences | 0.022* | **0.076** | 0.7% |
| Zoology |  |  |  |

All R^2^s and coefficients are significant at p <0.001, except *p<0.05; ** p<0.01.

### Regression Analyses: Multi-variate Models across 26 Fields

Table 26. Statistically significant linear regression standardized coefficients (β) in 26 fields. The dependent metric is Total Library Holding. Only Goodreads Ratings is excluded from models due to causing collinearity.

| Fields | Scopus | GB | GU | GTR | GAR | Mendeley | Twitter | Facebook | Wiki | Blogs | News | R^2^ |
| --- | --- | --- | --- | --- | --- | --- | --- | --- | --- | --- | --- | --- |
| Agriculture | .225 | .072^**^ | **.241** |  |  |  |  |  |  |  |  | 16.20% |
| Anthropology | **.205** | .18 | .186^**^ |  |  | -.088^**^ | -.131 |  |  |  |  | 21.70% |
| Arts | **.239** | .122 | .127^*^ |  | .172 | -.092^**^ |  | -.079^**^ | .050^*^ |  |  | 27.90% |
| Business and Economics | .232 | .123 | **.252** |  |  | .027^**^ | -.051 |  |  |  |  | 21.40% |
| Chemistry | **.211** | -.065^*^ | .193 |  |  | .065^*^ |  |  |  |  |  | 12.60% |
| Education | .25 | .154 | **.275** |  |  |  | .030^*^ |  |  |  |  | 24.70% |
| Engineering and Technology | **.266** |  | .256 |  |  | .087 | .038 |  |  |  |  | 21.60% |
| Ethics and Religion | .222 | .212 | **.279** |  |  | -.09 | -.067 | -.035^*^ |  |  |  | 26.00% |
| Geography | **.211** | .113 | .194 |  |  |  | -.109^**^ |  |  |  |  | 18.10% |
| History | .228 | .154 | **.232** | .029^*^ | .063 | -.096 | -.091 | -.022 |  |  |  | 25.90% |
| Languages and Literature | .175 | .154 | **.268** |  |  | -.099 | -.102 | -.039 | .046 |  |  | 21.90% |
| Law | .21 | .146 | **.265** |  |  |  | -.128 |  | .028^*^ | -.046^**^ | .034^*^ | 25.90% |
| Library Science and Bibliography | **.326** | -.093^*^ | .229^**^ |  |  | -.185 |  |  |  |  |  | 24.00% |
| Mathematics | .215 | .037^*^ | **.354** | -.042^*^ |  | .055^**^ |  |  |  |  |  | 22.90% |
| Medicine | **.203** | .064 | .197 |  |  | .12 | -.044 |  |  | .035 |  | 16.90% |
| Military Science | .114^**^ | **.159** |  |  |  | .076^*^ |  |  |  |  |  | 14.10% |
| Music | .237 | .124 | **.249** |  |  | -.119 | -.19 |  | .052^*^ |  |  | 29.10% |
| Natural History and Biology | .202 | .091 | **.256** |  | .079^*^ |  |  |  |  |  |  | 23.80% |
| Philosophy | .171 | .154 | **.216** |  |  | -.070^**^ | -.069^**^ | -.097 |  |  |  | 22.40% |
| Physics | .209 | .045^*^ | **.231** |  |  | .064^**^ |  |  | .060^**^ |  |  | 18.10% |
| Physiology | **.18** | .116 | .161 |  |  | .081^**^ |  |  |  |  |  | 17.10% |
| Political Sciences | **.268** | .152 | .213 | .034^*^ | .048^*^ | -.065 | -.099 |  |  | -.036^*^ | .027^*^ | 27.10% |
| Psychology | **.291** | .178 | .129^**^ | .116 |  |  |  |  |  | -.044^*^ |  | 27.70% |
| Recreation and Leisure | .144 | .099^**^ | **.281** |  |  | -.083^*^ | -.245 |  | .092^**^ |  |  | 29.30% |
| Social Sciences | .245 | .133 | **.273** | .028^*^ |  | -.056 | -.113 |  | .036 |  |  | 25.30% |
| Zoology | .214 |  | **.367** |  |  |  |  |  | .080^*^ |  |  | 23.10% |
| **Average** | **.219** | **.109** | **.237** | **.033** | **.091** | **-.025** | **-.091** | **.056** | **-.054** | **.031** | **-.023** | **22.5%** |

All R^2^s are significant at p<0.001; All coefficients are significant at p<0.001 except *p<0.05; ** p<0.01.

Table 27. Statistically significant linear regression standardized coefficients (β) in 26 fields. The dependent metric is Library Print Holding. Only Goodreads Ratings is excluded from models due to causing collinearity.

| Fields | Scopus | GB | GU | GTR | GAR | Mendeley | Twitter | Facebook | Wiki | Blogs | News | R^2^ | R^2^ LPH/TLH |
| --- | --- | --- | --- | --- | --- | --- | --- | --- | --- | --- | --- | --- | --- |
| Agriculture | .302 | **.318** | .292 |  |  | -.204 |  |  |  | .050^*^ |  | 42.4% | 2.6 |
| Anthropology | .228 | **.316** | .229 | .101^**^ |  | -.070^**^ |  | -.059^*^ |  |  |  | 35.7% | 1.6 |
| Arts | .228 | **.315** | .200 | .099^**^ |  | -.108 |  |  |  |  |  | 38% | 1.4 |
| Business and Economics | .284 | **.336** | .219 |  |  | -.103 | -.043 | -.038 |  | .045 |  | 41.3% | 1.9 |
| Chemistry | **.294** | .252 | .165 |  | .057^*^ | -.138 | -.074^**^ |  |  |  |  | 30.4% | 2.4 |
| Education | .256 | .239 | **.300** | .045^**^ |  | -.111 | -.077 | -.058 |  | .038^**^ |  | 34.1% | 1.4 |
| Engineering and Technology | .223 | **.260** | .148 | .039 |  | -.158 | -.052 | -.061 | .020^*^ | .041 |  | 23.6% | 1.1 |
| Ethics and Religion | .286 | .263 | **.291** | .067 | -.056^**^ |  | -.054 |  | .029^*^ |  |  | 40.9% | 1.6 |
| Geography | .293 | **.305** | .193 | .061^*^ |  | -.084 |  | -.052^*^ |  | .078^**^ |  | 38% | 2.1 |
| History | .228 | **.291** | .256 | .088 |  | -.033^**^ |  |  |  |  |  | 36.4% | 1.4 |
| Languages and Literature | .231 | **.314** | .239 | .054 |  | -.054 | -.055 | -.032 | .017^*^ |  | .019^*^ | 34.6% | 1.6 |
| Law | .259 | **.312** | .297 |  |  | -.045 | -.042^**^ |  |  | .029^*^ |  | 43.8% | 1.7 |
| Library Science and Bibliography | .236 | .139 | **.328** |  |  | -.137 | -.103^*^ | -.120^**^ |  |  |  | 30.1% | 1.3 |
| Mathematics | .263 | .237 | **.304** |  | -.049^*^ | -.173 | -.071 | -.032^*^ |  | .074 |  | 28% | 1.2 |
| Medicine | .266 | .257 | **.329** | -.026^**^ | .025^*^ | -.068 | -.043 | -.023^**^ | .016^*^ | .030 |  | 41.2% | 2.4 |
| Military Science | .208 | **.443** | .305 |  |  | -.063^*^ |  |  |  |  |  | 51.2% | 3.6 |
| Music | .239 | **.303** | .276 |  |  |  | -.126 |  |  |  |  | 40.2% | 1.4 |
| Natural History and Biology | **.315** | .263 | .312 |  |  | -.111 | -.068^**^ |  |  |  |  | 47.3% | 2.0 |
| Philosophy | **.281** | .239 | .213 | .071^**^ |  |  |  |  |  |  |  | 36.5% | 1.6 |
| Physics | .196 | **.269** | .188 | .079^**^ | .079^**^ | -.099 | -.084 |  |  | .065^**^ |  | 30.9% | 1.7 |
| Physiology | **.322** | .271 | .271 |  | .069^*^ | -.072 |  |  | .038^*^ |  |  | 47.5% | 2.8 |
| Political Sciences | **.323** | .289 | .249 | .032^*^ |  | -.076 | -.054 |  |  |  | .042 | 43.2% | 1.6 |
| Psychology | **.290** | .284 | .178 | .113 |  |  | -.065^**^ | -.061^**^ |  |  |  | 38.1% | 1.4 |
| Recreation and Leisure | .241 | **.274** | .212 |  |  |  | -.169 |  |  |  |  | 35.6% | 1.2 |
| Social Sciences | .281 | **.334** | .293 | .023^*^ |  | -.062 | -.061 | -.025^**^ | .029 |  |  | 44.6% | 1.8 |
| Zoology | **.386** | .164 | .354 |  |  | -.134 |  |  |  |  | .073^**^ | 48.4% | 2.1 |
| Average | .268 | .280 | .255 | .060 | .021 | -.100 | -.073 | -.051 | .025 | .050 | .045 | 38.5% | 1.8 |

All R^2^s are significant at p<0.001; All coefficients are significant at p<0.001 except *p<0.05; ** p<0.01.

Table 28. Statistically significant linear regression standardized coefficients (β) in 26 fields. The dependent metric is Library Electronic Holding. Only Goodreads Ratings is excluded from models due to causing collinearity.

| Fields | Scopus | GB | GU | GTR | GAR | Mendeley | Twitter | Facebook | Wiki | Blogs | News | R^2^ | R^2^ LEH/TLH |
| --- | --- | --- | --- | --- | --- | --- | --- | --- | --- | --- | --- | --- | --- |
| Agriculture | .165 | -.076^**^ | **.177** |  |  | .141 |  |  |  |  |  | 10.2% | 0.6 |
| Anthropology | .128 |  | **.165^**^** |  |  | -.077^*^ | -.083^*^ |  |  |  |  | 7.3% | 0.3 |
| Arts | **.195** |  |  |  | .140^**^ |  |  | -.068^*^ |  |  |  | 10.2% | 0.4 |
| Business and Economics | .146 | -.074 | **.236** | -.031^*^ |  | .082 |  |  |  | -.035^**^ |  | 9.3% | 0.4 |
| Chemistry | **.199** | -.227 | .148 |  |  | .143 | .075^*^ |  |  |  |  | 12.2% | 1.0 |
| Education | .177 | .096 | **.234** | -.073 |  | .069 |  |  |  |  |  | 14% | 0.6 |
| Engineering and Technology | .235 | -.161 | **.250** | -.057 |  | .174 | .083 | .029^**^ |  |  |  | 22.8% | 1.1 |
| Ethics and Religion | .162 | .080 | **.208** | -.075^**^ |  | -.107 | -.039^*^ |  |  |  |  | 9.6% | 0.4 |
| Geography | .070^*^ | -.067^*^ | **.204** |  |  | .151 |  |  |  |  |  | 6.9% | 0.4 |
| History | **.196** |  | .165 | -.037^*^ | .093 | -.120 | -.069 |  |  |  |  | 12.2% | 0.5 |
| Languages and Literature | .114 | -.038 | **.226** |  |  | -.084 | -.064 | -.024^*^ | .042 |  |  | 8.2% | 0.4 |
| Law | .144 | .034^*^ | **.177** |  |  | .059 | -.118 |  |  | -.074 | .037^*^ | 11.3% | 0.4 |
| Library Science and Bibliography | **.264** | -.194 | .161^*^ |  |  | -.145 | .094^*^ |  |  |  | -.114^**^ | 18.3% | 0.8 |
| Mathematics | .124 | -.066 | **.257** | -.069 |  | .192 | .041^*^ |  |  |  |  | 19.1% | 0.8 |
| Medicine | .166 | -.060 | .058 |  |  | **.187** |  |  |  | .023 |  | 8.8% | 0.5 |
| Military Science |  |  |  |  | **.114^*^** | .095^*^ |  |  |  |  |  | 5.3% | 0.4 |
| Music | **.188** |  | .165 |  |  | -.198 | -.132 |  |  |  |  | 16.3% | 0.6 |
| Natural History and Biology |  |  | **.219** |  | .075^*^ | .137 |  |  |  |  |  | 10% | 0.4 |
| Philosophy | .086^**^ | .047^*^ | **.160** |  | .083^*^ | -.087 |  | -.087^**^ |  |  |  | 9.1% | 0.4 |
| Physics | **.225** | -.078 | .198 |  |  | .145 | .070^**^ |  |  |  |  | 15.2% | 0.8 |
| Physiology | .13 |  |  |  |  | **.141** |  |  |  |  |  | 6.2% | 0.4 |
| Political Sciences | **.217** | .031^*^ | .143 |  | .067^**^ | -.058 | -.069 |  |  | -.057 |  | 12.6% | 0.5 |
| Psychology | **.265** |  |  |  | .073^*^ |  |  |  |  | -.062^*^ |  | 12% | 0.4 |
| Recreation and Leisure | .084^*^ |  | **.257** |  |  | -.082^*^ | -.142 |  | .094^**^ |  |  | 15.4% | 0.5 |
| Social Sciences | .184 | -.046 | **.217** |  |  | -.032^**^ | -.071 |  | .031^**^ |  |  | 10.9% | 0.4 |
| Zoology |  |  | **.355** |  |  | .101^*^ |  |  | .088^*^ |  |  | 10.3% | 0.4 |
| **Average** | **.168** | **-.050** | **.199** | **-.057** | **.092** | **.034** | **-.030** | **-0.038** | **.064** | **-.041** | **-.039** | **11.7%** | **0.5** |

All R^2^s are significant at p<0.001; All coefficients are significant at p<0.001 except *p<0.05; ** p<0.01.
